# Generic generation and manipulation of high-dimensional spin-orbit states in Hilbert space

**DOI:** 10.1038/s41467-026-74192-9

**Published:** 2026-06-24

**Authors:** Peijin Li, Ruizhe Zhao, Yang Cui, Guangzhou Geng, Junjie Li, Xifeng Ren, Yunkun Wu, Yongtian Wang, Shuang Zhang, Lingling Huang

**Affiliations:** 1 Beijing Engineering Research Center of Mixed Reality and Advanced Display, School of Optics and Photonics, Beijing Institute of Technology, Beijing, China; 2 Beijing National Laboratory for Condensed Matter Physics, Institute of Physics, Chinese Academy of Sciences, Beijing, China; 3 Laboratory of Quantum Information, University of Science and Technology of China, Hefei, China; 4 CAS Center for Excellence in Quantum Information and Quantum Physics, University of Science and Technology of China, Hefei, China; 5 Hefei National Laboratory, University of Science and Technology of China, Hefei, 230088 China; 6 New Cornerstone Science Laboratory, Department of Physics, University of Hong Kong, Hong Kong, China; 7 State Key Laboratory of Optical Quantum Materials, The University of Hong Kong, 999077 Hong Kong, China; 8 Department of Electrical and Electronic Engineering, The University of Hong Kong, 999077 Hong Kong, China; 9 Quantum Science Center of Guangdong-Hong Kong-Macao Greater Bay Area, 3 Binlang Road, Shenzhen, China; 10 Materials Innovation Institute for Life Sciences and Energy (MILES), HKU-SIRI Shenzhen, China

**Keywords:** Nanophotonics and plasmonics, Metamaterials

## Abstract

Light carries both spin (polarization) and orbital angular momentum. Combining these degrees of freedom produces hybrid spin–orbit states that live in a high-dimensional Hilbert space, offering greater information capacity and robustness for optical communication, quantum technologies, and metrology. However, generating arbitrary states in these spaces and characterizing them efficiently has remained difficult. Here we show a compact metasurface that generates arbitrary spin–orbit states in a four-dimensional Hilbert space, visualized on a Poincaré hypersphere, with straightforward scalability to higher dimensions. Using a tetratomic unit cell, the single-layer device precisely controls complex amplitude, phase, and polarization. We further introduce an efficient interferometric scheme that reconstructs the full density matrix of any *N*-dimensional spin–orbit state using only three interferograms. This approach uncovers an intrinsic spin–orbit parity order that governs the symmetry of projected intensity patterns, independent of the weighting of the eigenmodes, and enables controlled mode transformations through higher-order geometric phases. These advances establish a versatile platform for high-dimensional photonic technologies.

## Abstract (web summary)

The study presents a metasurface for generating high-dimensional spin-orbit light and a three interferogram scheme to characterize it, reveals a parity order that enforces symmetry, and shows mode switching through higher-order geometric phases.

## Introduction

The escalating demand for terabit-scale data transmission, ultra-dense data storage and robust, resource-efficient information processing is driving the adoption of high-dimensional optical carriers^1^. Photons, with their multiple degrees of freedom—such as polarization, spatial mode, wavelength, amplitude and phase serve as ideal information carriers for optical communication and quantum processing. Polarization states of photons carrying spin-angular momentum (SAM) constitute a two-dimensional Hilbert space spanned by left- and right-handed circularly polarized states (LCP and RCP). Additionally, the spatial mode of photons carries orbital angular momentum (OAM), which, in principle, supports an infinite number of eigenmodes^2^, enabling high-dimensional encoding alphabet^3–5^. When combined with spin angular momentum, scalar OAM can be elevated to vector vortex beams, generating non-separable spin-orbit states^6^. Such beams have found widespread applications in particle manipulation^7–9^, metrology^10^ and optical communication^11,12^. Furthermore, increasing the dimensionality of the states can bring many benefits over two-dimensional systems, including enhanced robustness against noise and eavesdropping^13,14^, higher information capacity^3,15^ and more efficient computing^16,17^.

Despite the unbounded nature of OAM and the broad application prospects of higher-dimensional states^18^, most works have remained confined to two-dimensional subspaces^19–21^. The fundamental obstacle lies in the complexity of the underlying Hilbert space: an *N*-dimensional state requires specification of 2(*N*−1) independent parameters^22^. The unitary evolution and manipulation of such states are governed by the SU(*N*) group, with (*N*^2^−1) degrees of freedom. This increasing complexity poses significant hurdles in generating and measuring high-dimensional states, and verifying their intrinsic properties. Additionally, a critical challenge is achieving coherent control and efficient detection of multiple optical degrees of freedom—including phase, polarization and OAM.

For the generation of high-dimensional spin-orbit states, cascaded optical elements—such as q-plates combined with polarizing beam splitters and wave plates—have been employed to generate four-dimensional mutually unbiased spin–orbit bases^23^. However, such setups cannot access the full Hilbert space and rely on external bulky components. Recent advances in on-chip synthesis, utilizing microcavities and metasurfaces, have demonstrated expansion of dimensionality and improved compatibility. For instance, two microcavities, controlled by thermal tuning and optical pumping, can generate higher-order spin-orbit states in a 4D Hilbert space through spatial overlapping with external elements^24^. However, this method is limited to opposite SAM and OAM pairs and does not allow arbitrary basis selection. While previous reports were only limited to two-dimensional spin-orbit states generations by using metasurfaces as mode converters^20,25–27^. By integrating quantum dots with moiré metasurfaces, quantum optical states featuring distinct spatial modes can be formed via the interference of spin-orbital waves. However, these states only occupy a two-dimensional subspace within the 4D Hilbert space^28^. Direct generation of an arbitrary hybrid spin-orbit state in a high-dimensional Hilbert space with a metasurface is still at an early stage, hindered by insufficient design degrees of freedom.

For state reconstruction, an *N*-dimensional spin–orbit state is defined by (2*N*−2) independent real parameters. The tomographic method requires at least (2*N*−1) projective measurements, becoming unpractical for large Hilbert spaces and prone to experimental noise when operating near this minimal bound^29^. Spin-dependent coordinate transformations or angular lenses can spatially demultiplex individual spin–orbit states and thus measure their relative intensities, but provide no information about the relative phases between eigenstates^30^. Interferometric approaches can retrieve the complex amplitudes of OAM spectra, yet they typically overlook the polarization degree of freedom, lacking a general and efficient method for characterizing arbitrary-dimensional spin–orbit Hilbert spaces^31^. Complete characterization of high-dimensional spin–orbit states is essential for emerging photonic technologies and fundamental studies, including spin–orbit generators and mode-conversion devices, decoding in optical-communication systems, and precise quantum state estimation in high-dimensional quantum information protocols.

**Fig. 1:**
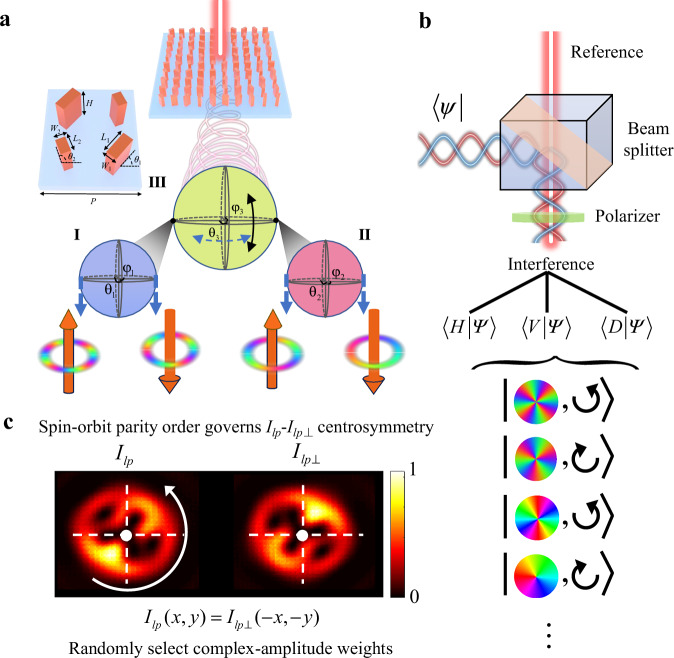
Generation, detection and parity order of high-dimensional spin–orbit states. **a** The generation of high-dimensional spin–orbit states via metasurface. **b** The schematic of the interferometry–projection–tomography reconstruction process. The generated state $|\psi \rangle$ interferes with a reference beam, and the interferograms recorded under the *H*, *V*, and *D* polarization projections are used for mode decomposition and to recover the high-dimensional spin-orbit state. **c** The concept of the spin–orbit parity order: the ordered arrangement of spin and OAM in the eigenmodes of the high-dimensional spin–orbit space induces a centrosymmetric feature in all states under linear polarization projections. As illustrated, the eigenmodes $|4,L\rangle,|3,R\rangle,|2,L\rangle,|-1,R\rangle$, corresponding to parity order parameter *P* = 1. A randomly selected state in this space, $|\psi \rangle=(-0.30+0.82i)|4,L\rangle -0.05i|3,R\rangle+(0.33+0.22i)|2,L\rangle+(0.17-0.21i)|-1,R\rangle$, exhibit centrosymmetric far-field projections under both *x*- and *y*-linear polarization bases.

In this work, we propose a generic approach for generating and detecting high-dimensional spin–orbit states using metasurfaces, as depicted in Fig. 1. By employing a tetratomic unit cell, we can precisely engineer the amplitude, phase, and polarization at each pixel through near-field interference, enabling the construction of arbitrary spin–orbit states with designated spatial modes in a four-dimensional Hilbert space. This approach generalizes to arbitrarily high-dimensional spin–orbit spaces by superposing basis OAM wavefronts with varying topological charges across corresponding polarization channels. We also develop a novel interferometry-projection-tomography scheme for full density matrix reconstruction of spin–orbit states in arbitrary dimensions, requiring only three interferograms—corresponding to *x*, *y*, and 45° polarization projections. This significantly reduces the measurement burden compared to traditional tomographies, which require (2*N*−1) measurements. Notably, certain high-dimensional spin–orbit states exhibit centrosymmetric features under linear polarization projections. We attribute this to an intrinsic spin–orbit parity order, determined by the parity of each eigenmode’s OAM and its spin orientation. This parity order governs the global symmetry of the projected intensity patterns, independent of the complex amplitude weighting of the eigenmodes, and provides a straightforward mechanism for quantum error correction. Furthermore, we demonstrate mode transformation governed by high-order geometric phases and reveal the geometric phases arising from metasurface rotations. These findings represent a significant advancement in overcoming the challenges of generating, characterizing, and understanding of intrinsic symmetry and geometry in high dimensions.

## Results

### Principle

Generally, a high-dimensional Hilbert space can be mapped onto a Poincaré hypersphere, which is composed of a set of nested Poincaré spheres. Figure 1 illustrates the schematic of the Poincaré hypersphere in a four-dimensional Hilbert space, with spin-orbit states serving as the basis. The general state in this four-dimensional Hilbert space can be denoted as:

1 $$\begin{array}{c}|\psi \rangle=\,\cos \left(\frac{{\theta }_{3}}{2}\right){e}^{-i\frac{{\varphi }_{3}}{2}}\left[\cos \left(\frac{{\theta }_{1}}{2}\right){e}^{-i\frac{{\varphi }_{1}}{2}}|{l}_{1},{\sigma }_{1}\rangle+\,\sin \left(\frac{{\theta }_{1}}{2}\right){e}^{i\frac{{\varphi }_{1}}{2}}|{l}_{2},{\sigma }_{2}\rangle \right]\\+\,\sin \left(\frac{{\theta }_{3}}{2}\right){e}^{i\frac{{\varphi }_{3}}{2}}\left[\cos \left(\frac{{\theta }_{2}}{2}\right){e}^{-i\frac{{\varphi }_{2}}{2}}|{l}_{3},{\sigma }_{3}\rangle+\,\sin \left(\frac{{\theta }_{2}}{2}\right){e}^{i\frac{{\varphi }_{2}}{2}}|{l}_{4},{\sigma }_{4}\rangle \right]\end{array}$$

Higher order Poincaré sphere(HOPS) I or (II) represents the linear combination of the eigenstates $|{l}_{1},{\sigma }_{1}\rangle$ and $|{l}_{2},{\sigma }_{2}\rangle$ (or $|{l}_{3},{\sigma }_{3}\rangle$ and $|{l}_{4},{\sigma }_{4}\rangle$), where${\sigma }_{i}=\pm 1$ represent LCP and RCP, respectively, *l* represents the topological charge of OAM. ${\theta }_{1},{\varphi }_{1}$ and ${\theta }_{2},{\varphi }_{2}$ are the polar angle and azimuthal angle of HOPS I and II, respectively. These two HOPS are nested within HOPS III, with each point on the HOPS III (polar angle *θ*_3_ and azimuthal angle *φ*_3_) representing a linear superposition of HOPS I and HOPS II. Using our method, any point on the hypersphere can be achieved by directing an x-polarized light beam onto the tailored metasurface.

Various types of beams can carry OAM with a characteristic phase front of exp(*ilφ*), such as Laguerre-Gaussian beams, Bessel beams, and Hypergeometric Gaussian beams. In this work, Hypergeometric Gaussian (HyGG) beams are chosen as carriers of OAM, which can be expressed in cylindrical coordinates as follows,

2 $$|l\rangle={{{\rm{HyGG}}}}_{-|l|,l}(r,\varphi,z)={C}_{l}{\zeta }^{\frac{-|l|}{2}}{(\zeta+i)}^{-(1-\frac{|l|}{2})}{\rho }^{|l|}{F}_{1}(\frac{|l|}{2},|l|+1;\frac{{\rho }^{2}}{\zeta (\zeta+i)}){e}^{-\frac{i{\rho }^{2}}{(\zeta+i)}}{e}^{il\varphi }$$

where *l* represents the topological charge of OAM, $\rho=\frac{r}{{w}_{0}}$, *φ*, $\zeta=\frac{z\lambda }{\pi {{w}_{0}}^{2}}$ are dimensionless cylindrical coordinates, *w*_0_ is waist radius, *F*_1_ is a confluent hypergeometric function. This beam possesses self-reconstruction and self-focusing properties^32^. At *z* = 0, the function has the form ${|l\rangle }_{z=0}=C{e}^{-{\rho }^{2}+il\varphi }$. Therefore, a HyGG beam can be generated by a Gaussian beam passing through an optical component with a phase profile of *lφ*.

To generate high-dimensional spin-orbit states, the amplitude, phase, and polarization of the incident light must be precisely modulated. Conventional devices such as q plates and spatial light modulators are limited in this simultaneous control. In contrast, metasurfaces provide a versatile platform to meet these requirements. For a four-dimensional spin-orbit mode converter, the Jones matrix of the metasurface in the circularly polarized basis can be expressed as:

3 $$\begin{array}{c}J=\,\cos \left(\frac{{\theta }_{3}}{2}\right){e}^{-i\frac{{\varphi }_{3}}{2}}\left[\cos \left(\frac{{\theta }_{1}}{2}\right){e}^{-i\frac{{\varphi }_{1}}{2}}|{l}_{1},{\sigma }_{1}\rangle \langle 0,-{\sigma }_{1}|+\,\sin \left(\frac{{\theta }_{1}}{2}\right){e}^{i\frac{{\varphi }_{1}}{2}}|{l}_{2},{\sigma }_{2}\rangle \langle 0,-{\sigma }_{2}|\right]\\+\,\sin \left(\frac{{\theta }_{3}}{2}\right){e}^{i\frac{{\varphi }_{3}}{2}}\left[\cos \left(\frac{{\theta }_{2}}{2}\right){e}^{-i\frac{{\varphi }_{2}}{2}}|{l}_{3},{\sigma }_{3}\rangle \langle 0,-{\sigma }_{3}|+\,\sin \left(\frac{{\theta }_{2}}{2}\right){e}^{i\frac{{\varphi }_{2}}{2}}|{l}_{4},{\sigma }_{4}\rangle \langle 0,-{\sigma }_{4}|\right]\\=\left(\begin{array}{cc}0 & {a}_{rl}{e}^{i{\varphi }_{rl}}\\ {a}_{lr}{e}^{i{\varphi }_{lr}} & 0\end{array}\right)\end{array}$$

where *l* = 0 corresponds to a Gaussian beam. When a horizontally polarized light $\frac{1}{\sqrt{2}}(|R\rangle+|L\rangle )$ is incident onto the metasurface, the transmitted light field exhibits the designed high-dimensional spin-orbit state. To implement the corresponding Jones matrix, the complex amplitudes of the two polarization conversion channels need to be independently controlled. By using tetramers to form the super cells, one can realize complex amplitude modulation in the orthogonal circular polarization channels, while the OAM of the co-polarized channels remains zero (See Supplementary Note 1). Meanwhile, higher dimensionality can be realized by superposing orthogonal spin-orbit bases with different weights using a single metasurface that implements the corresponding Jones matrix. Hence, such a method is universal and scalable.

Based on the method described above, we designed four metasurfaces (Sample #1, #2, #3, #4) to realize different spin-orbit bases ($|{l}_{i},{\sigma }_{i}\rangle$) on HOPS, corresponding to different modulation parameters (*θ*_1_, *φ*_1_, *θ*_2_, *φ*_2_, *θ*_3_, *φ*_3_). The four hybrid spin-orbit states are as follows:

4 $$|{\varPsi }_{1}\rangle=	 \frac{1}{2}(|2,L\rangle+|-2,R\rangle+i|-1,L\rangle+i|1,R\rangle )\\ |{\varPsi }_{2}\rangle=	 \frac{\sqrt{2}}{2}[\cos (\frac{\pi }{8})(|2,L\rangle+|-2,R\rangle )+\,\sin (\frac{\pi }{8}){e}^{i\frac{\pi }{4}}(|-1,L\rangle+|1,R\rangle )]\\ |{\varPsi }_{3}\rangle=	 \frac{1}{2}(|4,L\rangle+|3,R\rangle+|2,L\rangle+|-1,R\rangle )\\ |{\varPsi }_{4}\rangle=	 \frac{\sqrt{2}}{2}[\cos (\frac{\pi }{8})(|4,L\rangle+|3,R\rangle )+\,\sin (\frac{\pi }{8}){e}^{i\frac{\pi }{4}}(|2,L\rangle+|-1,R\rangle )]$$

The first two spin–orbit fields consist of opposite SAM and OAM pairs, corresponding to the north and south poles on HOPS I and HOPS II, respectively. $|{\varPsi }_{3}\rangle$ and $|{\varPsi }_{4}\rangle$ represent OAM states at the poles of HOPS I and HOPS II with arbitrary OAM numbers ${l}_{1}\ne {l}_{2}\ne {l}_{3}\ne {l}_{4}$. This flexible configuration reveals hidden spatial symmetries and an intrinsic spin–orbit parity order, observable in such asymmetrically structured high-dimensional states—a phenomenon not reported previously to our knowledge. This design paradigm significantly overcomes the limitations of microcavity-based approaches, which are typically constrained to generate symmetric SAM–OAM pairs.

### General framework for high-dimensional spin-orbit state characterization and metasurface verification

**Fig. 2:**
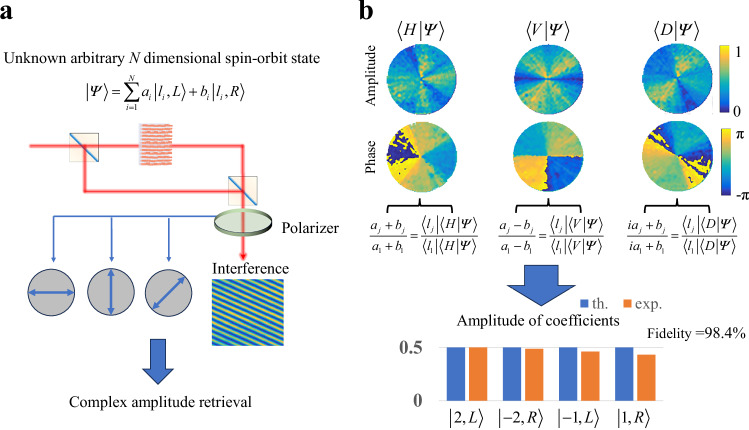
Interferometry-projection-tomography reconstruction scheme for high-dimensional spin–orbit states. **a** A schematic illustrating the acquisition of complex amplitudes for three projection channels via interferometry and polarization projection. **b** The retrieved complex amplitudes are decomposed into OAM modes, and the resulting set of equations is solved via the least-squares method to reconstruct the unknown high-dimensional state. The table in the lower panel presents the relative amplitude contributions of different eigenmodes in the high-dimensional state generated by Sample #1, with theoretical predictions shown in blue and experimentally retrieved values in orange.

To efficiently characterize four-dimensional spin–orbit states generated by metasurfaces, we propose a hybrid interferometry-projection-tomography scheme that integrates projection measurement with off-axis interferometry, enabling the reconstruction of the density matrix $\rho=|\psi \rangle \langle \psi |$ for arbitrary high-dimensional spin–orbit states using only three interferograms (See “Methods”). The weights of the basic components $|\varPsi \rangle={\sum }_{i=1}^{N}{a}_{i}|{l}_{i},L\rangle+{b}_{i}|{l}_{i},R\rangle$ that span the Hilbert space can thus be determined. As shown in Fig. 2, the unknown state is projected onto horizontal ∣H⟩, vertical ∣V⟩, and diagonal polarization states ∣D⟩, and interfered with a reference beam. The resulting interferograms are processed via Fourier transform (see Supplementary Note 2) to retrieve the complex amplitude distributions under the three polarization projections. As an example, Fig. 2b (upper panel) shows the retrieved amplitude and phase distributions for Sample #1. These fields are further decomposed into OAM eigenmodes, yielding a system of linear equations with coefficients *a*_*i*_ and *b*_*i*_ as variables. By solving this linear system, the complex coefficients of the constituent eigenstates can be determined, allowing for the reconstruction of the full density matrix. The bar chart in Fig. 2b (lower panel) compares the experimentally retrieved relative amplitudes of the eigenmodes with the theoretical predictions for Sample #1, showing excellent agreement.

**Fig. 3:**
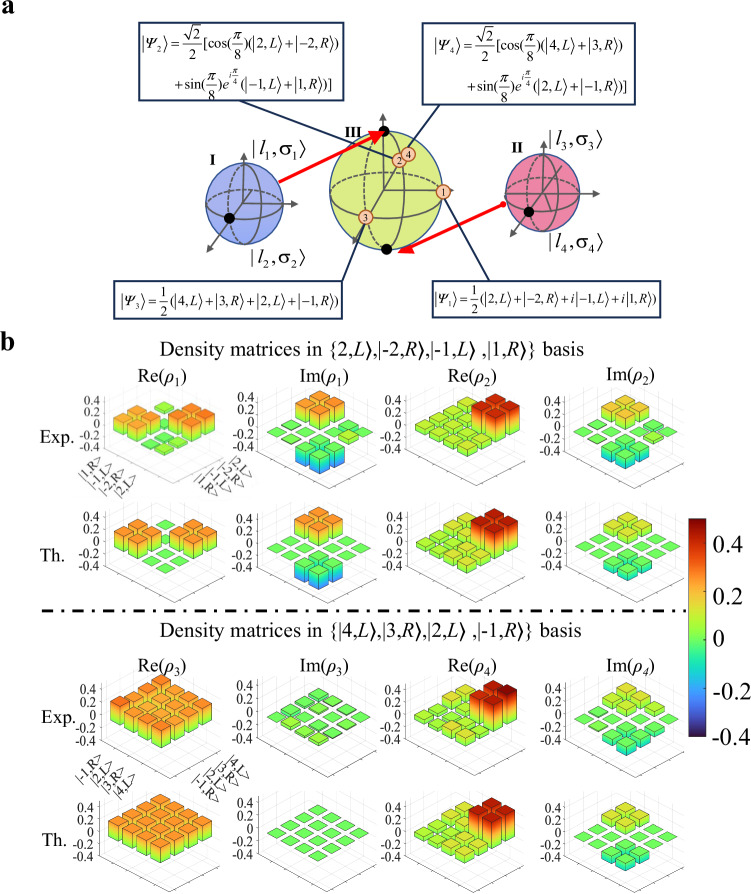
Experimental verification of designed metasurfaces. **a** The locations of four selected spin-orbit states on the HOPS. **b** The theoretically predicted and experimentally retrieved density matrices $\rho=|\varPsi \rangle \langle \varPsi |$ of the four states. The fidelities are 98.4%, 98.4%, 99.0% and 98.3%, respectively, with an average high fidelity of 98.5%, validating the capability of the generation of the high-dimensional spin–orbit states using metasurface.

Using this approach, we further characterize four spin–orbit states generated by distinct metasurface designs. Figure 3a illustrates their corresponding geometric locations on the Poincaré hypersphere. The design parameters (*θ*_1_, *φ*_1_, *θ*_2_, *φ*_2_, *θ*_3_, *φ*_3_) are as follows: Sample #1: (π/2, 0, π/2, 0, π/2, π/2); Samples #2 and #4: (π/2, 0, π/2, 0, π/4, π/4), but the two samples have different eigenmodes; Sample #3: (π/2, 0, π/2, 0, π/2,0). Figure 3b compares the experimentally reconstructed and target density matrices of these four spin–orbit states, achieving an average fidelity of 98.5%. This high fidelity confirms the effectiveness of our method in generating and reconstructing high-dimensional spin–orbit states.

### Spin–orbit parity order

We further identify a hidden parity structure in the high-dimensional spin–orbit Hilbert space, which determines whether the state exhibits central symmetry. Specifically, for a state $|\varPsi \rangle={\sum }_{{{\rm{i}}}=1}^{N}{a}_{i}|{l}_{i},{\sigma }_{i}\rangle$, if all eigenstates satisfy that both the parity of OAM $p({l}_{i})$ (where parity is +1 for even and −1 for odd) and SAM *σ*_*i*_ are the same (or opposite), the resulting superposition state exhibits a centro-symmetric far-field intensity distribution under linear polarization projection. This behavior resembles long-range spin alignment in ferromagnets, where microscopic interactions lead to macroscopic order. However, unlike ferromagnetic systems driven by exchange interactions, the coherence symmetry observed here arises from intrinsic spin–orbit parity correlations in the high-dimensional spin-orbit Hilbert space. We define this hidden structure as the spin–orbit parity order, and introduce a corresponding order parameter to quantify it:

5 $$P={\sum }_{{{\rm{i}}}=1}^{N}{|{a}_{i}|}^{2}\cdot p({l}_{i})\cdot {\sigma }_{i}$$

A value of *P* = ±1 indicates complete order, corresponding to centrosymmetric projections, while *P* = 0 means complete disorder. This parameter captures the global parity correlation between the spin and orbital components and is directly linked to the emergence of symmetry in the far field. It is also natural to classify the high-dimensional spin–orbit states according to the spin–orbit parity order parameter, into the parity-ordered region (*P* = ±1) and parity-disordered region (∣P∣<1).

**Fig. 4:**
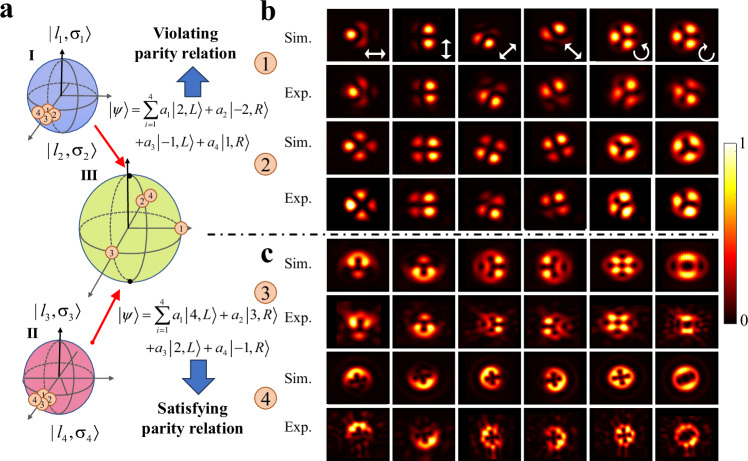
Spin–orbit parity order and centrosymmetry in high-dimensional states. **a** The fabricated metasurfaces are categorized into two types based on the spin–orbit parity order. **b** Simulated and experimentally measured far-field intensity projections of Samples #1 and #2 under horizontal, vertical, diagonal, anti-diagonal, left- and right-circular polarization. Due to fabrication-induced dispersion shifts, Sample #1 is measured at 900 nm and Sample #2 at 830 nm. **c** Far-field simulations and measurements for Samples #3 and #4 under various polarization projections. In both cases, we remove spin–orbit components with unwanted crosstalk arising from fabrication imperfections (see Supplementary Note 5). Measurements are conducted at 840 nm for both samples. A clear centrosymmetry emerges only for states with parity order *P* = 1.

In our previous design, as shown in Fig. 4a, Sample #1 and Sample #2 have spin–orbit parity order parameter *P* = 0, belonging to the disordered space. In contrast, Samples #3 and Samples #4 belong to the ordered space, where the order parameter *P* = 1. Moreover, it is noteworthy that for the eigen-basis chosen in Samples #3 and #4, the order parameter is a Hilbert-space invariant, remaining unchanged under different *a*_*i*_. Accordingly, the associated symmetry emerges as an intrinsic and invariant property of the spin–orbit Hilbert space. Figure 4b presents the experimentally measured and theoretically predicted far-field intensity distributions for the high-dimensional spin–orbit states generated by the two types of samples, projected onto different polarization channels. In the disordered case, the far-field projections onto linear polarization bases—whether horizontal/vertical or diagonal/anti-diagonal—exhibit no central symmetry, regardless of whether the projected states lie on the equator of the Poincare hypersphere or at other locations.

In contrast, Fig. 4c shows the results for the ordered space. For both Sample #3 and Sample #4, clear central symmetry emerges in the far-field intensity distributions when projected onto either the horizontal/vertical or diagonal/anti-diagonal linear polarization bases after eliminating certain crosstalk (see Supplementary Note 5 for details). By comparison, such symmetry does not appear under projection onto general elliptical polarization bases, as exemplified by the LCP and RCP channels in Fig. 4c. The slight discrepancies between theory and experiment are attributed to residual disordered high-order spin–orbit eigenstates present in the experiment.

### Higher-order Pancharatnam−Berry (PB) phase-controlled mode transformation

Furthermore, we demonstrate a higher-order Pancharatnam–Berry (PB) phase–controlled mode transformation in our metasurface system. The geometric phase refers to a phase accumulated when a system’s initial state undergoes an adiabatic evolution along a closed loop in its parameter space, and is determined by the geometry of the trajectory. In optics, this is manifested as the PB phase acquired when a polarization state traces a closed loop on the Poincaré sphere. The higher-order PB phase arises from the adiabatic evolution of a two-dimensional spin–orbit state on a higher-order Poincaré sphere, where the north and south poles represent the two spin–orbit eigenstates.

**Fig. 5:**
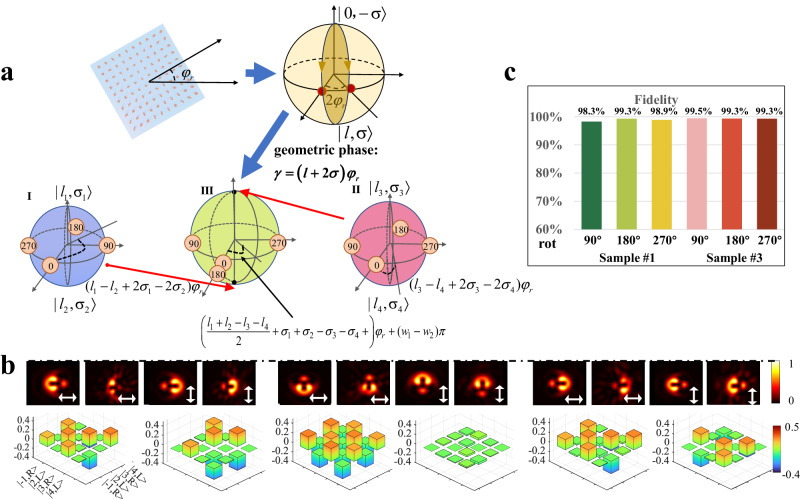
Higher-order Pancharatnam−Berry (PB) phase-controlled mode transformation. **a** Metasurface rotation modifies the evolution trajectory of the spin–orbit state, leading to the accumulation of a higher-order geometric phase. This phase shift alters the relative phases between spin–orbit eigenstates and results in a longitude-like rotation on three hybrid-order Poincaré spheres (HOPS I, II, and III), thereby switching the mode of the high-dimensional spin–orbit state. The labels 0, 90, 180, and 270 represent the geometric positions of the high-dimensional state after Sample #3 is rotated by 0°, 90°, 180°, and 270°, respectively. **b** Simulated and experimentally measured far-field intensity distributions and reconstructed density matrices of Sample #3 under metasurface rotations of 90°, 180°, and 270°, respectively. **c** Fidelities between the measured and theoretically predicted density matrices for Sample #1 and Sample #3. The average fidelity reaches 99%, validating the predictive accuracy of our model.

We consider a linearly polarized beam incident on a metasurface, which can be decomposed into LCP and RCP. When the state $|0,-\sigma \rangle$ impinges on the metasurface, it evolves into the state$\sum {c}_{i}|{l}_{i},\sigma \rangle$. Since the entire system operates in the linear optical regime, the light–metasurface interaction can be decomposed into multiple independent two-dimensional subspaces. In each subspace, the incident state $|0,-\sigma \rangle$ and output state $|l,\sigma \rangle$ span a two-dimensional spin–orbit Hilbert space, which can be visualized on a hybrid-order Poincaré sphere, where the poles correspond to the incident and output states, as illustrated by the yellow sphere in Fig. 5a. After the state $|0,-\sigma \rangle$ interacts with the metasurface, it evolves along a specific meridian toward the output state $|l,\sigma \rangle$. When the metasurface is rotated by ${\varphi }_{r}$, the evolution trajectory rotates $2{\varphi }_{r}$ accordingly, resulting in a geometric phase shift $\gamma=(l+2\sigma ){\varphi }_{r}$ (see Supplementary Note 7 for detailed derivation). Compared to the unrotated case, each eigenstate in the high-dimensional spin–orbit superposition acquires a distinct higher-order PB phase upon metasurface rotation. As a result, the overall spin–orbit state undergoes a transformation, manifested as a relative phase shift between the eigenstates. Geometrically, this corresponds to a longitude rotation on the Poincaré hyperspheres (HOPS Ⅰ, HOPS Ⅱ, and HOPS Ⅲ), as illustrated in Fig. 5a.

Assuming the initial state is parametrized on the Poincaré hypersphere as $[{\varphi }_{1},{\theta }_{1},{\varphi }_{2},{\theta }_{2},{\varphi }_{3},{\theta }_{3}]$, then after the metasurface rotation by ${\varphi }_{r}$, the transformed state can be written as:

6 $$	 \Bigg[{\varphi }_{1}+({l}_{1}-{l}_{2}+2{\sigma }_{1}-2{\sigma }_{2}){\varphi }_{r},{\theta }_{1},{\varphi }_{2}+({l}_{3}-{l}_{4}+2{\sigma }_{3}-2{\sigma }_{4}){\varphi }_{r},{\theta }_{2},\\ 	 {\varphi }_{3}+\left(\frac{{l}_{1}+{l}_{2}-{l}_{3}-{l}_{4}}{2}+{\sigma }_{1}+{\sigma }_{2}-{\sigma }_{3}-{\sigma }_{4}\right){\varphi }_{r}+({w}_{1}-{w}_{2})\pi,{\theta }_{3}\Bigg]\\ 	 {{\rm{where}}}\,\,{w}_{1}=round\left[\left|\frac{({l}_{1}-{l}_{2}+2{\sigma }_{1}-2{\sigma }_{2}){\varphi }_{r}}{2\pi }\right|\right],{w}_{2}=round\left[\left|\frac{({l}_{3}-{l}_{4}+2{\sigma }_{3}-2{\sigma }_{4}){\varphi }_{r}}{2\pi }\right|\right]$$

*round*[] represents the round down integer. *w*_1_ and *w*_2_ denote the number of full 2π rotations around HOPS I and HOPS II, respectively. These two terms originate from the double covering of the SU(2) to SO(3) mapping, where a full 2π rotation on HOPS I (or II) leads to a relative phase shift of π. This phase manifests on HOPS III as a longitude rotation of $({w}_{1}-{w}_{2})\pi$.

Importantly, apart from the parameter transformation on HOPS, there exists an additional global phase, which can be written as:

7 $${\varphi }_{global}=-\left(\frac{{l}_{1}+{l}_{2}+{l}_{3}+{l}_{4}+2{\sigma }_{1}+2{\sigma }_{2}+2{\sigma }_{3}+2{\sigma }_{4}}{4}\right){\varphi }_{r}-\frac{{w}_{1}+{w}_{2}}{2}\pi -{w}_{3}\pi$$

${w}_{3}=round\left[\left|\left(\frac{{l}_{1}+{l}_{2}-{l}_{3}-{l}_{4}}{2}+{\sigma }_{1}+{\sigma }_{2}-{\sigma }_{3}-{\sigma }_{4}\right){\varphi }_{r}+\left({w}_{1}-{w}_{2}\right)\pi \right|\right]$. Such a nontrivial global phase corresponds to an evolution path in a high-dimensional Hilbert space (See Supplementary Note 7). Its first term represents the averaged higher-order PB phase contributed by the geometric rotation of individual spin–orbit eigenstates, the second term arises from the SU(2) → SO(3) double-cover topology between HOPS I and II, and the third term corresponds to the accumulated phase associated with the global rotation of the nested Poincaré hypersphere (HOPS III). This global term reveals a generalized Pancharatnam–Berry phase, offering deep insights into the intrinsic geometric nature of high-dimensional spin–orbit light fields.

In Fig. 5a, the labels 0, 90, 180, and 270 indicate the positions of the high-dimensional state on the HOPS when Sample #3 is rotated by 0°, 90°, 180°, and 270°, respectively. The nonlinear variation of the azimuthal angle on HOPS III under metasurface rotation is a direct consequence of the influence of *w*_1_ and *w*_2_. Figure 5b shows the far-field intensity distributions and experimentally reconstructed density matrices for Sample #3, where the initial state undergoes metasurface rotations of 90°, 180°, and 270°, respectively, with projections onto linear polarization bases. The density matrix varies significantly by rotating the metasurface. The far-field projection also rotates accordingly, while preserving the intensity profiles, indicating an intrinsic transformation of such higher order spin-orbit modes. Figure 5c summarizes the fidelities of Sample #1 and Sample #3 between the experimentally reconstructed density matrices and those calculated from Eq. (6), showing excellent agreement and thus validating our theoretical model.

We also derive the evolution of high-dimensional spin–orbit states with respect to variations in the incident polarization state (Supplementary Note 8). Due to the interaction of the Jones matrix, the spin-orbit state is transformed into a new one, with different projections in 2D subspaces. Notably, unlike metasurface rotation, the azimuthal angle on at least one of the Poincaré spheres remains unchanged. This is because the polarization state of the input beam only modifies the relative complex amplitudes between spin components. However, when the SAM at the north and south poles of a given HOPS are identical, variations in polarization have no effect on that sphere. In the case of the four samples used in this work, the azimuthal angle on HOPS III remains invariant under changes in input polarization.

## Discussion

We have demonstrated the generation of spin–orbit states in a four-dimensional Hilbert space using metasurface. Our approach allows for straightforward design of higher-dimensional spin–orbit states by incorporating additional spin–orbit eigen-components into the Jones matrix formalism. We further demonstrate a representative eight-dimensional case for supporting this scalability, while the detection scheme remains the same by using only three interferograms (see Supplementary Note 9). Meanwhile, increasing dimensionality places more stringent requirements on metasurface design accuracy, fabrication precision and control of near field coupling.

Moreover, this framework can be extended to include other optical degrees of freedom—such as wavelength, path—enabling the construction of more complex entangled states across a broader Hilbert space. Our method for generating and characterizing high-dimensional spin–orbit states can, in principle, multiply the bandwidth of existing OAM-based optical-communication links^33,34^. In addition, high-dimensional spin–orbit states provide a natural platform for parallel, multi-parameter and higher-precision metrology^35^; they enable optical encryption in an enlarged Hilbert space, greatly increasing key complexity and decoding difficulty; they allow holographic multiplexing to move beyond orthogonal OAM channels toward non-orthogonal, high-dimensional spin–orbit holography, expanding the display and data-storage capacity. Access to richer spin–orbit space broadens the toolbox of strong-field vortex lasers, opening the door to more intricate light–plasma interactions and potentially novel physical phenomena^36^. These capabilities highlight the broad applicability of the present approach across photonic communications, sensing, security, holography, high-field laser physics and beyond.

Additionally, we identify a novel spin–orbit parity order, which unveils a hidden symmetry in the high-dimensional Hilbert space. Notably, for fully ordered states—that is, when *P* = ±1, the emergent symmetry is preserved under arbitrary change of the constituent eigenmode complex amplitudes. This parity structure holds potential for optical parity checking, quantum error correction, and even photonic simulation of quantum many-body systems, owing to its analogy with spin–orbital interactions in condensed matter systems. The discovery of our parity order indicates the existence of ordered structures in high-dimensional spin-orbit space. Intriguingly, we demonstrate that metasurface-induced higher-order geometric phases can be used to manipulate the transformation of spin–orbit states, highlighting metasurfaces as powerful tools for probing and engineering geometric phases in high-dimensionality systems.

Furthermore, when integrated with quantum light sources, this metasurface-based platform can be extended to the quantum regime, offering promising routes toward high-dimensional quantum key distribution, quantum communication, and quantum teleportation. Our proposed hybrid interferometric–projective tomography scheme is compatible with two-photon interference setups and can enable fast reconstruction of high-dimensional spin–orbit entangled states^37^. When generalized to multiphoton systems, the spin–orbit parity order could serve as a guiding principle for exploring symmetry, entanglement, and topology in high-dimensional multiphoton Hilbert spaces. When extended to entangled photon inputs, such platforms may enable access to richer topological and geometric phenomena.

## Methods

### Design of metasurfaces

A tetramer unit is used to satisfy the requirements for complex amplitude modulation in polarization conversion channels. The unit structure is composed of a SiO_2_ substrate and a tetramer made of Si nanopillars. The diagonal nanopillars share identical geometric parameters, and the anti-diagonal pair shares a different set. The period of the unit cell is *P* = 1100 nm, and the height of the Si nanopillars is 600 nm. In our design, each nanopillar functions as a half-wave plate. Its Jones matrix in the circular polarization basis can be written as: $${T}_{cir}=\left(\begin{array}{cc}0 & {e}^{i{\varphi }_{xx}-2\alpha }\\ {e}^{i{\varphi }_{xx}+2\alpha } & 0\end{array}\right)$$, where ${\varphi }_{x}$ denotes the phase delay along the long axis of the nanopillar, and *α* is the geometric rotation angle. By varying the length, width, and rotation angle, such a silicon nanopillar can independently modulate the phase of the two polarization conversion channels. When four silicon pillars are combined and placed together, interference occurs between adjacent nanostructures, giving rise to complex amplitude responses. The total response of the tetramer can be expressed as:

$${J}_{{{\rm{unit}}}}=\frac{1}{4}{\sum }_{i=1}^{4}{J}_{i}=\frac{1}{2}\left(\begin{array}{cc}0 & {e}^{i{\varphi }_{1,xx}-2{\alpha }_{1}}\\ {e}^{i{\varphi }_{1,xx}+2{\alpha }_{1}} & 0\end{array}\right)+\frac{1}{2}\left(\begin{array}{cc}0 & {e}^{i{\varphi }_{2,xx}-2{\alpha }_{2}}\\ {e}^{i{\varphi }_{2,xx}+2{\alpha }_{2}} & 0\end{array}\right)$$

This enables independent control of the complex amplitudes for the two polarization conversion channels, denoted by *T*_*lr*_ and *T*_*rl*_. Where *α*_1_ and *α*_2_ are the rotation angles of the diagonal and anti-diagonal nanopillars. ${\varphi }_{1,xx}$ and ${\varphi }_{2,xx}$ represent the phase delays along the *x-*directions of the two pairs of nanostructures. Therefore, we can have

$$\left\{\begin{array}{c}{\varphi }_{1,xx}=\frac{{\varphi }_{rl}+{\varphi }_{lr}+{\cos }^{-1}({a}_{rl})+{\cos }^{-1}({a}_{lr})}{2}\\ {\varphi }_{2,xx}=\frac{{\varphi }_{rl}+{\varphi }_{lr}-{\cos }^{-1}({a}_{rl})-{\cos }^{-1}({a}_{lr})}{2}\\ {\alpha }_{1}=\frac{-{\varphi }_{rl}+{\varphi }_{lr}-{\cos }^{-1}({a}_{rl})+{\cos }^{-1}({a}_{lr})}{4}\\ {\alpha }_{2}=\frac{-{\varphi }_{rl}+{\varphi }_{lr}+{\cos }^{-1}({a}_{rl})-{\cos }^{-1}({a}_{lr})}{4}\end{array}\right.$$

### Interferometry-projection-tomography scheme

As illustrated in Fig. 2, the complex amplitude distribution of the high-dimensional spin–orbit state$|\varPsi \rangle={\sum }_{i=1}^{N}{a}_{i}|{l}_{i},L\rangle+{b}_{i}|{l}_{i},R\rangle$ under horizontal ∣H⟩, vertical ∣V⟩, and diagonal ∣D⟩ projection can be experimentally retrieved through interferometric measurements. Take horizontal polarization projection as an example, horizontal polarization projection state can be written as ${E}_{H}(x,y)=\langle H|\varPsi \rangle={\sum }_{i=1}^{N}\frac{1}{\sqrt{2}}({a}_{i}+{b}_{i})|{l}_{i}\rangle$. ${E}_{H}(x,y)$ denotes the spatial complex amplitude of the high-dimensional state under horizontal polarization projection. It can be directly represented as ${E}_{H}(x,y)={\sum }_{i=1}^{N}{c}_{H,i}|{l}_{i}\rangle$, where ${c}_{H,i}$ is the complex amplitude coefficient corresponding to the OAM eigenstates$|{l}_{i}\rangle$. Without loss of generality, we assume that ${a}_{1}\ne 0$, and obtain:

$$\frac{{a}_{i}/{a}_{1}+{b}_{i}/{a}_{1}}{1+{b}_{1}/{a}_{1}}=\frac{{c}_{H,i}}{{c}_{H,1}},i={2..}.N$$

Similarly, the equations corresponding to vertical and diagonal polarization projections can be constructed as $\frac{{a}_{i}/{a}_{1}-{b}_{i}/{a}_{1}}{1-{b}_{1}/{a}_{1}}=\frac{{c}_{V,i}}{{c}_{V,1}}$ and$\frac{i{a}_{i}/{a}_{1}+{b}_{i}/{a}_{1}}{i+{b}_{1}/{a}_{1}}=\frac{{c}_{D,i}}{{c}_{D,1}},i={\mathrm{2..}}.N$. ${c}_{V,i}$ and ${c}_{D,i}$ denote the complex amplitude coefficients of each OAM eigenmode under vertical and diagonal polarization projections, respectively. In total, there are (4*N*−2) unknowns and (6*N*-6) linear equations, which can be solved using the least-squares method to retrieve the complex coefficients of the high-dimensional spin–orbit state, and thus reconstruct its density matrix. Although our method is demonstrated with classical light fields, it holds promise for fast reconstruction of quantum high-dimensional spin–orbit states when combined with two-photon interference techniques^37^.

## Supplementary information

Supplementary information

Peer Review file

## Data Availability

All data are available in the main text and supplementary information.

## Code availability

The codes that support the findings of this study are available for reasonable request from the corresponding authors.
