# Supplementary material for: Generic generation and manipulation of high-dimensional spin-orbit states in Hilbert space
Source: Nat Commun. 2026 Jun 24;17:7936. doi: 10.1038/s41467-026-74192-9 (PMC13448707; doi:10.1038/s41467-026-74192-9)
Supplement: Supplementary file 1 [file 41467_2026_74192_MOESM1_ESM.pdf]

Supplementary Information for Generic generation and manipulation of high-dimensional spin-orbit states in Hilbert space

Peijin Li[1], Ruizhe Zhao[1], Yang Cui[1], Guangzhou Geng[2], Junjie Li[2], Xifeng Ren[3,4,5], Yunkun Wu[3,4,5], Yongtian Wang[1], Shuang Zhang[6,7,8,9,10]*, Lingling Huang[1]*

1: Beijing Engineering Research Center of Mixed Reality and Advanced Display, School of Optics and Photonics, Beijing Institute of Technology, Beijing, China.
2: Beijing National Laboratory for Condensed Matter Physics, Institute of Physics, Chinese Academy of Sciences, Beijing, China.
3: Laboratory of Quantum Information, University of Science and Technology of China, Hefei, China.
4: CAS Center for Excellence in Quantum Information and Quantum Physics, University of Science and Technology of China, Hefei, China.
5: Hefei National Laboratory, University of Science and Technology of China, Hefei 230088, China
6: New Cornerstone Science Laboratory, Department of Physics, University of Hong Kong, Hong Kong, China.
7: State Key Laboratory of Optical Quantum Materials, The University of Hong Kong, 999077 Hong Kong, China
8: Department of Electrical and Electronic Engineering, The University of Hong Kong, 999077 Hong Kong, China
9: Quantum Science Center of Guangdong-Hong Kong-Macao Great Bay Area, 3 Binlang Road, Shenzhen, China
10: Materials Innovation Institute for Life Sciences and Energy (MILES), HKU-SIRI, Shenzhen, China

The list of Supplementary Notes

1. Design of metasurface

2. Interferometry-projection-tomograph method for arbitrary high-dimensional spin-orbit state density matrix reconstruction

3. Experimental setup for the interferometric reconstruction of density matrix

4. Experimental setup for far-field intensity imaging and polarization projection

5. Removal of crosstalk terms

6. The relationship between spin-orbit order parameter and centrosymmetry of linear polarization projection

7. Analysis of high-dimensional spin-orbit states transformation induced by higher-order geometric phase

8. Effect of changing incident polarization

9. Scalability to higher dimension

1.Design of metasurface

We begin by performing a two-dimensional parameter sweep of a single nanopillar, as indicated by the black dashed line in Figure S1(a), using the rigorous coupled-wave analysis (RCWA) under periodic boundary conditions. The height H of the nanopillar is fixed at 600 nm, and the lattice period is set to 550 nm. The rectangular cross-section is scanned over lengths and widths ranging from 75 nm to 315 nm. The amplitude T_{xx} and phase ϕ_{xx} distributions of the transmitted light component E_{xx} corresponding to various geometries are plotted in Figure S1(c) and (d). Due to symmetry, the amplitude and phase response of E_{yy} can be obtained via a 90° rotation of the structures and are thus equivalent to E_{xx}. The results confirm that a complete $0 \sim 2\pi$ phase coverage with nearly uniform amplitude is achievable across the swept design space.

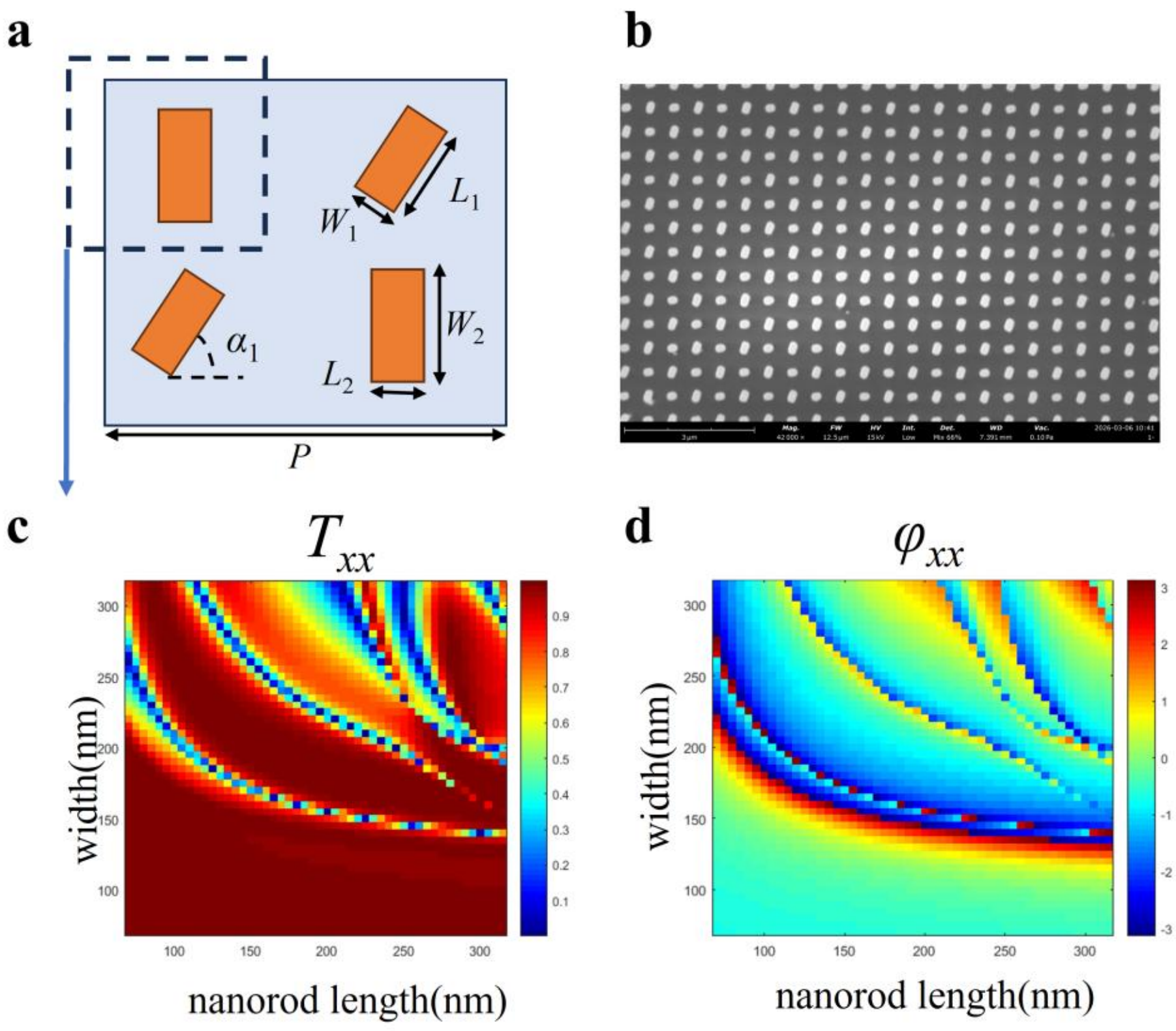

Figure S1. Numerical simulation of meta-atoms within the tetramer. **a.** Schematic of the tetramer unit cell composed of four nanopillars. The black dashed box represents the nanopillar to be numerically simulated. **b.** The scanning electron microscopy image of Sample #1, with the scale bar of 3 μm. **c.** Simulated amplitude response T_{xx} as a function of nanopillar length and width. **d.** Corresponding phase response ϕ_{xx}. The design space offers full $0 \sim 2\pi$ phase coverage and high transmission efficiency.

When a single nanopillar is rotated by an angle α, its Jones matrix in the linear polarization basis becomes: $J(\alpha) = R(-\alpha)\begin{pmatrix} e^{i\phi_x} & 0 \\ 0 & e^{i\phi_y} \end{pmatrix}R(\alpha)$, where $R(\alpha)$ is the standard rotation matrix, and ϕ_x, ϕ_y are the phase shifts along the principal axes. In our metasurface design (Figure S1a), each unit cell consists of four nanopillars arranged in a tetramer configuration. The diagonal nanopillars share identical geometric parameters, and the anti-diagonal pair shares a different set. Neglecting inter-unit coupling, the effective Jones matrix of the tetramer unit cell is the average of the four nanopillar responses: $J_{\text{unit}} = \frac{1}{4}\sum_{i=1}^{4} J_i$. More explicitly, the unit cell Jones matrix can be written as:

$$T_{\text{LP}} = \frac{1}{2}R(\alpha_1)\begin{pmatrix} e^{i\varphi_{1,xx}} & 0 \\ 0 & e^{i\varphi_{1,yy}} \end{pmatrix}R^{-1}(\alpha_1) + \frac{1}{2}R(\alpha_2)\begin{pmatrix} e^{i\varphi_{2,xx}} & 0 \\ 0 & e^{i\varphi_{2,yy}} \end{pmatrix}R^{-1}(\alpha_2) \tag{S1}$$

Where α_1 and α_2 are the rotation angles of the diagonal and anti-diagonal nanopillars. $\varphi_{1,xx}$, $\varphi_{1,yy}$ and $\varphi_{2,xx}$, $\varphi_{2,yy}$ represent the phase delays along the x and y directions of the two pairs of nanostructures, which can be independently controlled. Here we set $\varphi_{1,xx} = \varphi_{1,yy} + \pi$, $\varphi_{2,xx} = \varphi_{2,yy} + \pi$, that is, such Jones matrix can function as half waveplate for different polarizations. Hence, after transformation from linear polarization basis to circularly polarized basis, the Jones matrix can be expressed as:

$$T_{cir} = \frac{1}{2}\begin{pmatrix} 0 & e^{i\varphi_{1,xx} - 2\alpha_1} \\ e^{i\varphi_{1,xx} + 2\alpha_1} & 0 \end{pmatrix} + \frac{1}{2}\begin{pmatrix} 0 & e^{i\varphi_{2,xx} - 2\alpha_2} \\ e^{i\varphi_{2,xx} + 2\alpha_2} & 0 \end{pmatrix} \tag{S2}$$

Hence, through the proper combination of $\varphi_{1,xx}$, $\varphi_{1,yy}$ and $\varphi_{2,xx}$, $\varphi_{2,yy}$, arbitrary complex amplitude

on the anti-diagonal channels can be achieved. Therefore, for arbitrary N dimensional state $|\Psi\rangle=\sum_{i=1}^{N}a_i|l_i,\sigma_i\rangle=\sum_j a_j|l_j,R\rangle+\sum_k a_k|l_k,L\rangle$, the desired Jones matrix can be written as:

$$T_{need}=\begin{pmatrix}0 & \sum_{k=1}^{K}a_k|l_k,L\rangle\\ \sum_{j=1}^{J}a_j|l_j,R\rangle & 0\end{pmatrix}=\begin{pmatrix}0 & a_{rl}e^{i\varphi_{\cdot}}\\ a_{lr}e^{i\varphi_{\cdot}} & 0\end{pmatrix} \tag{S3}$$

Where k and j refer to the indexes of the corresponding polarization basis for composition of the Hilbert space, with K+J=N. This corresponds to

$$\begin{cases}\varphi_{1,xx}=\dfrac{\varphi_{rl}+\varphi_{lr}+\cos^{-1}(a_{rl})+\cos^{-1}(a_{lr})}{2}\\ \varphi_{2,xx}=\dfrac{\varphi_{rl}+\varphi_{lr}-\cos^{-1}(a_{rl})-\cos^{-1}(a_{lr})}{2}\\ \alpha_1=\dfrac{-\varphi_{rl}+\varphi_{lr}-\cos^{-1}(a_{rl})+\cos^{-1}(a_{lr})}{4}\\ \alpha_2=\dfrac{-\varphi_{rl}+\varphi_{lr}+\cos^{-1}(a_{rl})-\cos^{-1}(a_{lr})}{4}\end{cases} \tag{S4}$$

Subsequently, the desired local phase profiles are mapped to specific meta-atom geometries (length and width) using the design map shown in Figure S1(c) and (d), thereby completing the metasurface which is capable of generating arbitrary N-dimensional spin–orbit states.

2. Interferometry-projection-tomograph method for arbitrary high-dimensional spin-orbit state density matrix reconstruction

We first introduce the basic principle of off-axis interference for complex field reconstruction. The optical system consists of two coherent beams: a signal beam passing through the sample and a reference beam. These two beams interfere at the detector plane, forming an interferogram from which amplitude and phase information can be extracted. Suppose the complex amplitude distributions of the signal and reference beams at the detector be:

$$E_s(x,y)=A_s(x,y)e^{i\phi_s(x,y)},\quad E_r(x,y)=A_r(x,y)e^{i\phi_r(x,y)} \tag{S5}$$

The recorded intensity on the CCD is:

$$I(x,y)=|E_s(x,y)+E_r(x,y)|^2=|E_s|^2+|E_r|^2+E_sE_r^*e^{i\Delta k\cdot r}+E_s^*E_re^{-i\Delta k\cdot r} \tag{S6}$$

Here, $\Delta\mathbf{k}=\mathbf{k}_{\text{sig}}-\mathbf{k}_{\text{ref}}$ represents the wavevector difference between the two beams, and

$\mathbf{r}(x, y)$ denotes the spatial coordinates. The cross-term encodes the interference fringes modulated by the complex amplitude of the signal field. By performing a 2D Fourier transform of $I(x, y)$, we have

$$\tilde{I}(\mathbf{k}) = \mathcal{F}\{|E_s|^2\}(\mathbf{k}) + \mathcal{F}\{|E_r|^2\}(\mathbf{k}) + \mathcal{F}\{E_s E_r^*\}(\mathbf{k} - \Delta\mathbf{k}) + \mathcal{F}\{E_s^* E_r\}(\mathbf{k} + \Delta\mathbf{k}) \quad \text{(S7)}$$

The first two terms form a dominant DC component centered at the origin, while the last two terms correspond to two symmetrically displaced sidebands originating from the cross term and its complex conjugate. The off-axis carrier $\Delta\mathbf{k}$ is chosen such that the sidebands are well separated from the DC term. We isolate one sideband using a circular band-pass mask covering the selected sideband peak; the mask radius is chosen to fully include the signal bandwidth while avoiding overlap with the DC and the conjugate sideband. The filtered sideband is then shifted to the origin and inverse Fourier transformed to recover the complex cross term $E_s E_r^*$.

$$E_s E_r^* = |E_s||E_r| e^{i(\phi_{sample} + \phi_{system} - \phi_r)} \quad \text{(S8)}$$

To remove the system background and eliminate the reference-field contribution, a background measurement is taken without the sample in advance to obtain the. $\phi_{system} - \phi_r$ Then the sample-induced complex field can be written as:

$$E_{\text{sample}}(x, y) = \frac{E_s(x, y) E_r^*(x, y)}{|E_r(x, y)| e^{i(\phi_{system} - \phi_r)}} \quad \text{(S9)}$$

The same procedure is applied to each polarization projection (*H*/*V*/*D*) to obtain the complex fields E_H, E_V, E_D, which are then used for the subsequent mode decomposition and state reconstruction. Suppose the spin–orbit state is written as:

$$|\psi\rangle = \sum_{j=1}^{N} (a_j |l_j, L\rangle + b_j |l_j, R\rangle) \quad \text{(S10)}$$

By projecting onto different polarization bases and reconstructing the corresponding complex amplitudes, we obtain the projection components as follows:

Horizontal polarization:

$$\frac{a_j + b_j}{a_1 + b_1} = \frac{\langle l_j | \langle H | \Psi \rangle}{\langle l_1 | \langle H | \Psi \rangle} = \frac{\iint E_H e^{-il_j\varphi}}{\iint E_H e^{-il_1\varphi}} = \frac{c_{H,j}}{c_{H,1}} \quad \text{(S11)}$$

Vertical polarization:

$$\frac{a_j - b_j}{a_1 - b_1} = \frac{\langle l_j|\langle V|\Psi\rangle}{\langle l_1|\langle V|\Psi\rangle} = \frac{\iint E_V e^{-il_j\varphi}}{\iint E_V e^{-il_1\varphi}} = \frac{c_{V,j}}{c_{V,1}} \tag{S12}$$

Diagonal polarization:

$$\frac{ia_j + b_j}{ia_1 + b_1} = \frac{\langle l_j|\langle D|\Psi\rangle}{\langle l_1|\langle D|\Psi\rangle} = \frac{\iint E_D e^{-il_j\varphi}}{\iint E_D e^{-il_1\varphi}} = \frac{c_{D,j}}{c_{D,1}} \tag{S13}$$

Here, E_H, E_V, E_D are measured complex amplitudes of three polarization projection from interference. These three projections provide an overdetermined linear system for solving the complex ratio vector $\mathbf{x} = [a_1/a_1, b_1/a_1, a_2/a_1, b_2/a_1, \ldots, a_N/a_1, b_N/a_1]$. Solving this system yields the spin–orbit state up to a global phase factor. The corresponding density matrix is given by: $\rho = |\psi\rangle\langle\psi|$. The fidelity between the reconstructed and theoretical states is calculated as:

$F = \left[\mathrm{Tr}\left(\sqrt{\sqrt{\rho_{\exp}}\rho_{\mathrm{th}}\sqrt{\rho_{\exp}}}\right)\right]^2$. To clarify the full data-processing procedure, the overall workflow is schematically summarized in Figure S2. This interferometry-projection-tomograph method enables efficient characterization of arbitrary high-dimensional spin–orbit states with only three interferograms.

As a representative example, we present here the complete data-processing procedure for Sample #1, following exactly the interferometry–projection–tomography method described above. According to Eq. (S10), the spin–orbit state can be expressed in the full measurement basis as:

$$\begin{aligned} |\psi\rangle = a_1|2,L\rangle + b_1|2,R\rangle + a_2|-2,L\rangle + b_2|-2,R\rangle \\ + a_3|-1,L\rangle + b_3|-1,R\rangle + a_4|1,L\rangle + b_4|1,R\rangle \end{aligned} \tag{S14}$$

For this sample, three off-axis interferograms are recorded under the horizontal, vertical, and diagonal polarization projections, respectively. For each projection, the interferogram is processed by a two-dimensional Fourier transform, sideband filtering, shifting to the origin, and inverse Fourier transform, so that the corresponding complex field distributions $\langle H|\Psi\rangle$, $\langle V|\Psi\rangle$ and $\langle D|\Psi\rangle$ are retrieved. These reconstructed complex fields are then decomposed into the OAM eigenmodes $|l = \pm 2\rangle$ and $|l = \pm 1\rangle$. From Eq. (S11)–(S13), the measured modal coefficients give the following set of relative equations:

$$\frac{a_2+b_2}{a_1+b_1}=1.12 - 0.57i\,;\frac{a_3+b_3}{a_1+b_1}=0.20 + 1.07i\,;\frac{a_4+b_4}{a_1+b_1}=0.27 + 0.96i$$
$$\frac{a_2-b_2}{a_1-b_1}=-0.86 + 0.04i;\frac{a_3-b_3}{a_1-b_1}=0.07 + 0.77i\,;\frac{a_4-b_4}{a_1-b_1}=-0.05 - 0.76i \tag{S15}$$
$$\frac{ia_2+b_2}{a_1+b_1}= -0.50 - 1.39i\,;\frac{ia_3+b_3}{a_1+b_1}=0.31 + 0.87i\,;\frac{ia_4+b_4}{a_1+b_1}=1.18 - 0.44i$$

These nine equations form an overdetermined linear system for the seven unknown relative coefficients in the vector $\mathbf{x}=[\frac{a_1}{a_1},\frac{b_1}{a_1},\frac{a_2}{a_1},\frac{b_2}{a_1},\frac{a_3}{a_1},\frac{b_3}{a_1},\frac{a_4}{a_1},\frac{b_4}{a_1}]$

By solving this system using a least-squares procedure, we obtain:

$$\begin{aligned}\mathbf{x}=[\ &1, -0.12 - 0.13i ,-0.02 - 0.35i, 0.93 - 0.28i,\\ &0.15 + 0.91i, 0.15 + 0.02i, 0.21 - 0.04i, 0.18 + 0.85i]\end{aligned} \tag{S16}$$

This solution corresponds to the experimentally reconstructed state in the full eight-dimensional measurement space. For Sample #1, however, the designed target state occupies only the four-dimensional subspace spanned by $\{|2,L\rangle,|-2,R\rangle,|-1,L\rangle,|1,R\rangle\}$. Therefore, for direct comparison with the target design, we restrict the reconstructed result to this four-dimensional subspace. The experimentally reconstructed state in the target subspace can then be written as:

$$\begin{aligned}|\psi_{1\exp}\rangle &= a_1\,|2,L\rangle + b_2\,|-2,L\rangle + a_3\,|-1,L\rangle + b_4\,|1,R\rangle\\ &=|2,L\rangle+(0.93-0.28i)\,|-2,R\rangle+(0.15+0.91i)\,|-1,L\rangle+(0.18+0.85i)\,|1,R\rangle\end{aligned} \tag{S17}$$

After normalization, it becomes:

$$\begin{aligned}|\psi_{1\exp}\rangle &= 0.53\,|2,L\rangle+(0.50-0.15i)\,|-2,R\rangle\\ &+(0.08+0.48i)\,|-1,L\rangle+(0.09+0.45i)\,|1,R\rangle\end{aligned} \tag{S18}$$

The corresponding experimental density matrix is reconstructed as:

$$\begin{aligned}\rho_{1\exp} &= \left|\psi_{1\exp}\right\rangle\left\langle\psi_{1\exp}\right|\\ &= \begin{bmatrix} 0.28 + 0.00i & 0.26 - 0.08i & 0.04 + 0.26i & 0.05 + 0.24i \\ 0.26 + 0.08i & 0.27 + 0.00i & -0.03 + 0.25i & -0.02 + 0.24i \\ 0.04 - 0.26i & -0.03 - 0.25i & 0.24 + 0.00i & 0.22 - 0.01i \\ 0.05 - 0.24i & -0.02 - 0.24i & 0.22 + 0.01i & 0.21 + 0.00i \end{bmatrix}\end{aligned} \tag{S19}$$

For Sample #1, the theoretical density matrix is:

$$\rho_{1th} = |\psi_{1th}\rangle\langle\psi_{1th}| = \begin{bmatrix} 0.25 & 0.25 & 0.25i & 0.25i \\ 0.25 & 0.25 & 0.25i & 0.25i \\ -0.25i & -0.25i & 0.25 & 0.25 \\ -0.25i & -0.24i & 0.25 & 0.25 \end{bmatrix} \tag{S20}$$

The fidelity between the reconstructed and theoretical density matrices is then calculated as

$$F_1 = \left[\mathrm{Tr}\left(\sqrt{\sqrt{\rho_{1\exp}}\,\rho_{1\mathrm{th}}\sqrt{\rho_{1\exp}}}\right)\right]^2 = 0.985$$

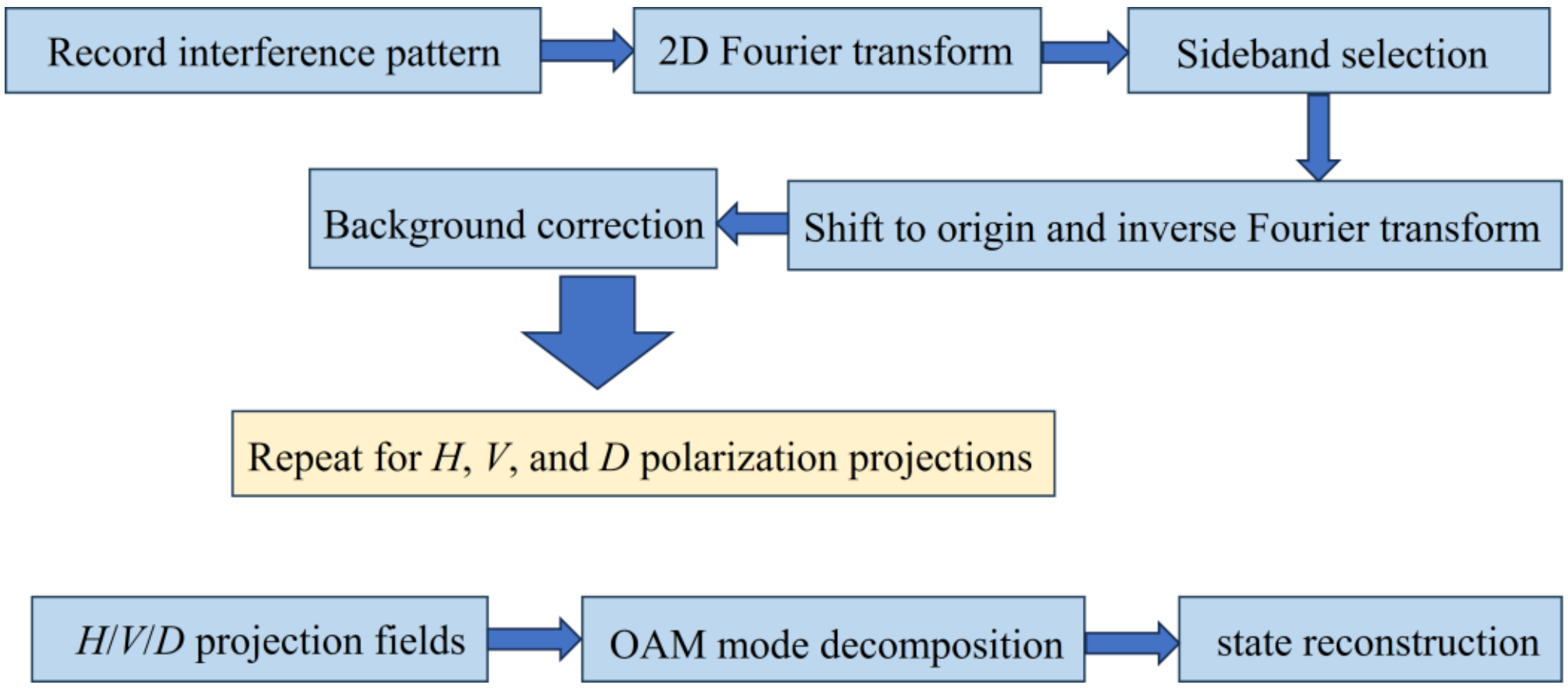

Figure S2. Schematic workflow of the Interferometry-projection-tomograph method.

3. Experimental setup for the interferometric reconstruction of density matrix

Figure S3 illustrates the experimental setup used for the complex amplitude measurement and density matrix reconstruction. A linearly polarized laser beam is split into a signal and a reference arm using a non-polarizing beam splitter. The signal beam passes through a polarizer and the designed metasurface sample, and is then relayed to a CCD camera by a 4f imaging system (focal lengths of 100 mm and 200 mm), with magnification of ×2. The reference beam is reflected by a slightly tilted mirror to introduce a small off-axis angle, enabling off-axis interference at the image plane. A half-wave plate is used to change the polarization of reference beam. The interference fringes recorded at the CCD allow retrieval of the full complex amplitude of the output field. By changing the output polarization projection through linear polarizer before CCD, density matrices of spin–orbit states can be reconstructed.

Figures S4 and S5 present the experimentally measured and numerically simulated complex amplitude distributions for Samples #1–4 under three representative polarization projections: |*H*>, |*V*>, and |*D*> linear polarization projection. The experimental results, obtained using the setup

described in Figure S3, show a high level of agreement with the theoretical predictions. Both the amplitude and phase exhibit consistent variation along the azimuthal direction, validating the designed spin–orbit state structure.

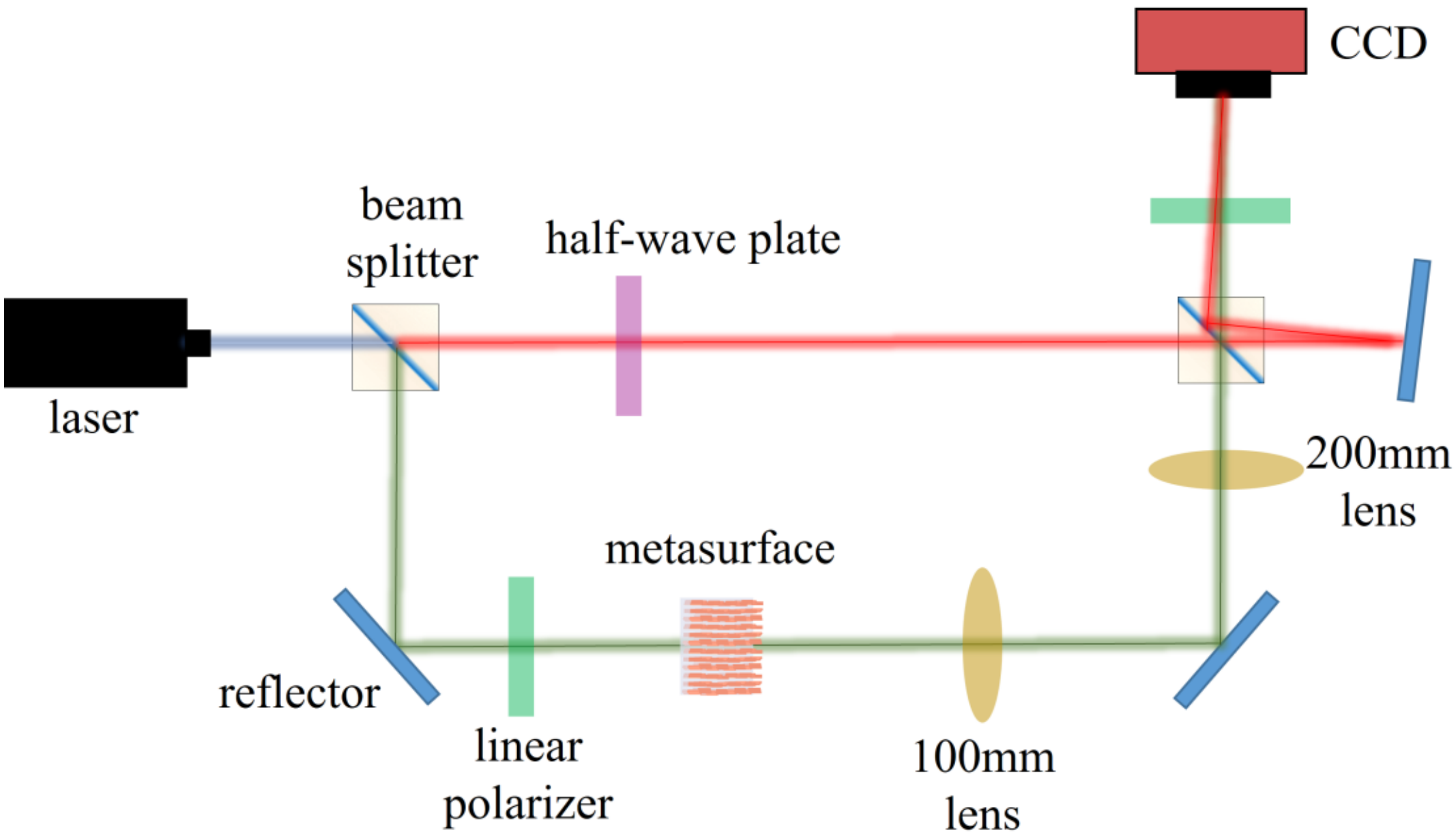

Figure S3. Schematic diagram of density matrix reconstruction experiment

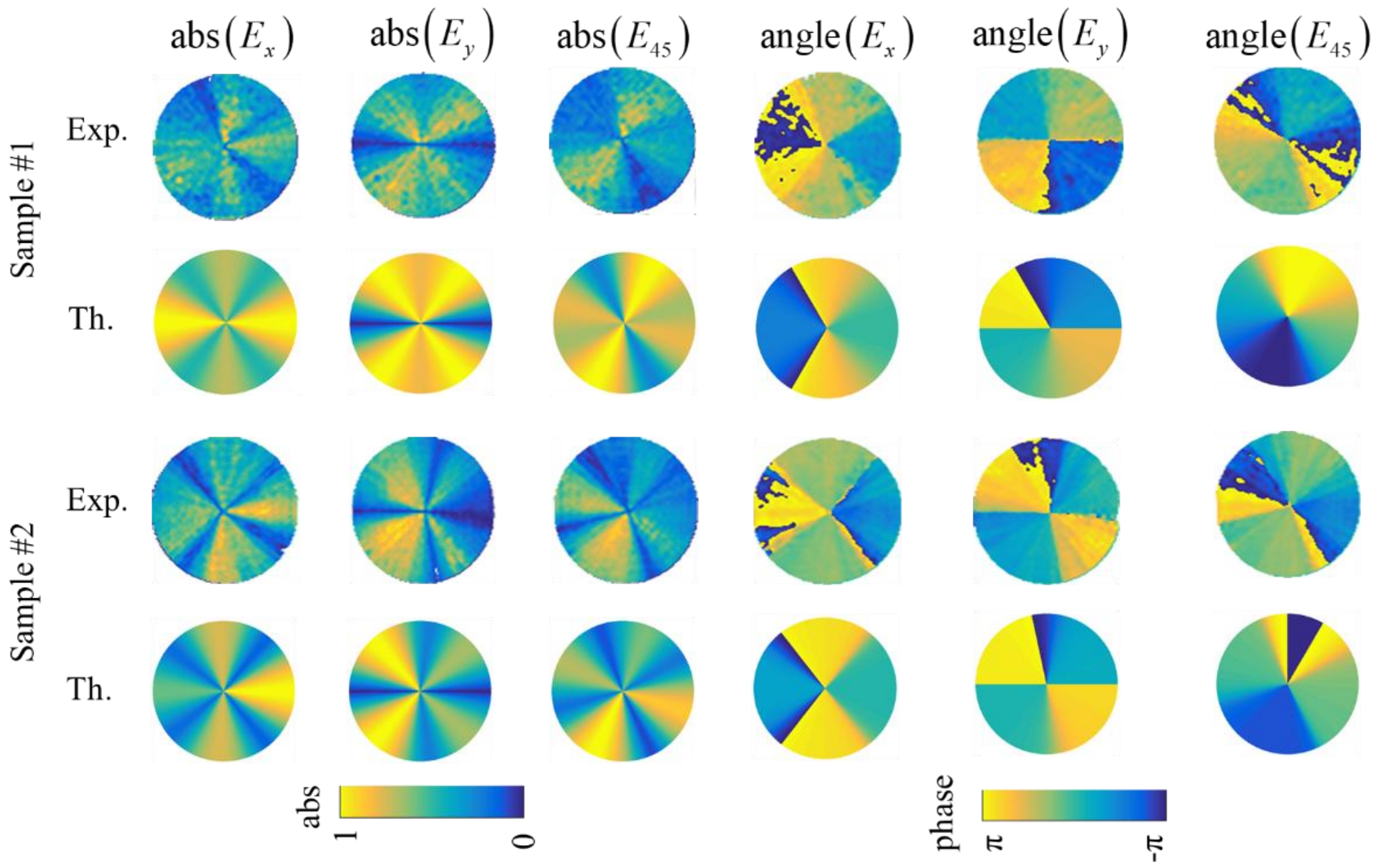

Figure S4. Experimental and simulated complex amplitudes of Samples #1 and #2 under three polarization projections.

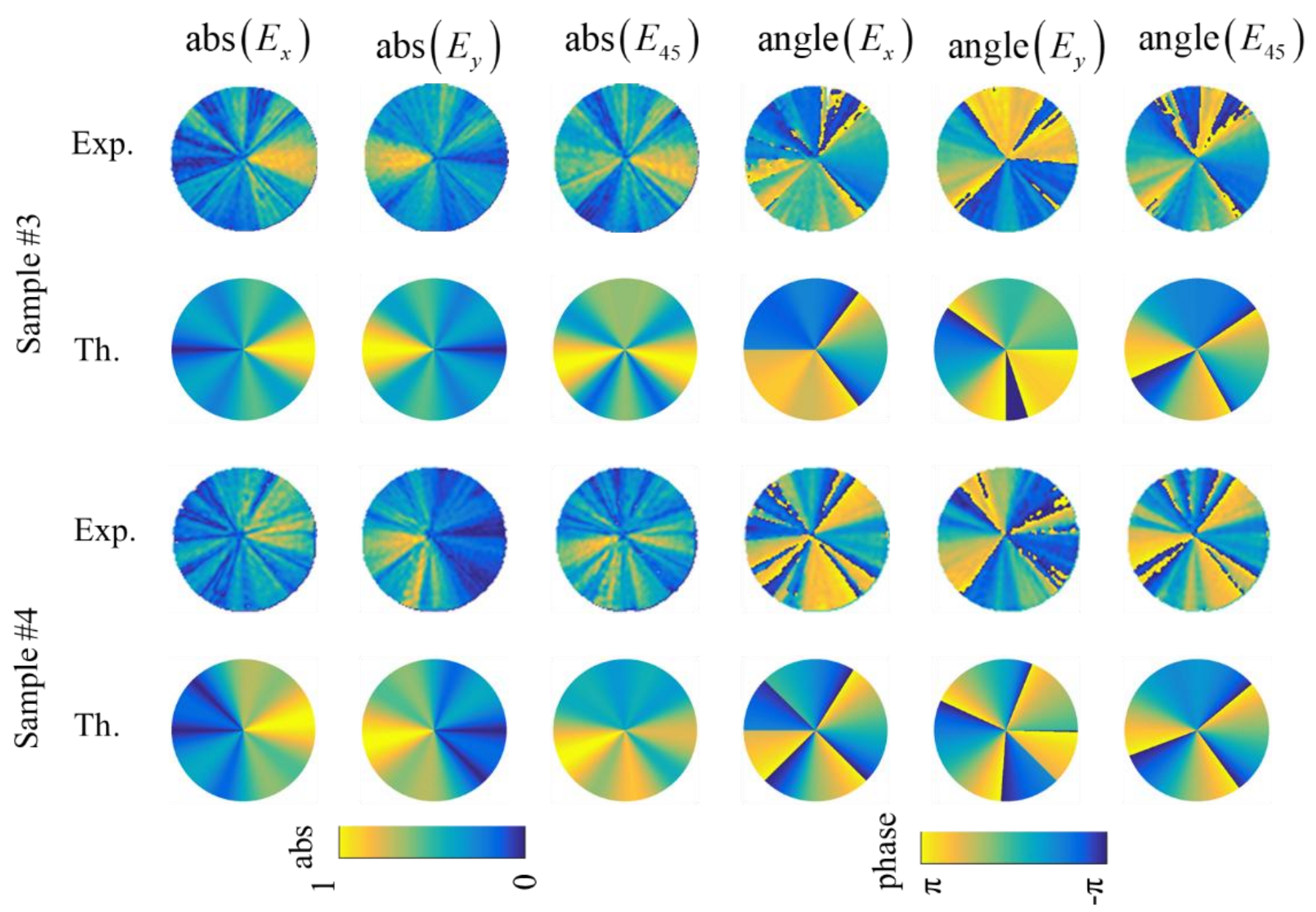

Figure S5. Experimental and simulated complex amplitudes of Sample #3 and #4 under three polarization projections.

4. Experimental setup for far-field intensity imaging and polarization projection

Figure S6 shows the experimental setup used for far-field intensity imaging and verification of the spin–orbit parity order. A linearly polarized tunable femtosecond laser beam is normally incident onto the metasurface sample, which is mounted on a rotation stage. The transmitted beam is projected onto selected polarization bases by tuning the quarter-wave plate and polarizer behind the metasurface. The far-field intensity distributions are directly recorded by a CCD camera placed at the back focal plane of the imaging lens. This setup allows direct visualization of the spatial structure of spin–orbit states and enables the analysis of their spatial symmetry. In the experiment, the incident wavelength is 900 nm for Sample #1, 830 nm for Sample #2, and 840 nm for both Sample #3 and Sample #4.

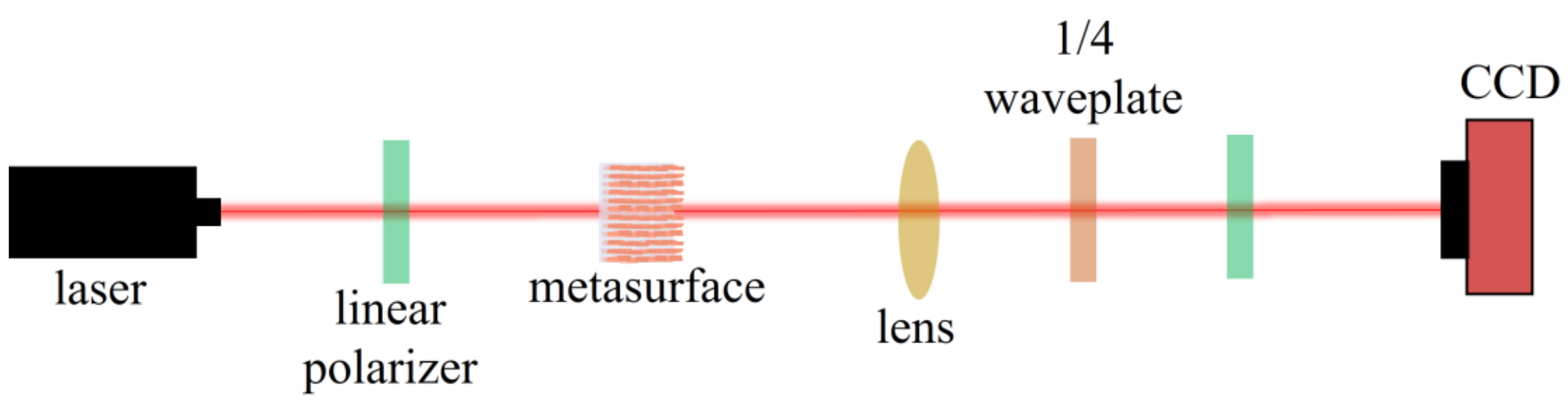

Figure S6 The experimental setup for verification of spin-orbit parity order in high-dimensional

spin-orbit Hilbert space.

5. Removal of crosstalk terms

Due to fabrication imperfections, wavelength shifts are observed for each sample. These imperfections can also introduce undesired crosstalk components during the generation of high-dimensional spin–orbit states. Fortunately, by applying the high-dimensional spin–orbit state characterization method developed in the main text, we are able to eliminate crosstalk. This not only provides strong validation for the spin–orbit parity order we proposed, but also highlights the reliability and practical applicability of our reconstruction technique.

In our experiment, the projections of the high-dimensional spin–orbit field onto horizontal (|*H*>) and vertical (|*V*>) polarization bases are measured. However, the complex relative amplitude factor t between these two projections remains unknown. Because for the co-polarization input/output channel, there will be residual light passing through the metasurface. Additionally, although the complex weights $\mathbf{x}=[a_1/a_1, b_1/a_1, a_2/a_1, b_2/a_1, \ldots, a_N/a_1, b_N/a_1]$ can be computed from Section 2, the reference coefficient a_1 is initially undetermined. Without loss of generality, we choose the x-polarized projection (|*H*>) as the reference basis for normalization.

$$\vec{\mathrm{E}}_{high-\dim} = \left(E_{x,measure}, tE_{y,measure}\right)^T \quad \text{(S21)}$$

Once the value of t is determined, the complete polarization amplitude and phase of the high-dimensional field can be reconstructed, hence the far-field diffraction pattern can be directly computed. By substituting Eq. (S11), we have

$$\iint E_{x,measure} e^{-il_j\varphi} = c_{H,j} = a_j + b_j = a_1\left(x_{2j-1} + x_{2j}\right) \quad \text{(S22)}$$

From this formula, we can compute a_1 for each mode index j. Averaging the results over all j helps suppress random noise and enhance robustness. Similarly, the expression for the y-polarized projection (|*V*>) is given by:

$$\iint tE_{y,measure} e^{-il_j\varphi} = tc_{V,j} = a_j - b_j \quad \text{(S23)}$$

Averaging the calculated t values across all j yields the global complex coefficient between the two orthogonal projections. With this information, any unwanted spin–orbit components (e.g, m-th mode $|l_m, L\rangle$ and n-th mode $|l_n, R\rangle$) can be numerically removed using:

$$E_{x,correct}=E_{x,measure}-\frac{a_m\left|l_m,L\right\rangle}{\sqrt{2}\left\langle l_m,L\middle|l_m,L\right\rangle}-\frac{b_n\left|l_n,R\right\rangle}{\sqrt{2}\left\langle l_n,R\middle|l_n,R\right\rangle} \tag{S24}$$

$$E_{y,correct}=tE_{y,measure}-\frac{ia_m\left|l_m,L\right\rangle}{\sqrt{2}\left\langle l_m,L\middle|l_m,L\right\rangle}+\frac{ib_n\left|l_n,R\right\rangle}{\sqrt{2}\left\langle l_n,R\middle|l_n,R\right\rangle} \tag{S25}$$

For any desired polarization projection $\vec{\mathrm{p}}=(a,b)^T$, we have:

$$E_{p,remove}=a^*E_{x,correct}+b^*E_{y,correct} \tag{S26}$$

Then, the corresponding far-field intensity pattern free from crosstalk can be computed through numerical diffraction propagation. In the case of P=1 (Sample #3 and #4), we remove the crosstalk $\left|4,R\right\rangle$, $\left|3,L\right\rangle$, $\left|2,R\right\rangle$, $\left|-1,L\right\rangle$, $\left|5,R\right\rangle$ and $\left|5,L\right\rangle$ and the final experimental results are shown in Figure 4c, with good agreement with theoretical design.

6. The relationship between spin-orbit order parameter and centrosymmetry of linear polarization projection

We consider an arbitrary high-dimensional spin–orbit state:

$$\left|\Psi\right\rangle=\sum_{\mathrm{i}=1}^{N}a_i\left|l_i,\sigma_i\right\rangle \tag{S27}$$

Any linearly polarized state and its orthogonal counterpart can be expressed in the circular polarization basis as:

$$\begin{aligned}\left|\psi_p\right\rangle&=e^{-i\frac{\varphi_p}{2}}\left|R\right\rangle+e^{i\frac{\varphi_p}{2}}\left|L\right\rangle\\ \left|\psi_{p\perp}\right\rangle&=-e^{-i\frac{\varphi_p}{2}}\left|R\right\rangle+e^{i\frac{\varphi_p}{2}}\left|L\right\rangle\end{aligned} \tag{S28}$$

Here, we neglect the global phase of the state. The projection of the high-dimensional spin–orbit state onto this pair of linear polarization states yields:

$$E_p=\left\langle\Psi_p\middle|\Psi\right\rangle=\sum_{i=1}^{N}e^{-i\frac{\varphi_p}{2}\sigma_i}a_i\left|l_i\right\rangle \tag{S29}$$

$$E_{p\perp}=\left\langle\Psi_{p\perp}\middle|\Psi\right\rangle=\sum_{i=1}^{N}e^{-i(\frac{\varphi_p}{2}\sigma_i+\frac{\sigma_i-1}{2}\pi)}a_i\left|l_i\right\rangle \tag{S30}$$

Where $\sigma_i=1$ represent LCP, $\sigma_i=-1$ represent RCP. Rotate E_p by 180°, yields:

$$E_{p\_rot}=\sum_{i=1}^{N}e^{-i\frac{\varphi_p}{2}\sigma_i}e^{i\pi l_i}a_i\left|l_i\right\rangle \tag{S31}$$

Assuming the state $|l_i\rangle$ is normalized, i.e., $\langle l_i | l_i\rangle = 1$, we define the spin–orbit parity order parameter as $P = \langle E_{p\perp} | E_{p\_rot}\rangle$. We have:

$$P = \sum_{i=1}^{N} |a_i|^2 e^{i\frac{\sigma_i - 1}{2}\pi} e^{il_i\pi} = \sum_{i=1}^{N} |a_i|^2 \sigma_i p(l_i) \tag{S32}$$

where the parity function $p(\cdot)$ takes the value +1 for even indices and −1 for odd indices. When P=1, the two projection vectors are identical, resulting in linear-polarization projection amplitudes and phases that are mutually centrosymmetric. For P=−1, the vectors are antiparallel; in this case, the far-field intensity distribution under linear-polarization projection remains centrosymmetric, while the phase exhibits a π shift. For $|P|<1$, the two vectors form an angle with each other, and the central symmetry in the projection images is broken. This establishes a direct link between the spin–orbit parity order and the presence of central symmetry distribution under linear polarization projections.

7. Analysis of high-dimensional spin-orbit states transformation induced by higher-order geometric phase

To simply the analysis, we first consider a two-dimensional subsystem: the incident light is $|0, R\rangle$ and the outgoing light is $|l, L\rangle$. Hence the Jones matrix under the circular polarization basis can be written as $T(r,\varphi) = \begin{pmatrix} 0 & Ae^{il\varphi} \\ 0 & 0 \end{pmatrix}$. (r,φ) represent the polar coordinate. A counterclockwise rotation of the metasurface corresponds to a rotation of the path on the mixed-order Poincaré sphere, which leads to the acquisition of a higher-order geometric phase $\gamma = (l + 2\sigma)\varphi_r$. This phase accumulation can also be directly derived via a metric analysis. Specifically, when the metasurface rotates φ_0 counterclockwise, the corresponding nanopillar (r,φ) originates from $(r,\varphi - \varphi_0)$ before rotation. As the metasurface rotates, the local azimuthal angle of the nanopillar also rotates relative to the global coordinate system, giving rise to an additional geometric phase of $2\varphi_0$ in the circular polarization conversion channel. Therefore, at (r,φ), the Jones matrix after rotation should be written as:

$$T(r,\varphi)=R(\varphi_0)\begin{pmatrix}0 & Ae^{il(\varphi-\varphi_0)}\\ 0 & 0\end{pmatrix}R^{-1}(\varphi_0)$$
$$=\begin{pmatrix}e^{-i\varphi_0} & 0\\ 0 & e^{i\varphi_0}\end{pmatrix}\begin{pmatrix}0 & Ae^{il(\varphi-\varphi_0)}\\ 0 & 0\end{pmatrix}\begin{pmatrix}e^{i\varphi_0} & 0\\ 0 & e^{-i\varphi_0}\end{pmatrix}=\begin{pmatrix}0 & Ae^{il(\varphi-\varphi_0)-i2\varphi_0}\\ 0 & 0\end{pmatrix} \tag{S33}$$

$R(\varphi_0)$ is rotation metric in the circular polarization basis,

$$R(\varphi_0)=\begin{pmatrix}1 & -i\\ 1 & i\end{pmatrix}\begin{pmatrix}\cos\varphi_0 & -\sin\varphi_0\\ \sin\varphi_0 & \cos\varphi_0\end{pmatrix}\begin{pmatrix}i & i\\ -1 & 1\end{pmatrix}=\begin{pmatrix}e^{-i\varphi_0} & 0\\ 0 & e^{i\varphi_0}\end{pmatrix} \tag{S34}$$

Therefore, $E_{out}=T(r,\varphi)*E_{in}=Ae^{-i(l+2)\varphi_0}e^{il\varphi}\begin{pmatrix}1\\ 0\end{pmatrix}=e^{-i(l+2)\varphi_0}\left|l,L\right\rangle$. Due to the rotation of metasurface, outgoing state $\left|l,L\right\rangle$ acquire the geometric phase $-\gamma=-(l+2)\theta_0$ .The same derivation applies for arbitrary incident eigenstate $\left|l,\sigma\right\rangle$. Therefore, whenever the metasurface rotates, each eigenstate acquires a geometric phase $-\gamma=-(l+2\sigma)\varphi_0$.

Furthermore, we provide the general form of such high-dimensional spin-orbit states by considering different eigenstates basis as follows:

$$\left|\psi\right\rangle=\cos\left(\frac{\theta_3}{2}\right)\cos\left(\frac{\theta_1}{2}\right)e^{-i\frac{\varphi_3+\varphi_1}{2}}\left|l_1,\sigma_1\right\rangle+\cos\left(\frac{\theta_3}{2}\right)\sin\left(\frac{\theta_1}{2}\right)e^{-i\frac{\varphi_3-\varphi_1}{2}}\left|l_2,\sigma_2\right\rangle$$
$$+\sin\left(\frac{\theta_3}{2}\right)\cos\left(\frac{\theta_2}{2}\right)e^{i\frac{\varphi_3-\varphi_2}{2}}\left|l_3,\sigma_3\right\rangle+\sin\left(\frac{\theta_3}{2}\right)\sin\left(\frac{\theta_2}{2}\right)e^{i\frac{\varphi_3+\varphi_2}{2}}\left|l_4,\sigma_4\right\rangle \tag{S35}$$

After the metasurface rotates φ_r angle, the state can be expressed as:

$$\left|\psi_{\varphi_r}\right\rangle = \cos\left(\frac{\theta_3}{2}\right)\cos\left(\frac{\theta_1}{2}\right)e^{-i\frac{\varphi_3+\varphi_1+2(l_1+2\sigma_1)\varphi_r}{2}}\left|l_1,\sigma_1\right\rangle$$
$$+\cos\left(\frac{\theta_3}{2}\right)\sin\left(\frac{\theta_1}{2}\right)e^{-i\frac{\varphi_3-\varphi_1+2(l_2+2\sigma_2)\varphi_r}{2}}\left|l_2,\sigma_2\right\rangle$$
$$+\sin\left(\frac{\theta_3}{2}\right)\cos\left(\frac{\theta_2}{2}\right)e^{i\frac{\varphi_3-\varphi_2-2(l_3+2\sigma_3)\varphi_r}{2}}\left|l_3,\sigma_3\right\rangle$$
$$+\sin\left(\frac{\theta_3}{2}\right)\sin\left(\frac{\theta_2}{2}\right)e^{i\frac{\varphi_3+\varphi_2-2(l_4+2\sigma_4)\varphi_r}{2}}\left|l_4,\sigma_4\right\rangle$$
$$= e^{\varphi_{global}}\left\{\begin{array}{l}\cos\left(\frac{\theta_3}{2}\right)e^{-i\frac{\varphi_3+\left(\frac{l_1+l_2-l_3-l_4}{2}+\sigma_1+\sigma_2-\sigma_3-\sigma_4\right)\varphi_r}{2}} \\ \left[\cos\left(\frac{\theta_1}{2}\right)e^{-i\frac{\varphi_1+(l_1-l_2+2\sigma_1-2\sigma_2)\varphi_r}{2}}\left|l_1,\sigma_1\right\rangle+\sin\left(\frac{\theta_1}{2}\right)e^{i\frac{\varphi_1+(l_1-l_2+2\sigma_1-2\sigma_2)\varphi_r}{2}}\left|l_2,\sigma_2\right\rangle\right] \\ +\sin\left(\frac{\theta_3}{2}\right)e^{i\frac{\varphi_3+\left(\frac{l_1+l_2-l_3-l_4}{2}+\sigma_1+\sigma_2-\sigma_3-\sigma_4\right)\varphi_r}{2}} \\ \left[\cos\left(\frac{\theta_2}{2}\right)e^{-i\frac{\varphi_2+(l_3-l_4+2\sigma_3-2\sigma_4)\varphi_r}{2}}\left|l_3,\sigma_3\right\rangle+\sin\left(\frac{\theta_2}{2}\right)e^{i\frac{\varphi_2+(l_3-l_4+2\sigma_3-2\sigma_4)\varphi_r}{2}}\left|l_4,\sigma_4\right\rangle\right]\end{array}\right\} \quad \text{(S36)}$$

Where $\varphi_{global} = -\left(\frac{l_1+l_2+l_3+l_4+2\sigma_1+2\sigma_2+2\sigma_3+2\sigma_4}{4}\right)\varphi_r - \frac{w_1+w_2}{2}\pi - w_3\pi$,

$w_1 = \left[\left|\frac{(l_1-l_2+2\sigma_1-2\sigma_2)\varphi_r}{2\pi}\right|\right]$, $w_2 = \left[\left|\frac{(l_3-l_4+2\sigma_3-2\sigma_4)\varphi_r}{2\pi}\right|\right]$,

$w_3 = \left[\left|\left(\frac{l_1+l_2-l_3-l_4}{2}+\sigma_1+\sigma_2-\sigma_3-\sigma_4\right)\varphi_r+(w_1-w_2)\pi\right|\right]$. $[x]$ represents the biggest integer that smaller than x. w_1, w_2 and w_3 denote the number of full 2π rotations around HOPS I, II and III, respectively. These three terms originate from the double covering of the SU(2) to SO(3) mapping. So, one point $[\varphi_1,\theta_1,\varphi_2,\theta_2,\varphi_3,\theta_3]$ on four-dimensional poincaré hypersphere will be transformed into:

$$\begin{bmatrix}\varphi_1+(l_1-l_2+2\sigma_1-2\sigma_2)\varphi_r,\theta_1,\varphi_2+(l_3-l_4+2\sigma_3-2\sigma_4)\varphi_r,\theta_2, \\ \varphi_3+\left(\frac{l_1+l_2-l_3-l_4}{2}+\sigma_1+\sigma_2-\sigma_3-\sigma_4\right)\varphi_r+(w_1-w_2)\pi,\theta_3\end{bmatrix} \quad \text{(S37)}$$

And such global phase of φ_{global} refers to higher order geometric phase in Hilbert space.

8 Effect of changing incident polarization

In a general case, the incident light can be of arbitrary polarization, which is characterized geometrically through polarization Poincaré sphere.

$$\left|\psi\right\rangle_{in} = \cos(\frac{\theta_p}{2})e^{-i\frac{\varphi_p}{2}}\left|0,R\right\rangle + \sin(\frac{\theta_p}{2})e^{i\frac{\varphi_p}{2}}\left|0,L\right\rangle \tag{S38}$$

$$\begin{aligned}
\left|\psi\right\rangle_{\text{out}} &= J\left|\psi\right\rangle_{in} \\
&= \frac{1}{2}\cos\left(\frac{\theta_3}{2}\right)\cos\left(\frac{\theta_1}{2}\right)\left[\cos(\frac{\theta_p}{2})(\sigma_1+1)-\sin(\frac{\theta_p}{2})(\sigma_1-1)\right]e^{-i\frac{\varphi_3+\varphi_1+\sigma_1\varphi_p}{2}}\left|l_1,\sigma_1\right\rangle \\
&+\frac{1}{2}\cos\left(\frac{\theta_3}{2}\right)\sin\left(\frac{\theta_1}{2}\right)\left[\cos(\frac{\theta_p}{2})(\sigma_2+1)-\sin(\frac{\theta_p}{2})(\sigma_2-1)\right]e^{-i\frac{\varphi_3-\varphi_1+\sigma_2\varphi_p}{2}}\left|l_2,\sigma_2\right\rangle \\
&+\frac{1}{2}\sin\left(\frac{\theta_3}{2}\right)\cos\left(\frac{\theta_2}{2}\right)\left[\cos(\frac{\theta_p}{2})(\sigma_3+1)-\sin(\frac{\theta_p}{2})(\sigma_3-1)\right]e^{i\frac{\varphi_3-\varphi_2-\sigma_3\varphi_p}{2}}\left|l_3,\sigma_3\right\rangle \\
&+\frac{1}{2}\sin\left(\frac{\theta_3}{2}\right)\sin\left(\frac{\theta_2}{2}\right)\left[\cos(\frac{\theta_p}{2})(\sigma_4+1)-\sin(\frac{\theta_p}{2})(\sigma_4-1)\right]e^{i\frac{\varphi_3+\varphi_2-\sigma_4\varphi_p}{2}}\left|l_4,\sigma_4\right\rangle
\end{aligned} \tag{S39}$$

Where J is Jones matrix of formula (3) in the main text. Rewrite formula S30 in the standard form:

$$\begin{aligned}
\left|\psi\right\rangle_{\text{out}} &= A\cos\left(\frac{\theta_3'}{2}\right)e^{-i\frac{\varphi_3'}{2}}\left[\cos(\frac{\theta_1'}{2})e^{-i\frac{\varphi_1'}{2}}\left|l_1,\sigma_1\right\rangle + \sin\left(\frac{\theta_1'}{2}\right)e^{i\frac{\varphi_1'}{2}}\left|l_2,\sigma_2\right\rangle\right] \\
&+A\sin\left(\frac{\theta_3'}{2}\right)e^{i\frac{\varphi_3'}{2}}\left[\cos\left(\frac{\theta_2'}{2}\right)e^{-i\frac{\varphi_2'}{2}}\left|l_3,\sigma_3\right\rangle + \sin\left(\frac{\theta_2'}{2}\right)e^{i\frac{\varphi_2'}{2}}\left|l_4,\sigma_4\right\rangle\right]
\end{aligned} \tag{S40}$$

$$\begin{cases}
\varphi_1' = \varphi_1 + \dfrac{(\sigma_1-\sigma_2)}{2}\varphi_p \\
\theta_1' = 2\arctan\left[\tan(\dfrac{\theta_1}{2})\dfrac{A_2}{A_1}\right] \\
\varphi_2' = \varphi_2 + \dfrac{(\sigma_3-\sigma_4)}{2}\varphi_p \\
\theta_2' = 2\arctan\left[\tan(\dfrac{\theta_2}{2})\dfrac{A_4}{A_3}\right] \\
\varphi_3' = \varphi_3 + \dfrac{(\sigma_1+\sigma_2)-(\sigma_3+\sigma_4)}{4}\varphi_p \\
\theta_3' = 2\arctan\left[\tan(\dfrac{\theta_3}{2})\sqrt{\dfrac{\cos^2(\frac{\theta_2}{2})A_3^2+\sin^2(\frac{\theta_2}{2})A_4^2}{\cos^2(\frac{\theta_1}{2})A_1^2+\sin^2(\frac{\theta_1}{2})A_2^2}}\right]
\end{cases}$$
$$A_i = \frac{1}{2}\left[\cos(\frac{\theta_p}{2})(\sigma_i+1)-\sin(\frac{\theta_p}{2})(\sigma_i-1)\right], i=1:4 \tag{S41}$$

It can be seen that when the input polarization state is varied, the azimuthal angle on at least one of the Poincaré spheres remains unchanged**.** This is in stark contrast to the evolution induced by rotating the metasurface, which leads to significant azimuthal variation on all HOPS.

Figure S7 illustrates the parameters of the generated high-dimensional spin–orbit states on the

nested Poincaré hypersphere under various incident polarization states, as well as the corresponding far-field simulation results projected onto x and y polarization.

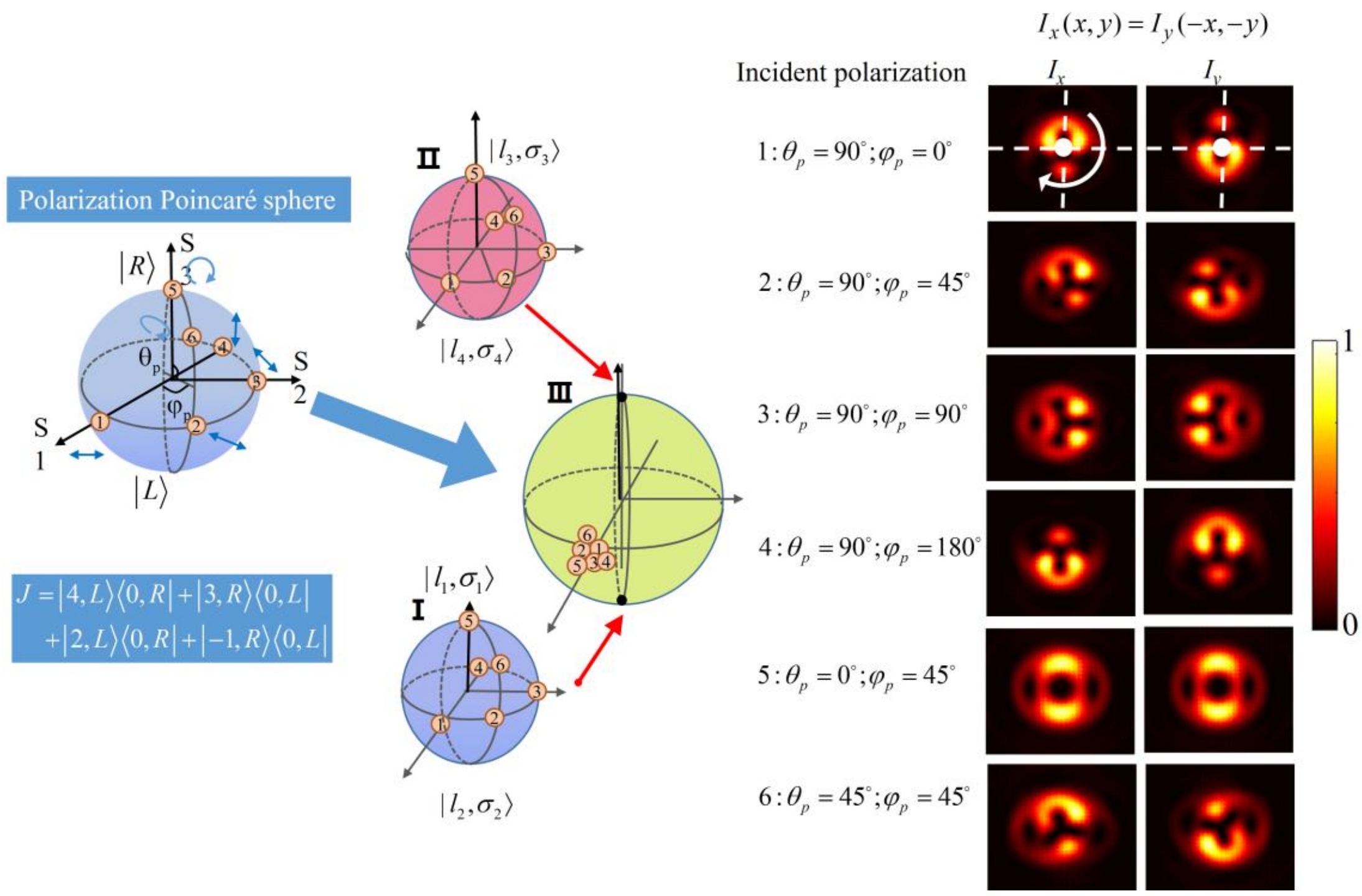

Figure S7 Far field results of sample # 3 under changing incident polarization state

9. Scalability to higher dimension

The Jones-matrix-based framework used in this work is not restricted to the four-dimensional case. In principle, higher-dimensional spin–orbit states can be generated by incorporating additional orthogonal spin–orbit eigen-components with prescribed complex weights into the target Jones matrix. To illustrate this scalability, we numerically designed a representative eight-dimensional spin–orbit state:

$$|\Psi\rangle = \frac{1}{2\sqrt{2}}(|4,L\rangle + |3,L\rangle + |2,L\rangle + |-1,L\rangle + |4,R\rangle + |3,R\rangle + |2,R\rangle + |-1,R\rangle) \tag{S42}$$

Figure S9a presents the theoretical far-field intensity under x-polarized illumination and x-polarized projection, and Figure S9b shows the corresponding calculated result after mapping the ideal continuous design onto a discrete practical meta-atom library. The good agreement between the two is further supported by the density matrix shown in Figure S10, where the calculated density matrix in Figure S10b closely matches the theoretical target in Figure S10a. The corresponding fidelity reaches 99.9%, confirming that the present Jones-matrix-based design strategy can be extended

beyond the four-dimensional Hilbert space demonstrated in the main text.

To evaluate the practical scalability, we further introduced fabrication-related perturbations into the metasurface design. Specifically, for each pixel, the length and width of the meta-atom are independently perturbed by random values chosen with equal probability from{-5nm，0，+5nm} and{-10nm，-5nm，0，+5nm，+10nm}.When increasing perturbation level, the generated far-field electromagnetic field shows increasing deviation from the target state, as shown in Figure S9c, d. The similar trend is observed in the reconstructed density matrices in Figure S10c, d. From the density matrices, the real part progressively departs from the theoretical distribution, while non-ideal imaginary components emerge, indicating increased inter-mode crosstalk. Accordingly, the state fidelity decreases to 99.5% for the ±5 nm perturbation case, and 98.2% for the ±10 nm perturbation case. Nevertheless, the fidelity remains high in both cases, indicating that the proposed design retains good robustness against moderate fabrication perturbations.

In summary, as the target Hilbert-space dimensionality increases, a larger number of spin–orbit eigen-components must be simultaneously controlled with accurate relative amplitudes and phases. This places stringent requirements on the match between the fabricated meta-atom response and the ideal design, making higher-dimensional state generation more sensitive to fabrication deviations and near-field coupling between meta-atoms. Such limitations may be alleviated in several ways. For example, one may employ meta-atoms with improved fabrication tolerance. In addition, hybrid anapole meta-atoms may help suppress near-field interactions. More generally, inverse design methods that jointly incorporate fabrication uncertainty, near-field coupling, and target state fidelity could provide a practical route toward more robust high-dimensional spin-orbit state design.

On the characterization side, higher-dimensional states generally involve finer spatial features and broader spatial frequency, which impose stricter requirements on the sampling bandwidth of the off-axis interferometric system. In practice, to ensure reliable frequency separation in the Fourier plane, the sensor sampling bandwidth should be at least four times larger than the object bandwidth.

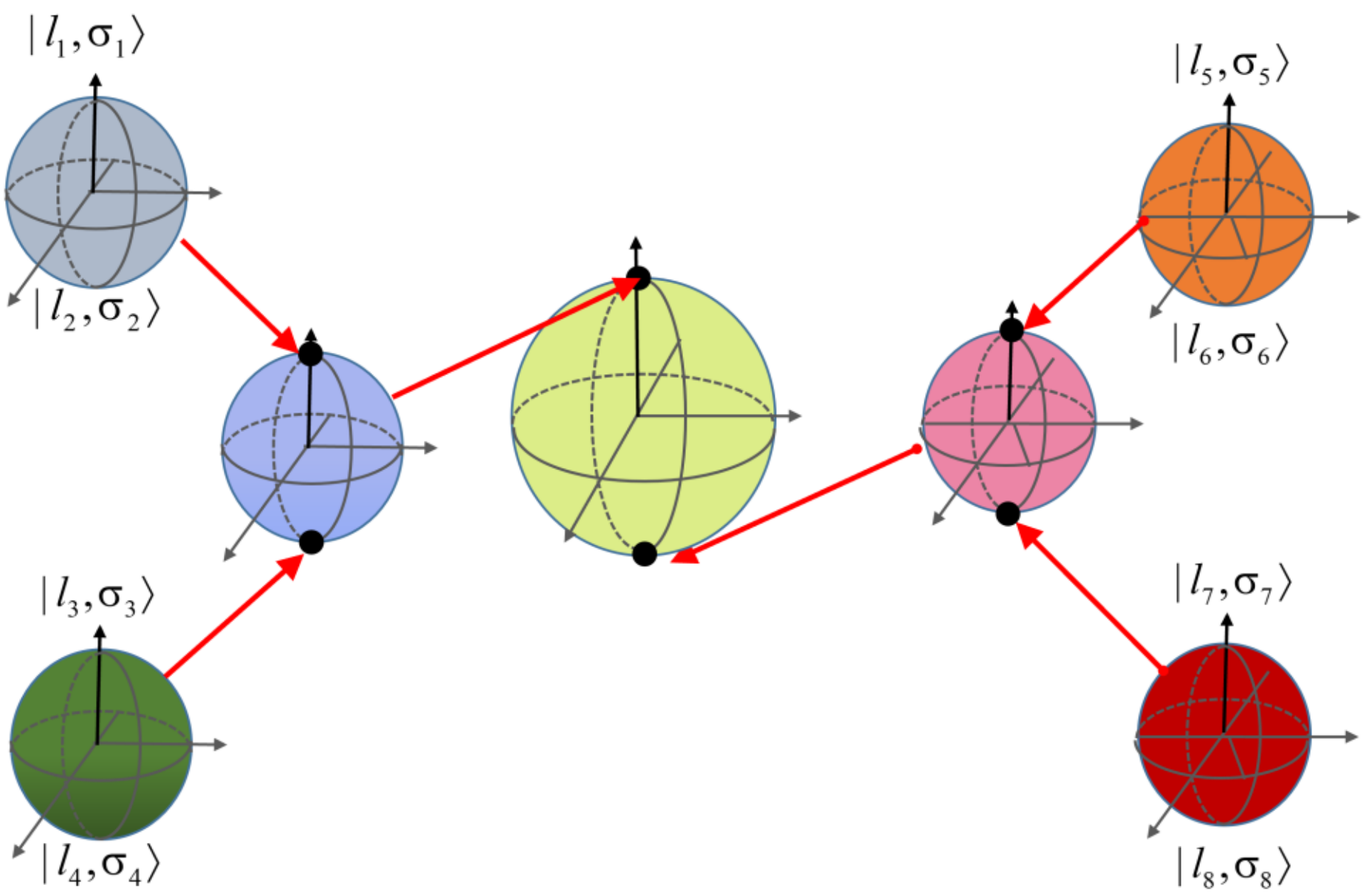

Figure S8 Poincaré hypersphere representation of the eight-dimensional spin-orbit Hilbert space.

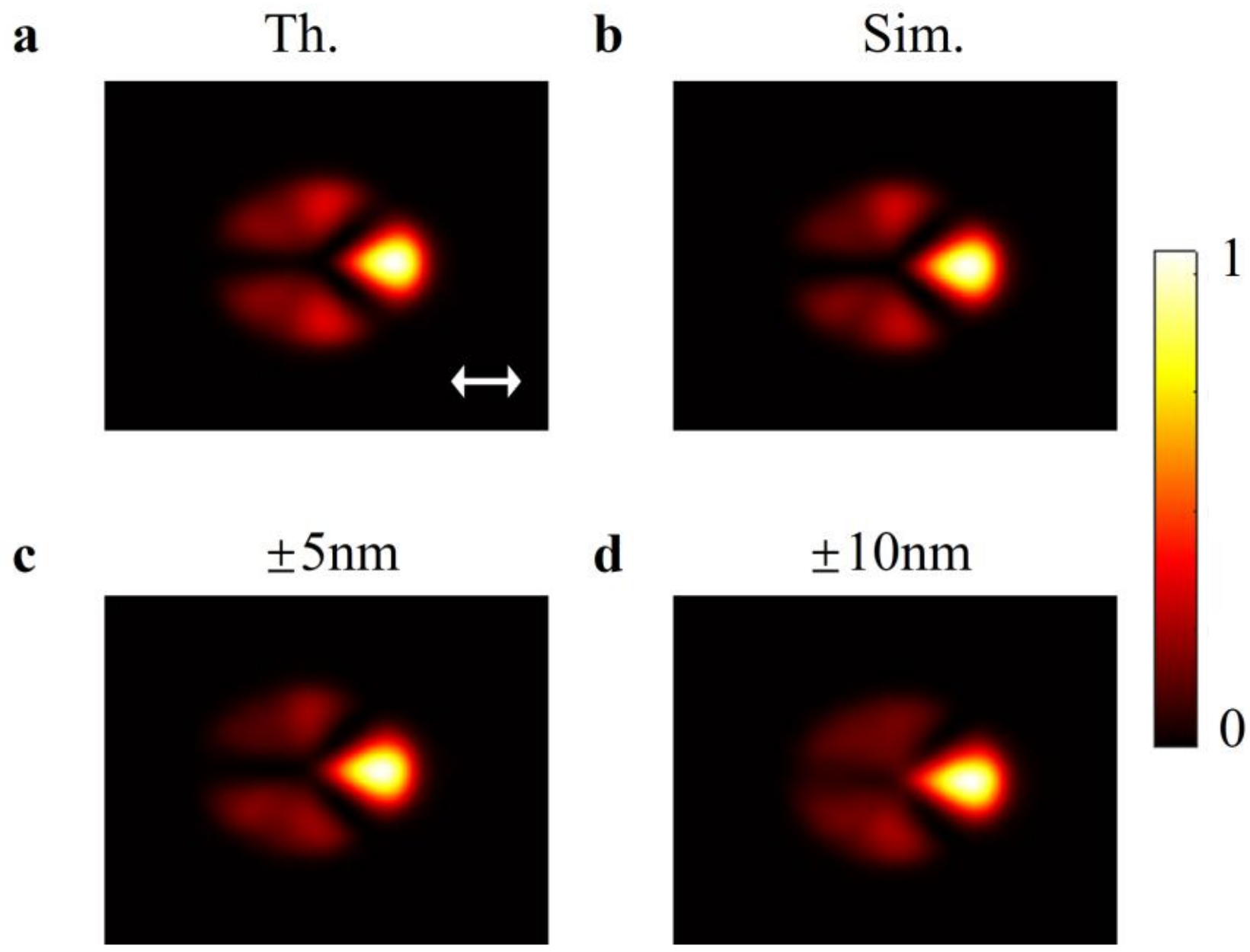

Figure S9 Far-field intensity of the eight-dimensional spin–orbit state under x-polarized projection. **a.** Theoretical result of the target state. **b.** Corresponding calculated result. **c, d.** Calculated results with fabrication perturbations, where the length and width of each meta-atom are independently and randomly varied with equal probability from $\{-5\text{ nm}, 0, +5\text{ nm}\}$ in c and $\{-10\text{ nm}, -5\text{ nm}, 0, +5\text{ nm}, +10\text{ nm}\}$ in d, respectively.

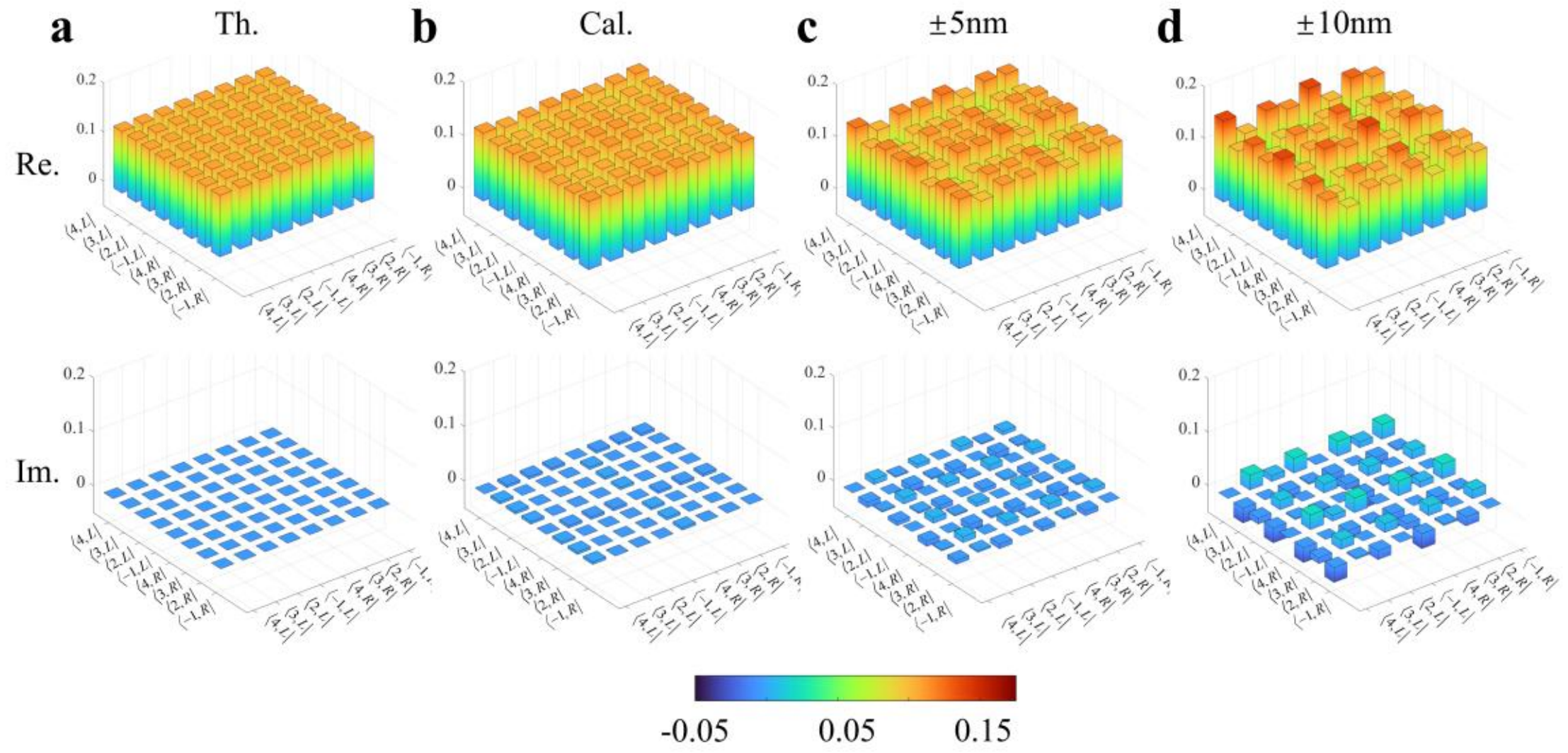

Figure S10 Density matrix results for the eight-dimensional spin–orbit state. **a.** Theoretical density matrix. **b.** Calculated density matrix. **c., d.** Calculated density matrices under fabrication perturbations corresponding to Figure S9 c, d, respectively.
