# Supplementary material for: Generic generation and manipulation of high-dimensional spin-orbit states in Hilbert space
Source: Nat Commun. 2026 Jun 24;17:7936. doi: 10.1038/s41467-026-74192-9 (PMC13448707; doi:10.1038/s41467-026-74192-9)
Supplement: Supplementary file 2 [file 41467_2026_74192_MOESM2_ESM.pdf]

Peer Review File

Generic generation and manipulation of high-dimensional spin-orbit states in Hilbert space

Corresponding Author: Professor Shuang Zhang

This file contains all reviewer reports in order by version, followed by all author rebuttals in order by version.

Version 0:

Reviewer comments:

Reviewer #1

(Remarks to the Author)
The manuscript entitled "Generic generation and manipulation of high-dimensional spin-orbit states in Hilbert space" by Li et al. demonstrates generic methods for the generation, manipulation, and detection of high-dimensional spin–orbit states, both theoretically and experimentally. In addition, the authors identify a special parity structure in high-dimensional Hilbert space, which determines the symmetry of the optical fields. High-dimensional optical fields involving both spin and orbital angular momentum are widely believed to be crucial for quantum information technology, high-precision metrology, and high-capacity optical communication systems. The general ideas are novel, and the presentation is of good scientific quality. I believe this manuscript would be suitable for publication in Nature Communications, provided that the following concerns and comments are properly addressed.
1. In line 27, it is not fair to describe microlasers as having a bulky footprint due to spatially separated basis. The current distance between the two microring lasers (i.e., basis) is around 70 μm, and this distance can be easily reduced to tens of microns if desired. The microlaser itself is about 7 μm in diameter. While this may be larger than some metasurface designs, it should not be considered bulky.
2. Readers may wish to grasp the overall concept of the manuscript clearly from Figure 1. However, the current Figure 1 appears confusing to me. In particular, panels (b) and (c) are problematic: panel (b) is too simplistic to convey useful information, while panel (c) appears somewhat overcrowded.
3. A clear SEM image with a scale bar is necessary if the authors wish to claim the compactness of their devices.
4. The proposed detection method relies on Fourier transformation, which requires additional effort in data processing. It would be helpful if the authors could compare the advantages and disadvantages of this method with those of conventional projection-based detection methods.
5. Regarding the tunability of states in high-dimensional space, for a single fabricated sample, how much area can be covered on the surfaces of HOPS I, II, and III?
6. Does the current design support the generation of states in an even higher-dimensional Hilbert space?
7. The description of the Fourier-transformation-based detection method in the Supporting Information could be more complete. It would be beneficial if the authors could include a full data-processing case study as an example.
8. There is a minor formatting issue in Figure S1c.
9. It would be better to add color bars to all light field images.
10. The fonts in Figure 3 are too small.
11. In Figure 1c and Figure S6, the authors may consider including one reference axis and marking the corresponding rotation angles to help readers more easily identify the symmetries.

Reviewer #2

(Remarks to the Author)
The authors demonstrate a metasurface for generating hybrid spin-orbit optical states in a four-dimensional Hilbert space, with scalability toward higher dimensionality. They further introduce an interferometric reconstruction scheme that can efficiently characterize the full density matrix of these states experimentally. From a physics perspective, the work uncovers a symmetry invariant (spin-orbit parity order) and explores phenomena associated with geometric (topological) phases. Overall, the results are innovative, the physics is rigorous, and the presentation is generally clear. I recommend the manuscript for publication after minor revision.

Major comments:
My main question concerns, for metasurfaces designed following your strategy, how densely the generated states can span the parameter space (the Poincaré hypersphere), and how finely such states can be resolved and characterized experimentally.
Specifically, the four demonstrated examples correspond to points that are well separated on the Poincaré hypersphere. This raises the potential concern: when scaling the encoding strategy to higher dimensions, does the requirement of large separation imply that the encoding becomes highly discrete (although the total number of available states still scales exponentially)?

You state that "Meanwhile, higher dimensionality can be realized by superposing orthogonal spin–orbit bases with different weights using a single metasurface that implements the corresponding Jones matrix. Hence, such method is universal and scalable."
This claim is strong. In practice, there are additional limitations on scalability (e.g., fabrication tolerances and cross-talk between modes). I would encourage you to discuss these limits and their implications for the metasurface design and the interferometric characterization experiment, potentially in the Supplementary Information.

Minor comments:
In Fig. 1b, what does “Ref.” above the cube refer to? Please revise the figure.
The manuscript structure does not comply with Nature Communications formatting: section titles such as “Principle” needs to be revised to match journal requirements.
Some wording choices are unnecessarily uncommon (e.g., “nascent”). Please replace these with more standard scientific language to improve readability.

Version 1:

Reviewer comments:

Reviewer #1

(Remarks to the Author)
The current version of the manuscript is now ready for publication.

Reviewer #2

(Remarks to the Author)
The authors have thoroughly addressed all of my comments. I recommend this paper for publication.

Open Access This Peer Review File is licensed under a Creative Commons Attribution 4.0 International License, which permits use, sharing, adaptation, distribution and reproduction in any medium or format, as long as you give appropriate credit to the original author(s) and the source, provide a link to the Creative Commons license, and indicate if changes were made.

In cases where reviewers are anonymous, credit should be given to 'Anonymous Referee' and the source.
The images or other third party material in this Peer Review File are included in the article's Creative Commons license, unless indicated otherwise in a credit line to the material. If material is not included in the article's Creative Commons license and your intended use is not permitted by statutory regulation or exceeds the permitted use, you will need to obtain permission directly from the copyright holder.
To view a copy of this license, visit https://creativecommons.org/licenses/by/4.0/

The Response to the Reviewers' Comments

Title: Generic generation and manipulation of high-dimensional spin-orbit states in Hilbert space

Authors: Peijin Li, Ruizhe Zhao, Yang Cui, Guangzhou Geng, Junjie Li, Xifeng Ren, Yunkun Wu, Yongtian Wang, Shuang Zhang, Lingling Huang

REVIEWER 1

Reviewer's Comments

The manuscript entitled "Generic generation and manipulation of high-dimensional spin-orbit states in Hilbert space" by Li et al. demonstrates generic methods for the generation, manipulation, and detection of high-dimensional spin–orbit states, both theoretically and experimentally. In addition, the authors identify a special parity structure in high-dimensional Hilbert space, which determines the symmetry of the optical fields. High-dimensional optical fields involving both spin and orbital angular momentum are widely believed to be crucial for quantum information technology, high-precision metrology, and high-capacity optical communication systems. The general ideas are novel, and the presentation is of good scientific quality. I believe this manuscript would be suitable for publication in Nature Communications, provided that the following concerns and comments are properly addressed.

Our Responses

We thank the reviewer for the evaluation of our work and appreciate the detailed comments, which are of great value. We appreciate the valuable suggestions for improving our manuscript.

Reviewer's Comments 1

In line 27, it is not fair to describe microlasers as having a bulky footprint due to spatially separated basis. The current distance between the two microring lasers (i.e.,

basis) is around 70 μm, and this distance can be easily reduced to tens of microns if desired. The microlaser itself is about 7 μm in diameter. While this may be larger than some metasurface designs, it should not be considered bulky.

Our Responses

We thank the reviewer for this important comment. Our intended point was not about the absolute footprint of the microlaser itself, but rather the fact that such approaches typically require spatially separated basis generation and an additional external combiner element, in contrast to our single-planar metasurface implementation. We have revised the relevant sentence to avoid confusion.

"Despite progress in on-chip synthesis techniques for dimensionality expansion, current methods either rely on combination of spatially separated basis[1], or cannot fully cover the Hilbert space[2], restricting the generation of arbitrary high-dimensional spin–orbit states."

Reviewer's Comments 2

Readers may wish to grasp the overall concept of the manuscript clearly from Figure 1. However, the current Figure 1 appears confusing to me. In particular, panels (b) and (c) are problematic: panel (b) is too simplistic to convey useful information, while panel (c) appears somewhat overcrowded.

Our Responses

We sincerely appreciate your valuable suggestion. We have revised the Fig.1. In the revised figure, panel (b) is redrawn to show the interferometric characterization procedure more explicitly, so that the detection concept can be understood more intuitively. Panel (c) is simplified by keeping only one representative example. Auxiliary arrows and reference markings are added to make the symmetry in the far-field patterns easier to recognize.

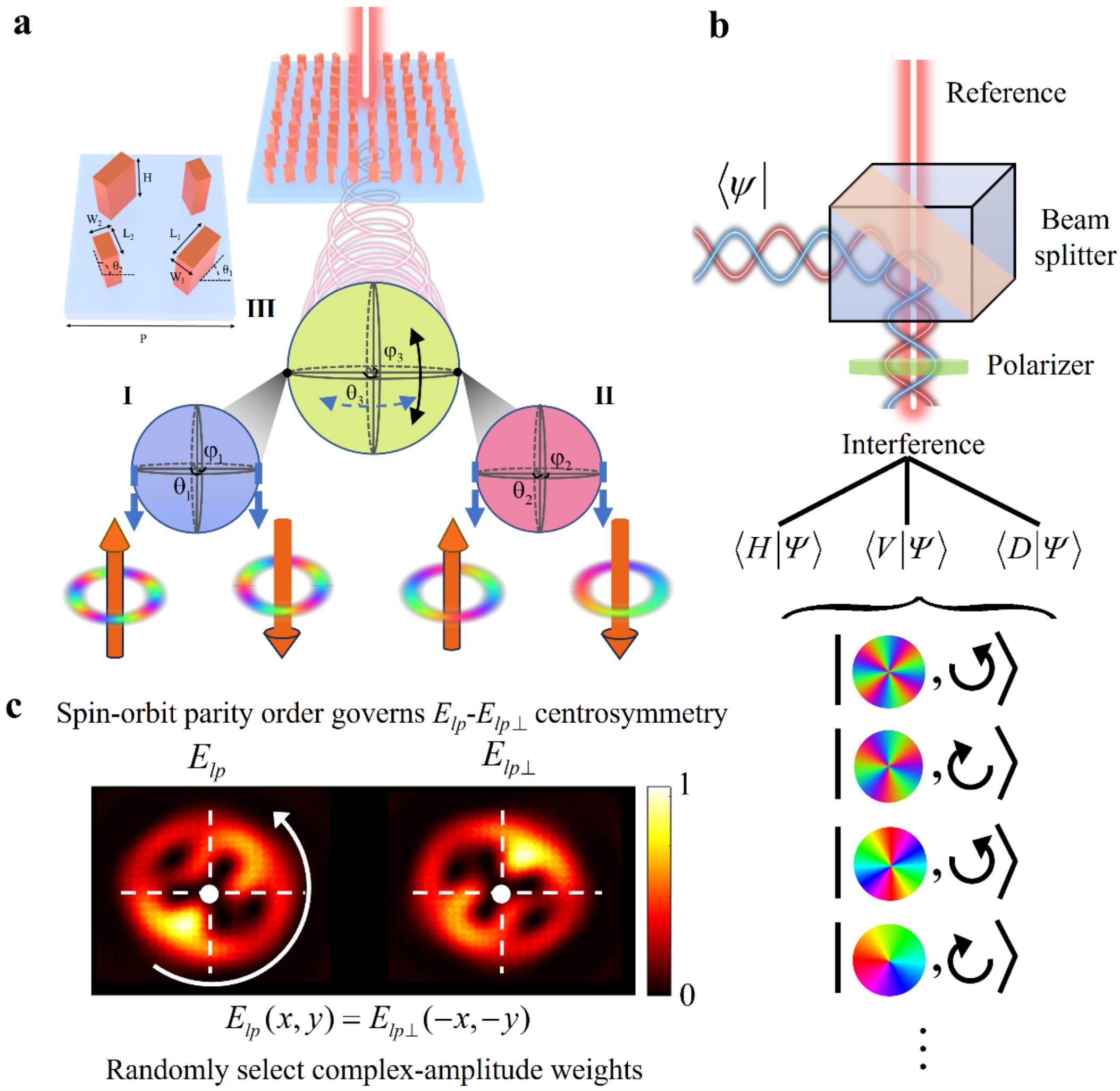

Fig.1. Generation, detection and parity order of high-dimensional spin–orbit states. a, The generation of high-dimensional spin–orbit states via metasurface. **b,** The schematic of the interferometry–projection–tomography reconstruction process. The generated state $|\psi\rangle$ interferes with a reference beam, and the interferograms recorded under the H, V, and D polarization projections are used to mode decomposition and recover the high-dimensional spin-orbit state. **c,** The concept of the spin–orbit parity order: the ordered arrangement of spin and OAM in the eigenmodes of the high-dimensional spin–orbit space induces a centrosymmetric feature in all states under linear polarization projections. As illustrated, the eigenmodes $|4,L\rangle,|3,R\rangle,|2,L\rangle,|-1,R\rangle,$

corresponding to parity order parameter P=1. A randomly selected state in this space, $|\psi\rangle = (-0.30+0.82i)|4,L\rangle - 0.05i|3,R\rangle + (0.33+0.22i)|2,L\rangle + (0.17-0.21i)|-1,R\rangle$, exhibit centrosymmetric far-field projections under both x- and y-linear polarization bases.

Reviewer's Comments 3

A clear SEM image with a scale bar is necessary if the authors wish to claim the compactness of their devices.

Our Responses

Thank you for this helpful suggestion. We have added a clear SEM image of the fabricated Sample #1 with a scale bar in Supplementary Note 1.

b

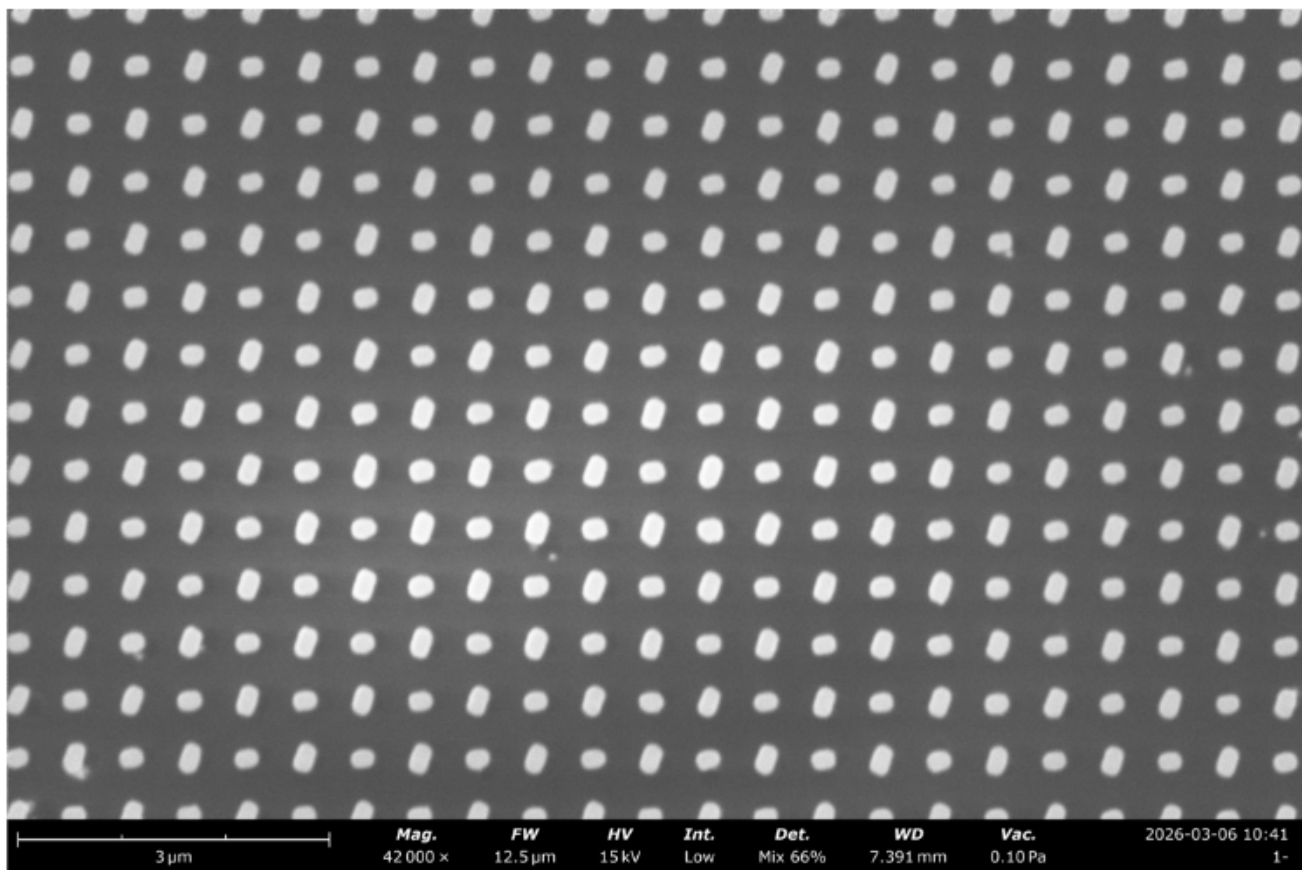

Figure S1 **b.** The scanning electron microscopy image of Sample #1, with the scale bar of 3 μm.

Reviewer's Comments 4

The proposed detection method relies on Fourier transformation, which requires additional effort in data processing. It would be helpful if the authors could compare the advantages and disadvantages of this method with those of conventional projection-based detection methods.

Our Responses

We thank the reviewer for this helpful comment. In conventional projection-based

detection, the unknown state is projected onto a set of analysis bases, and the corresponding intensities are recorded. Full state reconstruction of an N-dimensional state typically requires repeated measurements over at least 2N-1 bases. Therefore, the measurement number increases with the state dimension.

By contrast, the main **advantage** of our method is its **measurement efficiency:** in our implementation, only **three interferograms** corresponding to the H, V, and D projections are required for state reconstruction. The **cost** is that the method requires **additional Fourier-domain post-processing** to recover the complex field from each interferogram.

We would like to clarify that, although our method requires recording multiple interferograms, it does not require strict phase locking between different measurements. Because each projection channel is reconstructed independently from a single off-axis interferogram, the reconstruction does not rely on maintaining a fixed phase relationship across multiple exposures. This makes the approach experimentally convenient and robust.

Reviewer's Comments 5

Regarding the tunability of states in high-dimensional space, for a single fabricated sample, how much area can be covered on the surfaces of HOPS I, II, and III?

Our Responses

We thank the reviewer for this valuable question. For a single fabricated sample, the generated 4D spin–orbit state remains continuously tunable through three external degrees of freedom: (i) the incident polarization ellipticity θ_p , (ii) the incident polarization azimuth φ_p, and (iii) the metasurface rotation angle φ_r.

Varying θ_p changes the latitude (polar angle) on the HOPS I–III. Tuning φ_p and the metasurface rotation φ_r can both rotate the longitudes on the HOPS I–III. However, metasurface rotation acts through mode-dependent higher-order PB phases (Eq. (6), see Fig. 5a), and therefore yields a different set of relative longitude rotations among the

three HOPS compared with φ_p tuning.

Hence, the three external degrees of freedom provide **three independently addressable coordinates** on the Poincaré hypersphere. A convenient independent set can be chosen as the polar angle and azimuthal angle (θ_1, φ_1) on HOPS I (controlled by θ_p and φ_p) and an additional **independently tunable** azimuthal angle φ_2 on HOPS II (controlled by φ_p and φ_r). The remaining hyperspherical parameters are **dependent variables** once $(\theta_1, \varphi_1, \varphi_2)$ are specified.

Taking the Sample #3 as an example, Supplementary Equation (S34) gives:

$$\begin{cases} \theta_1' = 2\arctan\left[\tan(\frac{\theta_1}{2})\frac{A_2}{A_1}\right] \\ \varphi_1' = \varphi_1 + \frac{(\sigma_1 - \sigma_2)}{2}\varphi_p \\ \varphi_2' = \varphi_2 + \frac{(\sigma_3 - \sigma_4)}{2}\varphi_p \end{cases} \tag{R1}$$

For Sample #3, $A_1 = A_2$, $\sigma_1 = \sigma_3 = 1$, $\sigma_2 = \sigma_4 = -1$, $\theta_1 = \frac{\pi}{2}$ and $\varphi_1 = \varphi_2 = 0$. Therefore, when only the incident polarization is varied, we obtain $\theta_1' = \theta_p$, $\varphi_1' = \varphi_p$ $\varphi_2' = \varphi_p$. Meanwhile, from Eq. (6) in the main text, only varying the metasurface rotation angle gives:

$$\begin{cases} \varphi_1' = \varphi_1 + (l_1 - l_2 + 2\sigma_1 - 2\sigma_2)\varphi_r \\ \varphi_2' = \varphi_2 + (l_3 - l_4 + 2\sigma_3 - 2\sigma_4)\varphi_r, \end{cases} \tag{R2}$$

Since $l_1 = 4$, $l_2 = 3$, $l_3 = 2$, $l_4 = -1$, varing φ_r alone leads to $\varphi_1' = 5\varphi_r$, $\varphi_2' = 7\varphi_r$ Combining the two controls, the hyperspherical parameters are explicitly related to the external controls as:

$$\theta_1' = \theta_p, \quad \varphi_1' = 5\varphi_r + \varphi_p, \quad \varphi_2' = 7\varphi_r + \varphi_p \tag{R3}$$

Hence, $(\theta_1, \varphi_1, \varphi_2)$ can be continuously and independently addressed. In other words, a single sample enables continuous coverage on HOPS I, while simultaneously scanning the longitudes on HOPS II and inducing corresponding continuous variation

on HOPS III.

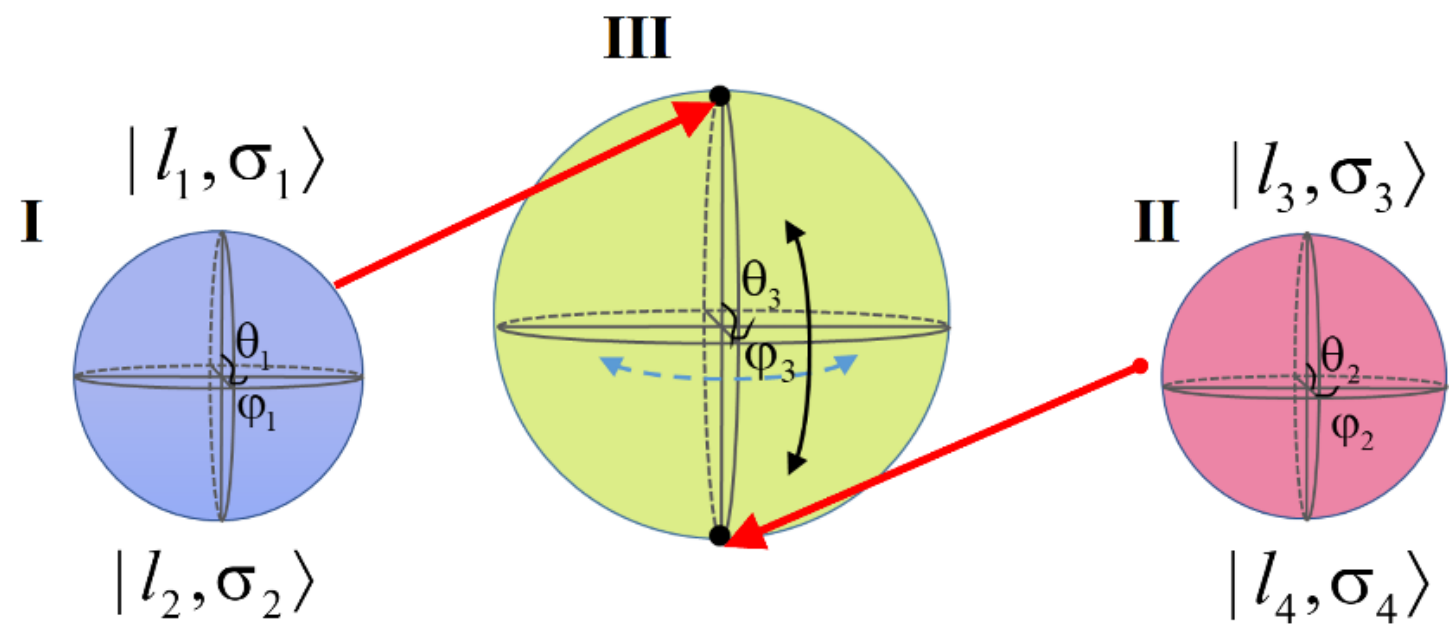

Fig.R1. Poincaré hypersphere representation of the four-dimensional spin-orbit Hilbert space.

Reviewer's Comments 6

Does the current design support the generation of states in an even higher-dimensional Hilbert space?

Our Responses

We thank the reviewer for this important question. The current design indeed supports the generation of states in an even higher-dimensional Hilbert space. The proposed approach is based on a general Jones-matrix framework and is not restricted to the four-dimensional case demonstrated in the main text. To clarify this point, we have added a new section, *"**Scalability to higher-dimension**"*, in the Supplementary Information. In this section, we provide a numerical demonstration of a representative eight-dimensional spin–orbit state. The calculated far-field intensity agrees well with the theoretical target, and the corresponding density matrix also reproduces the target state with high accuracy. The calculated fidelity reaches 99.9%, which supports that the present design strategy can be extended to a higher-dimensional Hilbert space.

"The Jones-matrix-based framework used in this work is not restricted to the four-dimensional case. In principle, higher-dimensional spin–orbit states can be generated by incorporating additional orthogonal spin–orbit eigen-components with prescribed complex weights into the target Jones matrix. To illustrate this scalability, we numerically designed a representative eight-dimensional spin–orbit state:

$$|\Psi\rangle = \frac{1}{2\sqrt{2}}(|4,L\rangle + |3,L\rangle + |2,L\rangle + |-1,L\rangle + |4,R\rangle + |3,R\rangle + |2,R\rangle + |-1,R\rangle) \quad \text{(S42)}$$

Figure S9a presents the theoretical far-field intensity under x-polarized illumination and x-polarized projection, and Figure S9b shows the corresponding calculated result after mapping the ideal continuous design onto a discrete practical meta-atom library. The good agreement between the two is further supported by the density matrix shown in Figure S10, where the calculated density matrix in Figure S10b closely matches the theoretical target in Figure S10a. The corresponding fidelity reaches 99.9%, confirming that the present Jones-matrix-based design strategy can be extended beyond the four-dimensional Hilbert space demonstrated in the main text.

To evaluate the practical scalability, we further introduced fabrication-related perturbations into the metasurface design. Specifically, for each pixel, the length and width of the meta-atom are independently perturbed by random values chosen with equal probability from{-5nm，0，+5nm} and{-10nm，-5nm，0，+5nm，+10nm}.When increasing perturbation level, the generated far-field electromagnetic field shows increasing deviation from the target state, as shown in Figure S9c, d. The similar trend is observed in the reconstructed density matrices in Figure S10c, d. From the density matrices, the real part progressively departs from the theoretical distribution, while non-ideal imaginary components emerge, indicating increased inter-mode crosstalk. Accordingly, the state fidelity decreases to 99.5% for the ±5 nm perturbation case, and 98.2% for the ±10 nm perturbation case. Nevertheless, the fidelity remains high in both cases, indicating that the proposed design retains good robustness against moderate fabrication perturbations.”

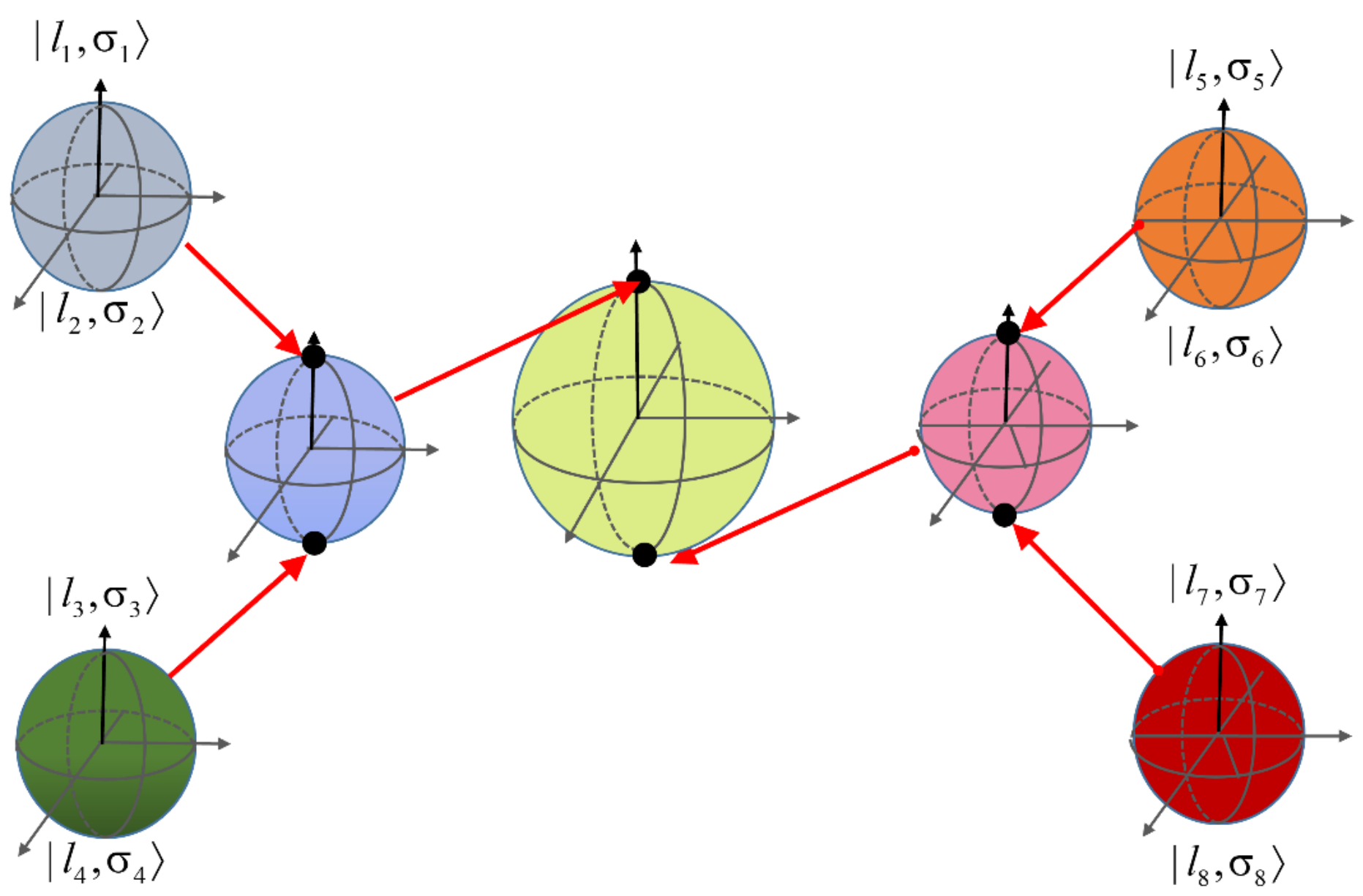

Figure S8 Poincaré hypersphere representation of the eight-dimensional spin-orbit Hilbert space.

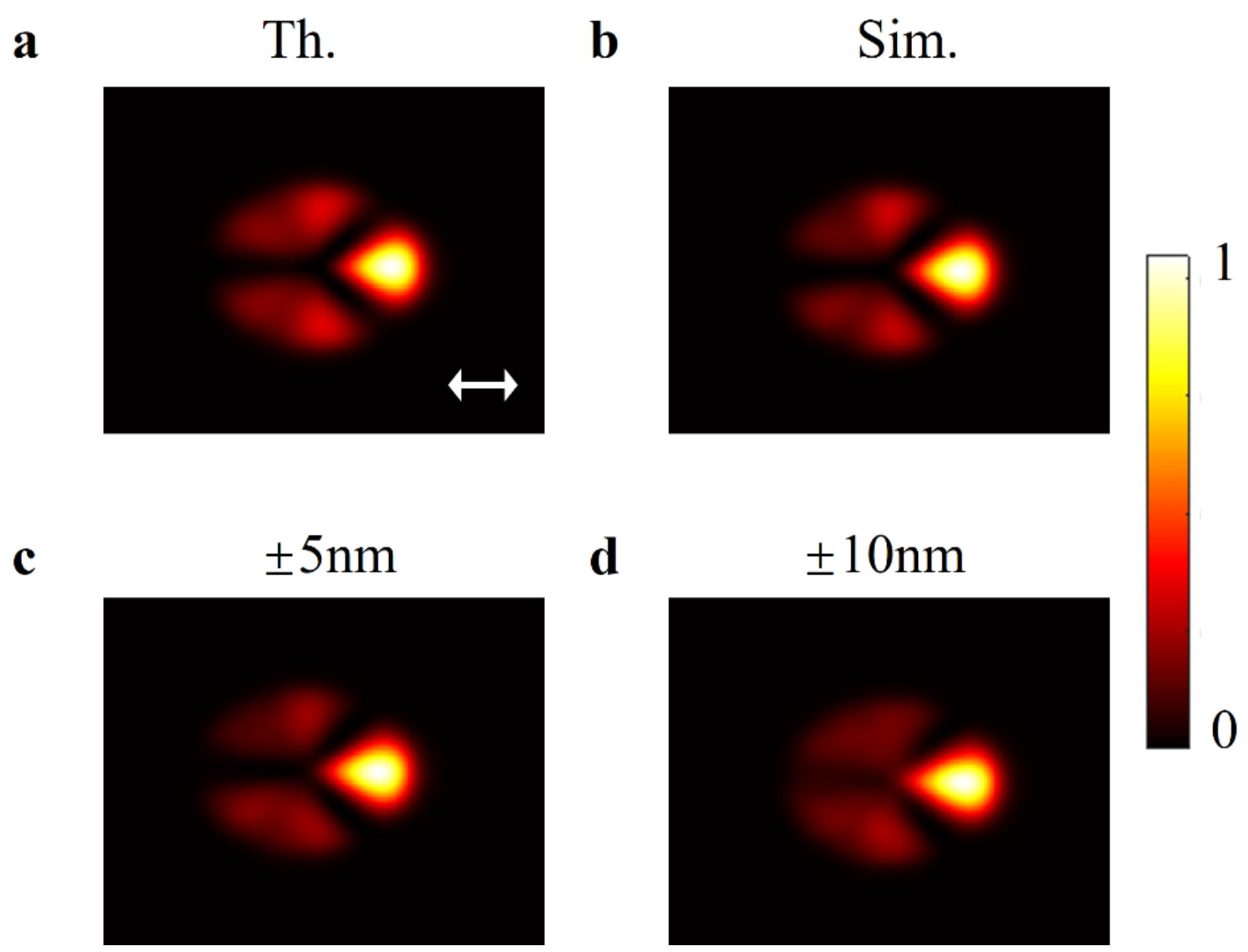

Figure S9 Far-field intensity of the eight-dimensional spin–orbit state under x-polarized projection. **a.** Theoretical result of the target state. **b.** Corresponding calculated result. **c, d.** Calculated results with fabrication perturbations, where the length and width of each meta-atom are independently and randomly varied with equal probability from {−5 nm,0, +5 nm}in c and {−10 nm, −5 nm, 0, +5 nm, +10 nm} in d, respectively.

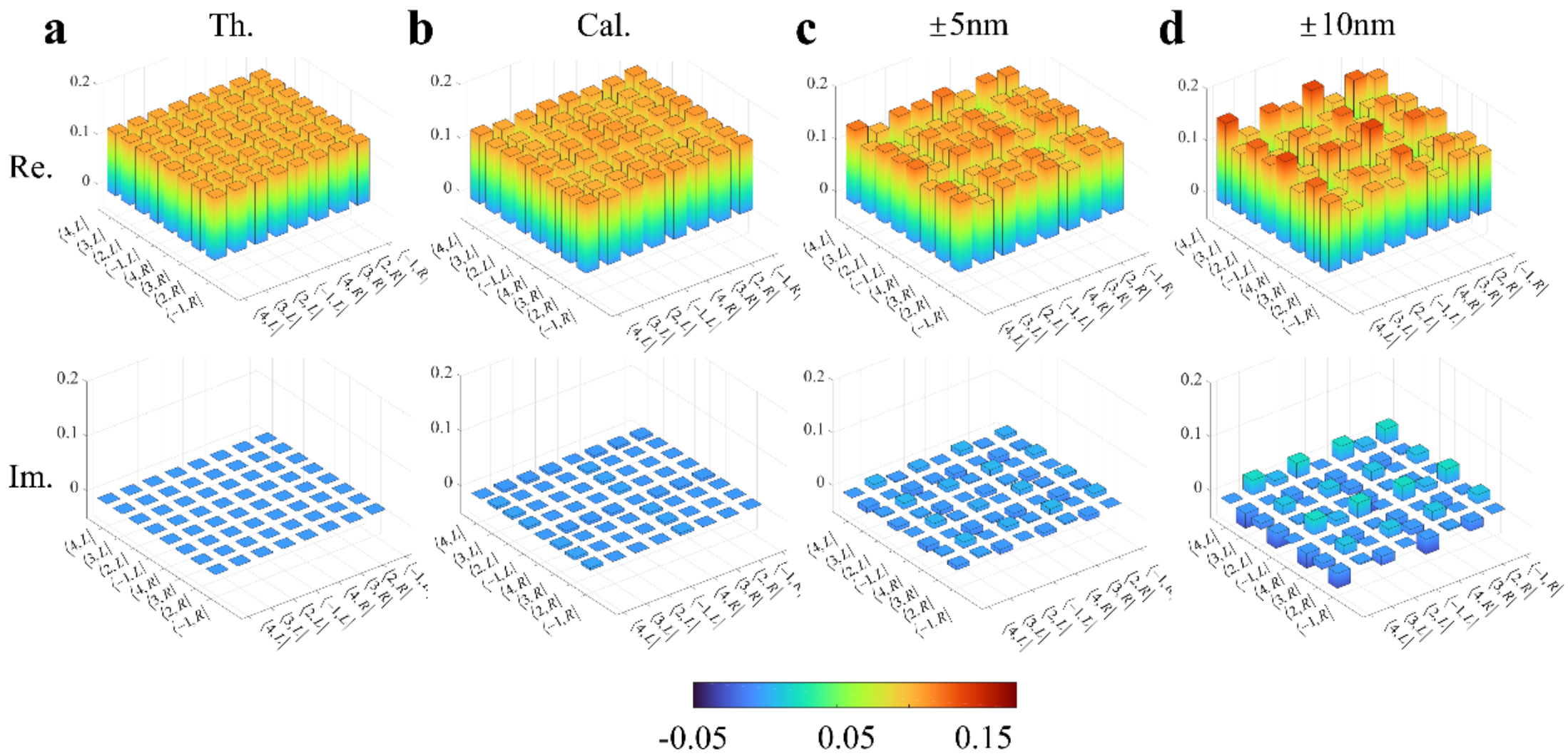

Figure S10 Density matrix results for the eight-dimensional spin–orbit state. **a.** Theoretical density matrix. **b.** Calculated density matrix. **c., d.** Calculated density matrices under fabrication perturbations corresponding to Figure S9 c, d, respectively.

Reviewer's Comments 7

The description of the Fourier-transformation-based detection method in the Supporting Information could be more complete. It would be beneficial if the authors could include a full data-processing case study as an example.

Our Responses

The reviewer's suggestion is very valuable. In the revised Supplementary Information, we have expanded the description of the Fourier-transformation-based detection method in Supplementary Note 2 to make the data-processing procedure more complete.

“ $$I(x,y)=|E_s(x,y)+E_r(x,y)|^2=|E_s|^2+|E_r|^2+E_sE_r^*e^{i\Delta k\cdot r}+E_s^*E_re^{-i\Delta k\cdot r} \quad \text{(S6)}$$

By performing a 2D Fourier transform of $I(x,y)$, we have

$$\tilde{I}(k)=\mathrm{F}\{|E_s|^2\}(k)+\mathrm{F}\{|E_r|^2\}(k)+\mathrm{F}\{E_sE_r^*\}(k-\Delta k)+\mathrm{F}\{E_s^*E_r\}(k+\Delta k) \quad \text{(S7)}$$

The first two terms form a dominant DC component centered at the origin, while the last two terms correspond to two symmetrically displaced sidebands originating

from the cross term and its complex conjugate. The off-axis carrier $\Delta\mathbf{k}$ is chosen such that the sidebands are well separated from the DC term. We isolate one sideband using a circular band-pass mask covering the selected sideband peak; the mask radius is chosen to fully include the signal bandwidth while avoiding overlap with the DC and the conjugate sideband. The filtered sideband is then shifted to the origin and inverse Fourier transformed to recover the complex cross term $E_s E_r^*$.

$$E_s E_r^* = |E_s||E_r| e^{i(\phi_{sample}+\phi_{system}-\phi_r)} \tag{S8}$$

To remove the system background and eliminate the reference-field contribution, a background measurement is taken without the sample in advance to obtain the $\phi_{system} - \phi_r$. Then the sample-induced complex field can be written as:

$$E_{\text{sample}}(x,y) = \frac{E_s(x,y)E_r^*(x,y)}{|E_r(x,y)| e^{i(\phi_{system}-\phi_r)}} \tag{S9}$$

The same procedure is applied to each polarization projection (H/V/D) to obtain the complex fields E_H, E_v, E_D, which are then used for the subsequent mode decomposition and state reconstruction."

In addition, following the reviewer's suggestion, we have added a full data-processing case study using Sample #1 as a representative example.

"As a representative example, we present here the complete data-processing procedure for Sample #1, following exactly the interferometry–projection–tomography method described above. According to Eq. (S10), the spin–orbit state can be expressed in the full measurement basis as:

$$\begin{aligned} |\psi\rangle &= a_1|2,L\rangle + b_1|2,R\rangle + a_2|-2,L\rangle + b_2|-2,R\rangle \\ &\quad + a_3|-1,L\rangle + b_3|-1,R\rangle + a_4|1,L\rangle + b_4|1,R\rangle \end{aligned} \tag{S14}$$

For this sample, three off-axis interferograms are recorded under the horizontal, vertical, and diagonal polarization projections, respectively. For each projection, the interferogram is processed by a two-dimensional Fourier transform, sideband filtering, shifting to the origin, and inverse Fourier transform, so that the corresponding complex field distributions $\langle H|\Psi\rangle$, $\langle V|\Psi\rangle$ and $\langle D|\Psi\rangle$ are retrieved. These reconstructed complex

fields are then decomposed into the OAM eigenmodes $|l=\pm 2\rangle$ and $|l=\pm 1\rangle$. From Eq. (S11)–(S13), the measured modal coefficients give the following set of relative equations:

$$\begin{aligned}
&\frac{a_2+b_2}{a_1+b_1}=1.12\text{ - }0.57\text{i}\ ;\frac{a_3+b_3}{a_1+b_1}=0.20+1.07\text{i}\ ;\frac{a_4+b_4}{a_1+b_1}=0.27+0.96\text{i}\\
&\frac{a_2-b_2}{a_1-b_1}=\text{-}0.86+0.04\text{i};\frac{a_3-b_3}{a_1-b_1}=0.07+0.77\text{i}\ ;\frac{a_4-b_4}{a_1-b_1}=\text{-}0.05\text{ - }0.76\text{i}\\
&\frac{ia_2+b_2}{a_1+b_1}=\ \text{-}0.50\text{ - }1.39\text{i}\ ;\frac{ia_3+b_3}{a_1+b_1}=0.31+0.87\text{i}\ ;\frac{ia_4+b_4}{a_1+b_1}=1.18\text{ - }0.44\text{i}
\end{aligned} \tag{S15}$$

These nine equations form an overdetermined linear system for the seven unknown relative coefficients in the vector $x=[\frac{a_1}{a_1},\frac{b_1}{a_1},\frac{a_2}{a_1},\frac{b_2}{a_1},\frac{a_3}{a_1},\frac{b_3}{a_1},\frac{a_4}{a_1},\frac{b_4}{a_1}]$

By solving this system using a least-squares procedure, we obtain:

$$\begin{aligned}
x=[\ &1,\text{-}0.12\text{ - }0.13\text{i}\ ,\text{-}0.02\text{ - }0.35\text{i},\ 0.93\text{ - }0.28\text{i},\\
&0.15+0.91\text{i},\ 0.15+0.02\text{i},\ 0.21\text{ - }0.04\text{i},\ 0.18+0.85\text{i}]
\end{aligned} \tag{S16}$$

This solution corresponds to the experimentally reconstructed state in the full eight-dimensional measurement space. For Sample #1, however, the designed target state occupies only the four-dimensional subspace spanned by $\{|2,L\rangle,|-2,R\rangle,|-1,L\rangle,|1,R\rangle\}$. Therefore, for direct comparison with the target design, we restrict the reconstructed result to this four-dimensional subspace. The experimentally reconstructed state in the target subspace can then be written as:

$$\begin{aligned}
|\psi_{1\exp}\rangle &= a_1\,|2,L\rangle + b_2\,|-2,L\rangle + a_3\,|-1,L\rangle + b_4\,|1,R\rangle\\
&=|2,L\rangle+(0.93-0.28i)\,|-2,R\rangle+(0.15+0.91i)\,|-1,L\rangle+(0.18+0.85i)\,|1,R\rangle
\end{aligned} \tag{S17}$$

After normalization, it becomes:

$$\begin{aligned}
|\psi_{1\exp}\rangle = {}&0.53\,|2,L\rangle+(0.50-0.15i)\,|-2,R\rangle\\
&+(0.08+0.48i)\,|-1,L\rangle+(0.09+0.45i)\,|1,R\rangle
\end{aligned} \tag{S18}$$

The corresponding experimental density matrix is reconstructed as:

$$\rho_{1\exp} = |\psi_{1\exp}\rangle\langle\psi_{1\exp}| = \begin{bmatrix} 0.28 + 0.00i & 0.26 - 0.08i & 0.04 + 0.26i & 0.05 + 0.24i \\ 0.26 + 0.08i & 0.27 + 0.00i & -0.03 + 0.25i & -0.02 + 0.24i \\ 0.04 - 0.26i & -0.03 - 0.25i & 0.24 + 0.00i & 0.22 - 0.01i \\ 0.05 - 0.24i & -0.02 - 0.24i & 0.22 + 0.01i & 0.21 + 0.00i \end{bmatrix} \quad \text{(S19)}$$

For Sample #1, the theoretical density matrix is:

$$\rho_{1th} = |\psi_{1th}\rangle\langle\psi_{1th}| = \begin{bmatrix} 0.25 & 0.25 & 0.25i & 0.25i \\ 0.25 & 0.25 & 0.25i & 0.25i \\ -0.25i & -0.25i & 0.25 & 0.25 \\ -0.25i & -0.24i & 0.25 & 0.25 \end{bmatrix} \quad \text{(S20)}$$

The fidelity between the reconstructed and theoretical density matrices is then calculated as $F_1 = \left[\mathrm{Tr}\left(\sqrt{\sqrt{\rho_{1\exp}}\,\rho_{1\mathrm{th}}\sqrt{\rho_{1\exp}}}\right)\right]^2 = 0.985$"

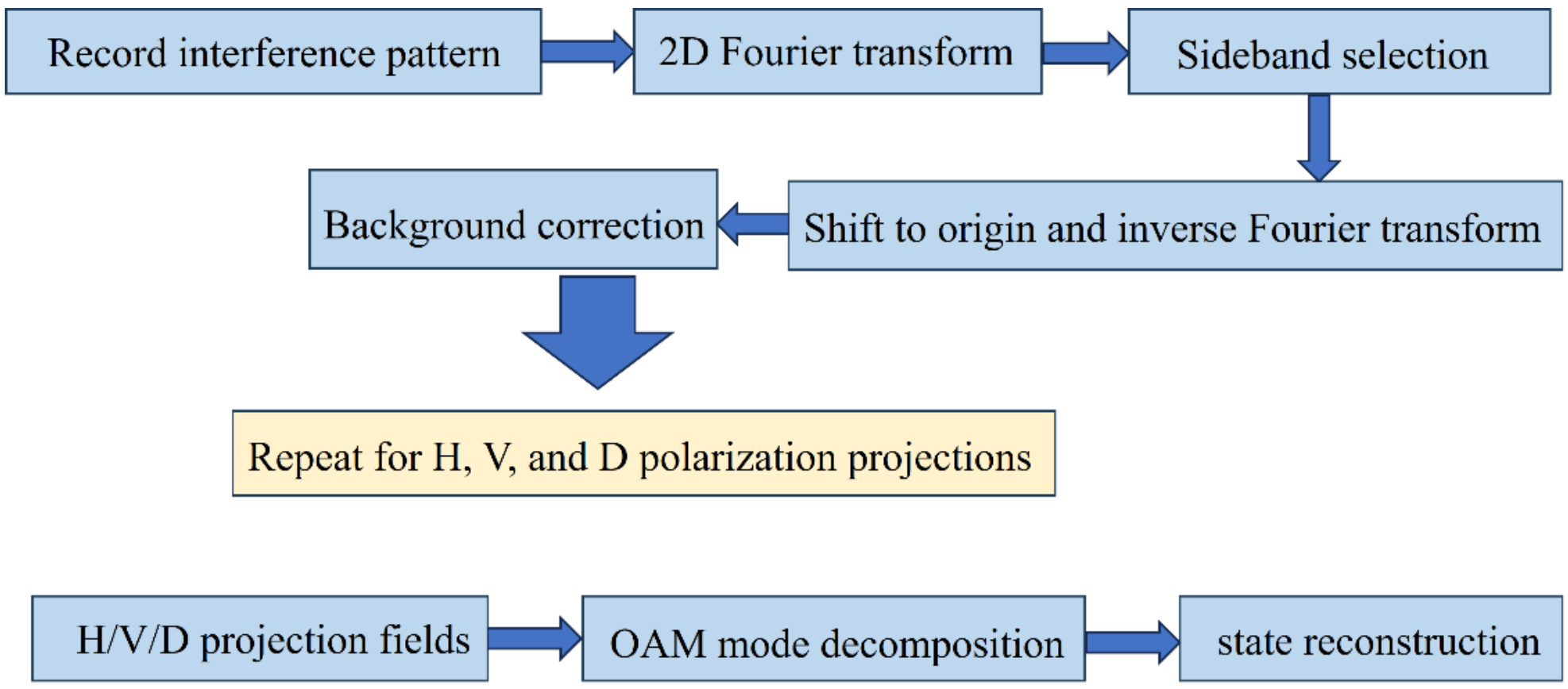

Figure S2. Schematic workflow of the Interferometry-projection-tomograph method.

Reviewer's Comments 8

There is a minor formatting issue in Figure S1c

Our Responses

We sincerely thank the reviewer for the careful observation. We have corrected the this in the revised Supplementary Note 1.

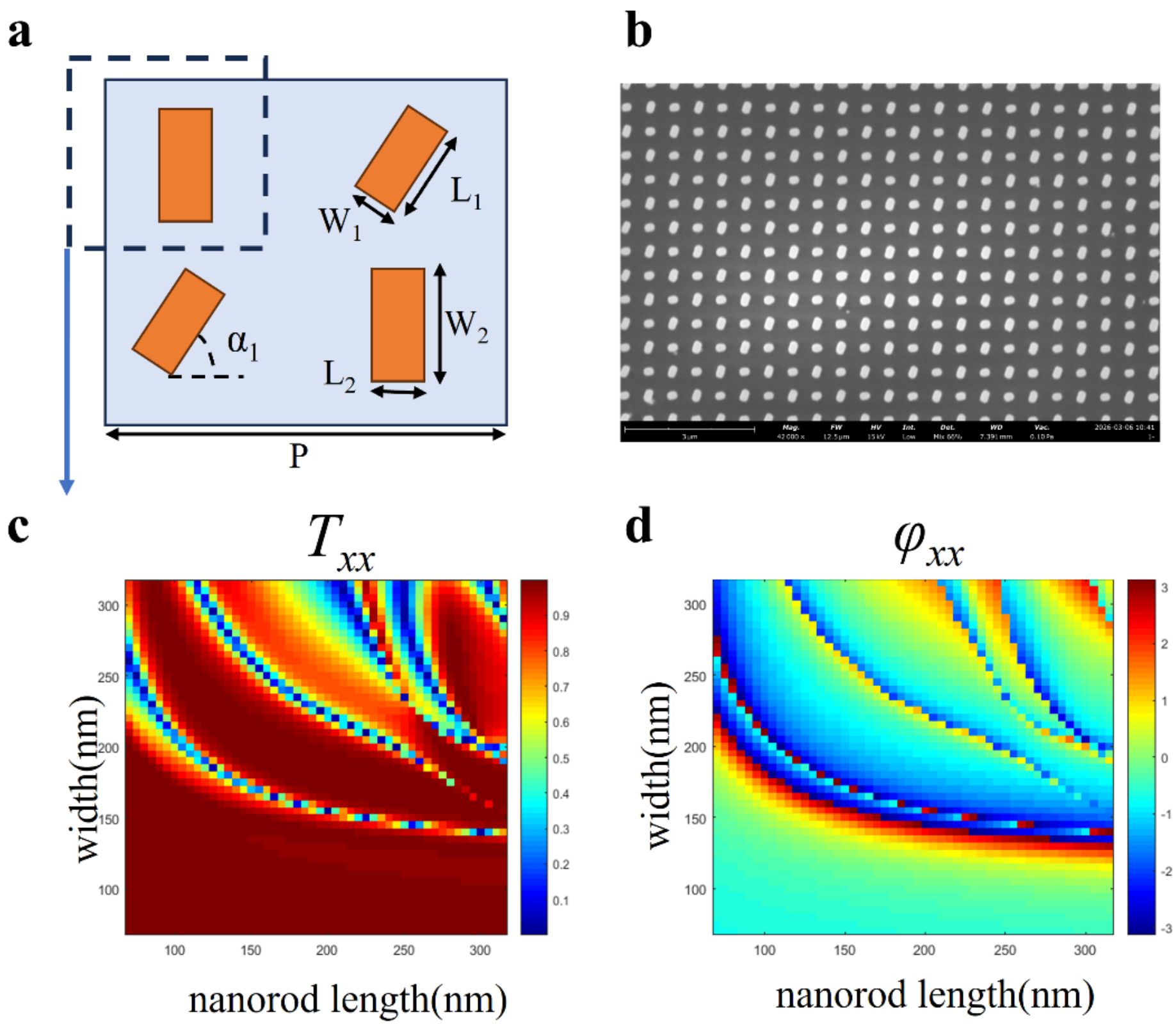

Figure S1. Numerical simulation of meta-atoms within the tetramer.

Reviewer's Comments 9

It would be better to add color bars to all light field images.

Our Responses

We thank the reviewer for this valuable suggestions. We have add color bars in Fig.1, Fig.4, Fig.5 and Figure S7

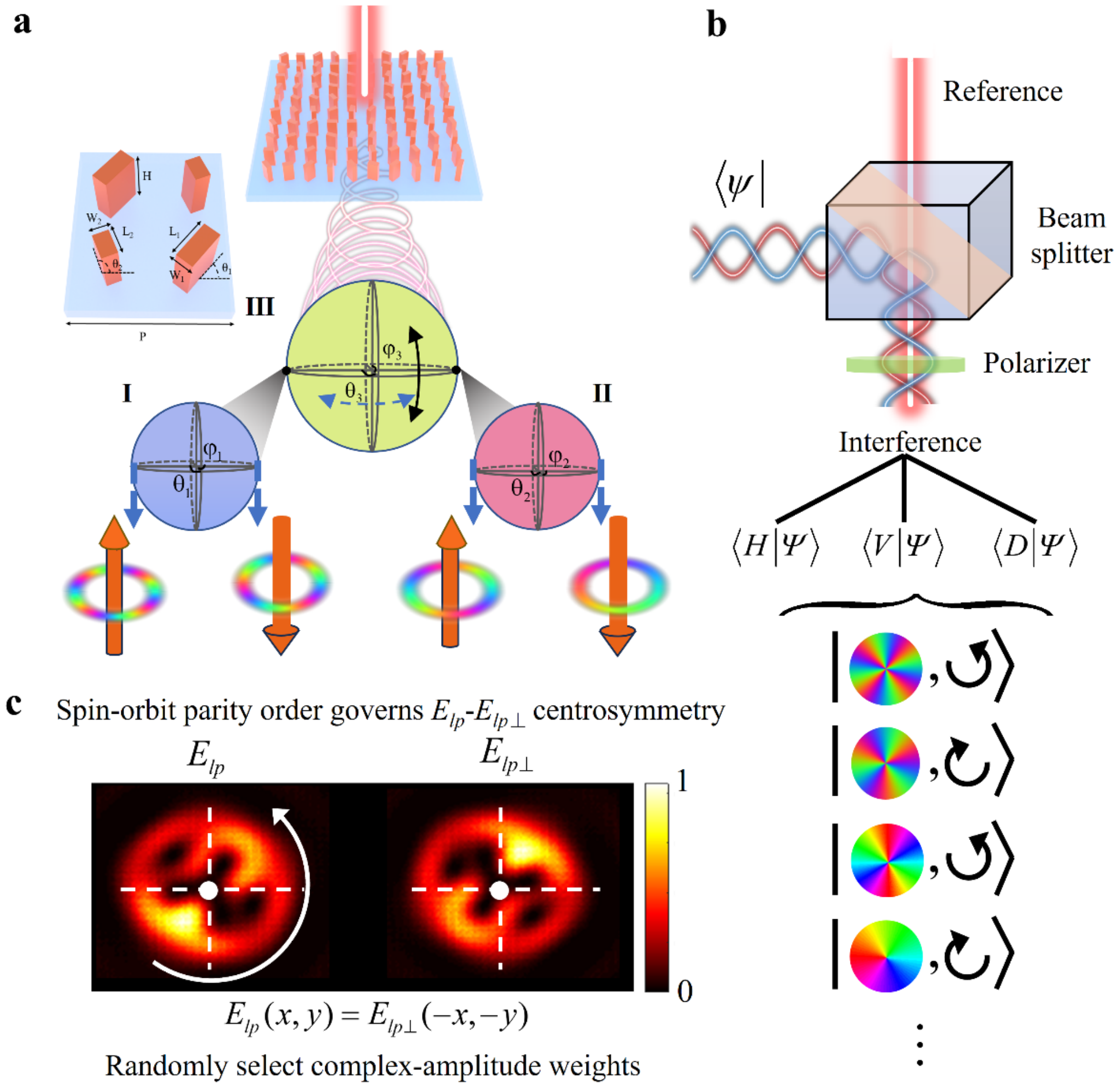

Fig.1. Generation, detection and parity order of high-dimensional spin–orbit states.

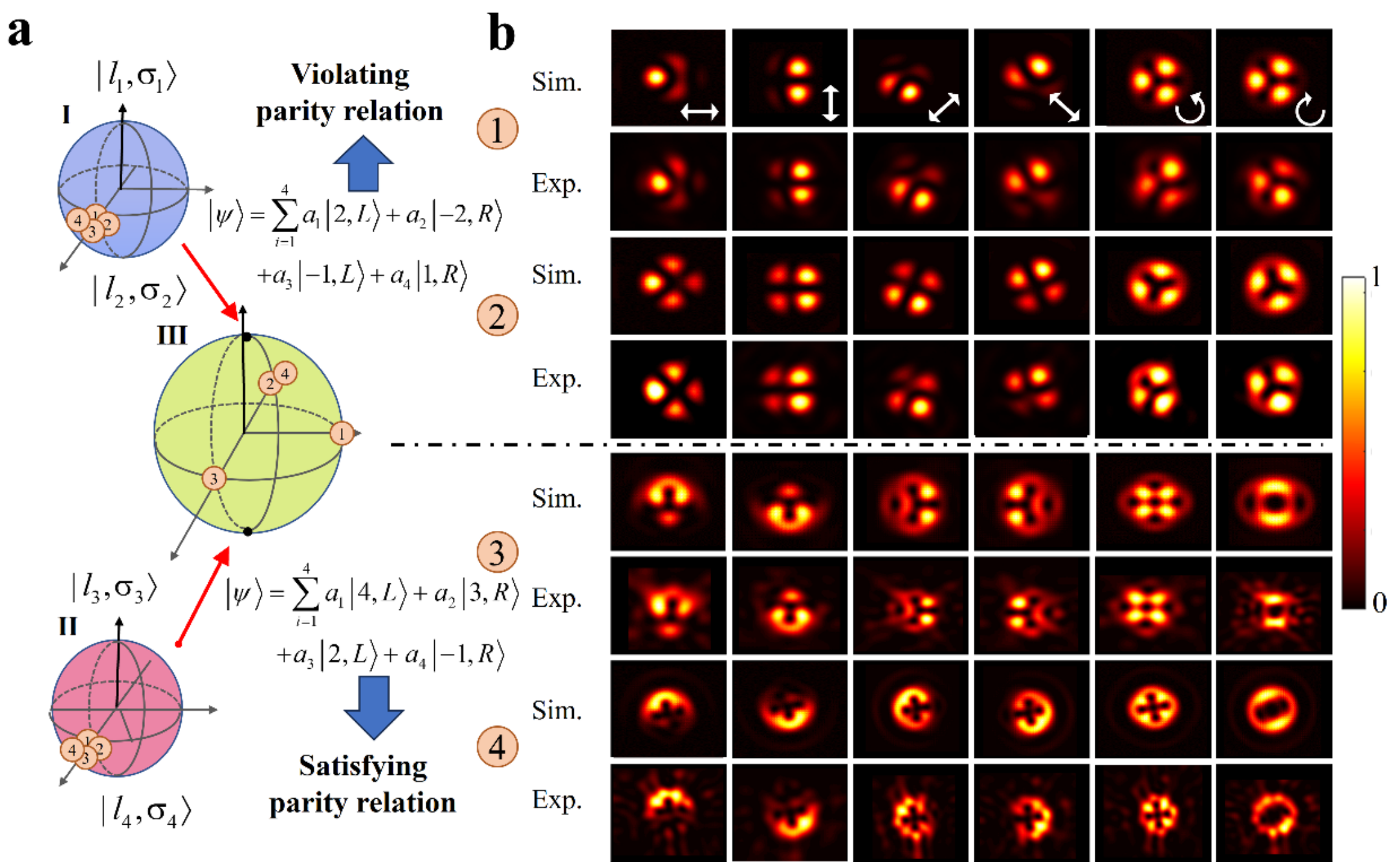

Fig.4. Spin–orbit parity order and centrosymmetry in high-dimensional states.

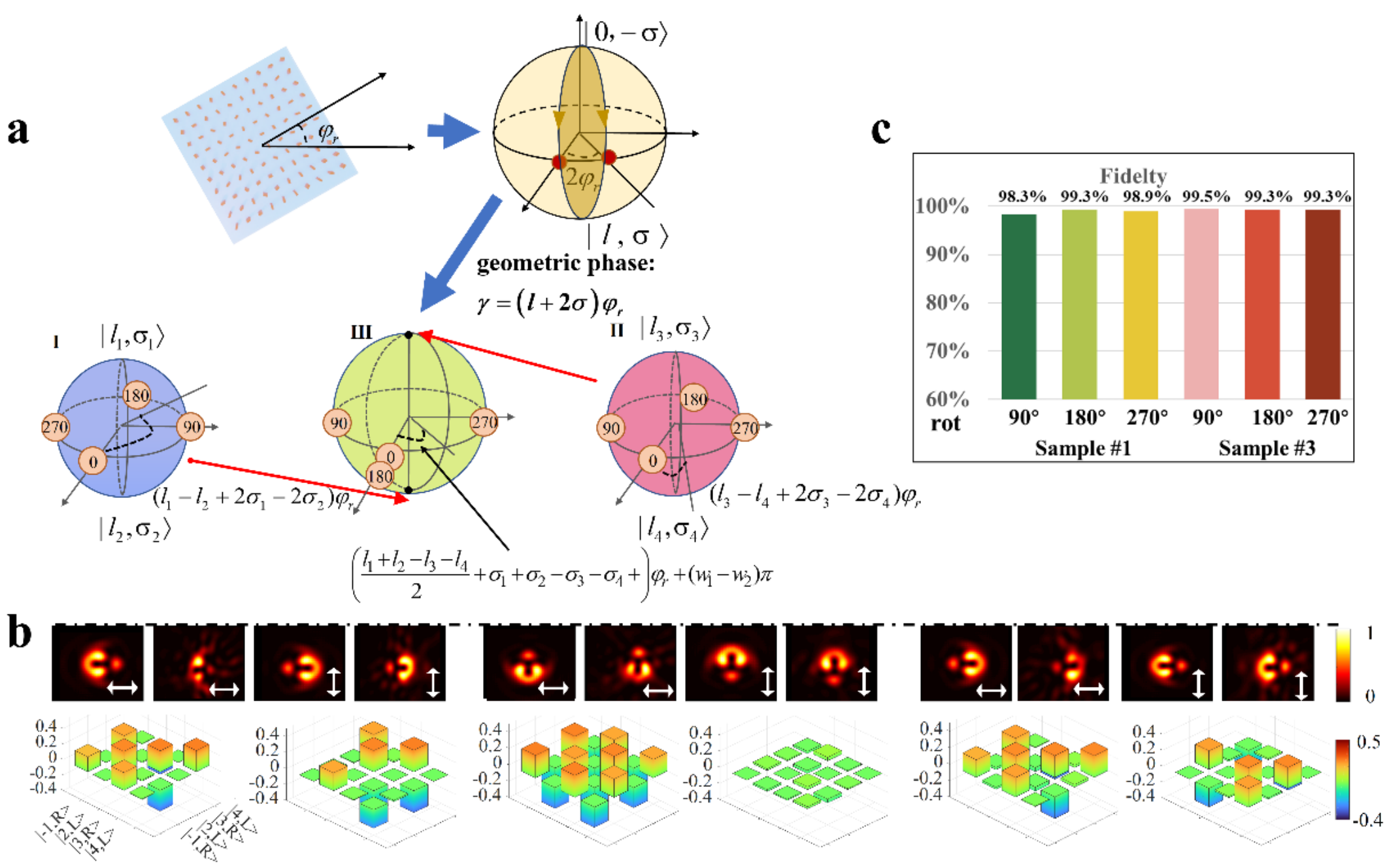

Fig.5. Higher order Pancharatnam−Berry (PB) phase controlled mode transformation.

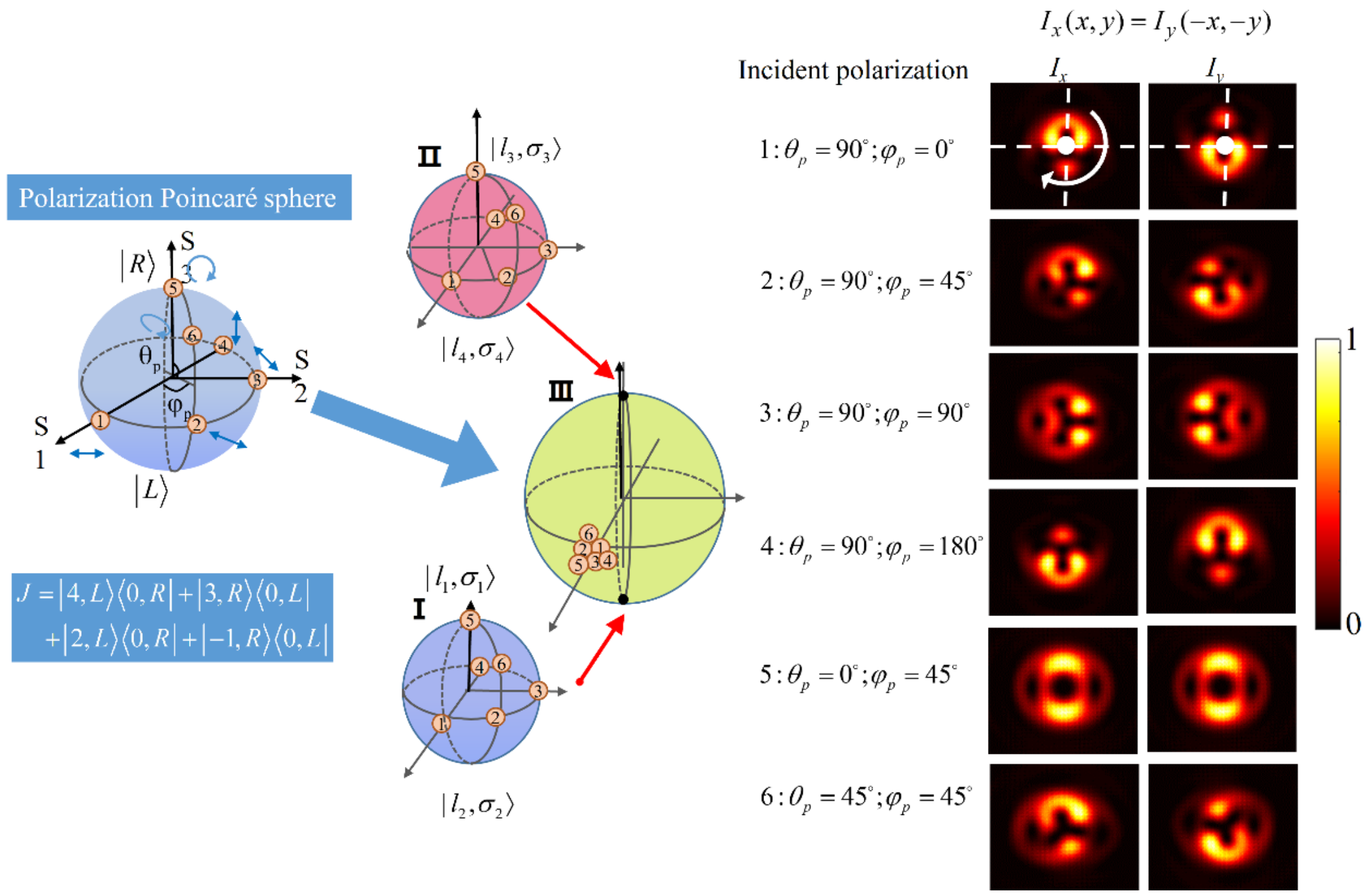

Figure S7 Far field results of sample # 3 under changing incident polarization state

Reviewer's Comments 10

The fonts in Figure 3 are too small.

Our Responses

We appreciate the reviewer's suggestion. We adjusted the layout of Figure 3 and enlarged the font size.

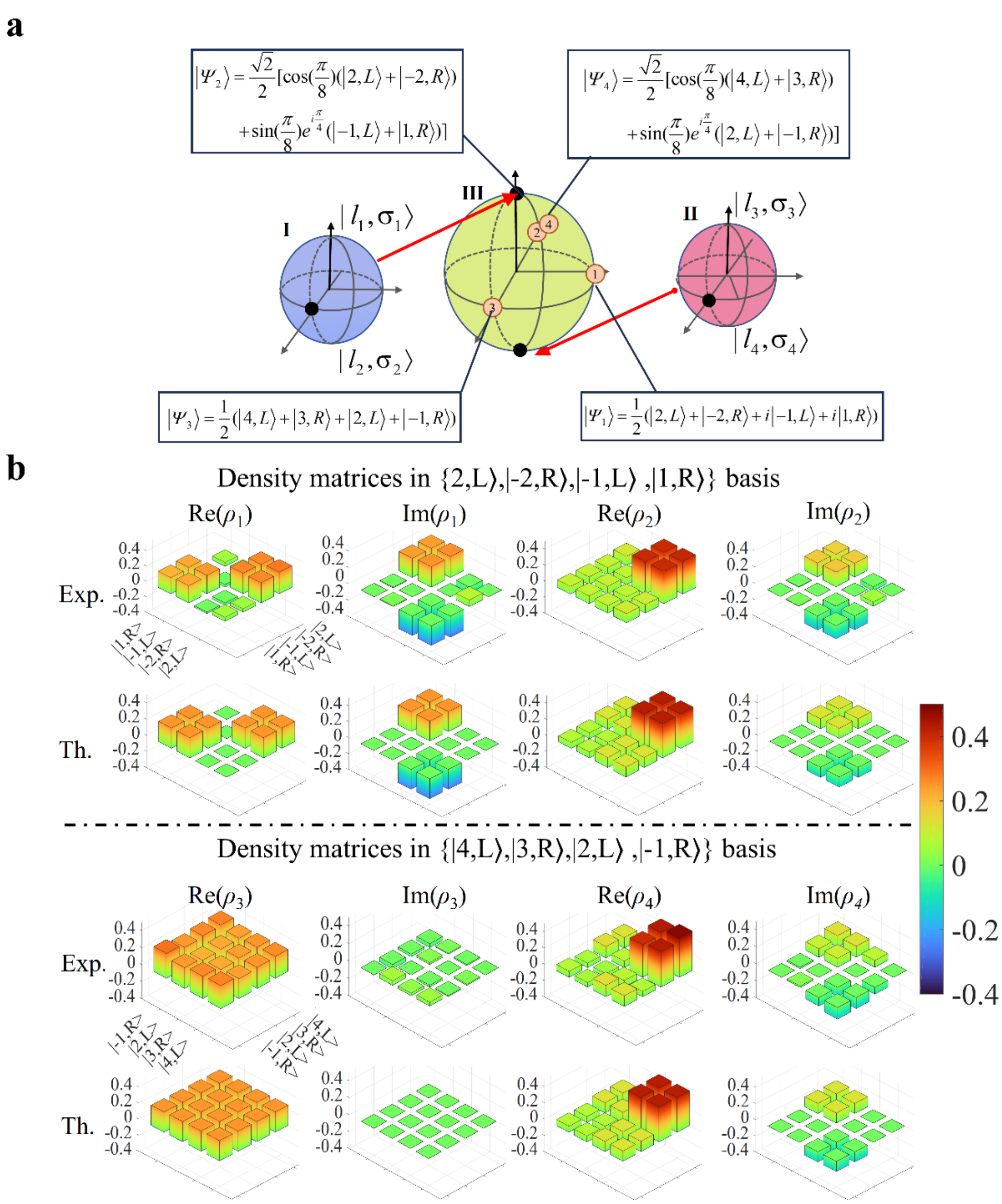

Fig.3. Experimental verification of designed metasurfaces.

Reviewer's Comments 11

In Figure 1c and Figure S6, the authors may consider including one reference axis and marking the corresponding rotation angles to help readers more easily identify the symmetries.

Our Responses

We appreciate the reviewer's comment. Following the reviewer's advice, we have added a reference axis and marked the corresponding rotation direction in Fig. 1c and

Figure S6(now Figure S7).

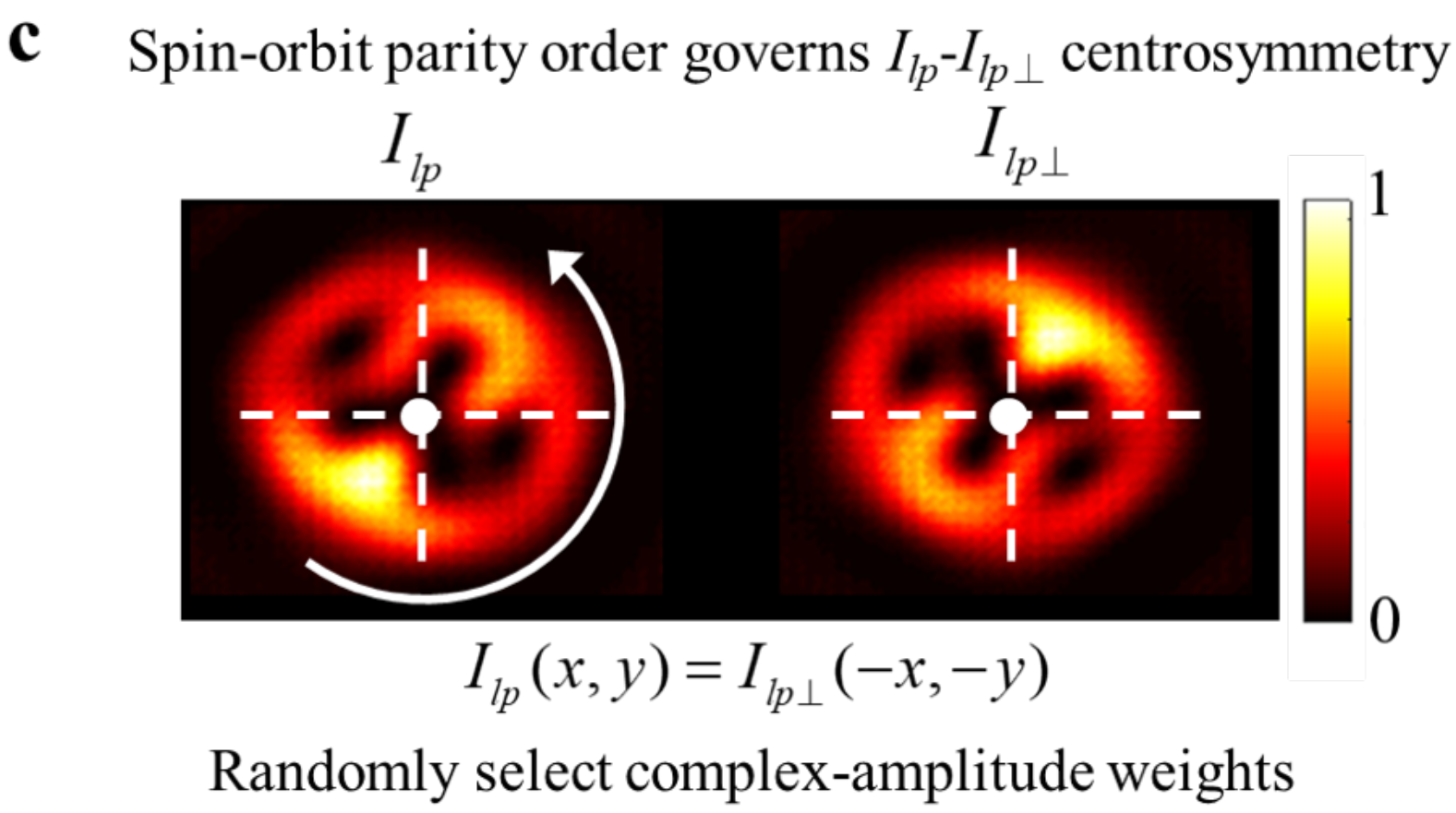

Fig.1.c, The concept of the spin–orbit parity order

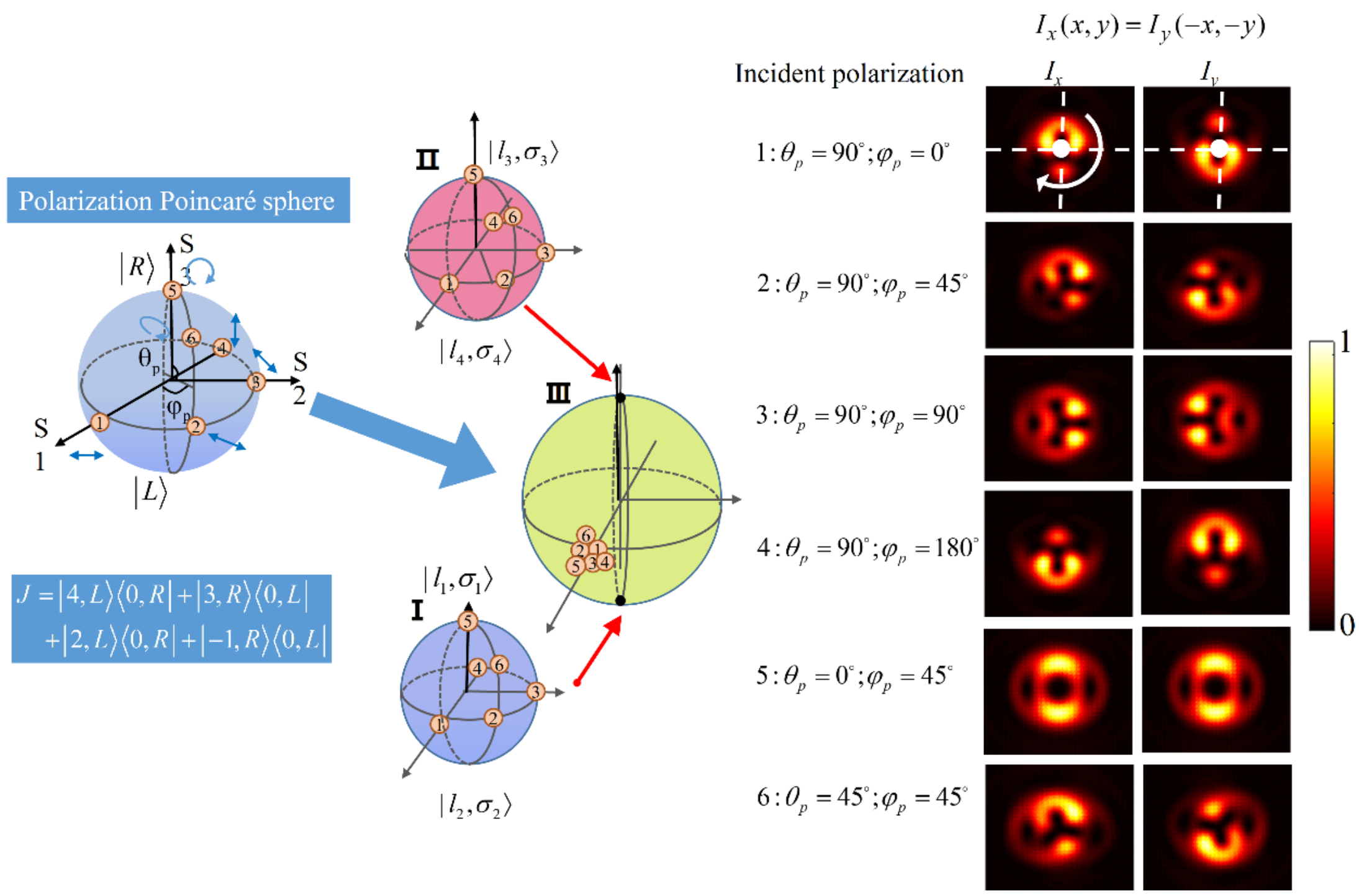

Figure S7 Far field results of sample # 3 under changing incident polarization state

REVIEWER 2

Reviewer's Comments

The authors demonstrate a metasurface for generating hybrid spin-orbit optical states in a four-dimensional Hilbert space, with scalability toward higher dimensionality. They further introduce an interferometric reconstruction scheme that

can efficiently characterize the full density matrix of these states experimentally. From a physics perspective, the work uncovers a symmetry invariant (spin-orbit parity order) and explores phenomena associated with geometric (topological) phases. Overall, the results are innovative, the physics is rigorous, and the presentation is generally clear. I recommend the manuscript for publication after minor revision.

Our Responses

We sincerely thank the reviewers for their positive comments and constructive suggestions. We have carefully addressed the issues raised and revised the manuscript accordingly.

Reviewer's Comments 1

My main question concerns, for metasurfaces designed following your strategy, how densely the generated states can span the parameter space (the Poincaré hypersphere), and how finely such states can be resolved and characterized experimentally.

Specifically, the four demonstrated examples correspond to points that are well separated on the Poincaré hypersphere. This raises the potential concern: when scaling the encoding strategy to higher dimensions, does the requirement of large separation imply that the encoding becomes highly discrete (although the total number of available states still scales exponentially)?

Our Responses

We thank the reviewer for this insightful question. The four examples shown in the manuscript were chosen as representative states for clearer visualization.

In principle, our method is not limited to a few isolated states. By encoding the target weights into the corresponding Jones matrix, the generated state can cover the whole Poincaré hypersphere. The large separation between the demonstrated examples is not a fundamental restriction of the encoding strategy.

In practice, the actual generated state $|\psi\rangle$ may deviate from the ideal target state $|\psi_0\rangle$ because of fabrication imperfections and experimental errors. For pure states, this

deviation can be quantified by the fidelity:

$$F = \left|\langle \psi | \psi_0 \rangle\right|^2 \tag{R4}$$

The higher the fidelity, the smaller the state deviation and the finer the experimentally resolvable spacing in the state space. If the minimum fidelity over the accessible parameter space is denoted by $F_{\min}$, the corresponding angular deviation may be expressed as:

$$\alpha = \arccos\sqrt{F_{\min}} \tag{R5}$$

This angle can be used to estimate the minimum state spacing that can be resolved experimentally on the Poincaré hypersphere. For the four-dimensional cases demonstrated in the manuscript, all reconstructed states exhibit fidelities above 98%. Taking $F_{\min} = 0.98$ gives $\alpha_{\max} \approx 8.13^\circ$. This indicates that, for the demonstrated samples, the experimentally generated states remain close to the target ones and are not limited to a few isolated points.

For higher-dimensional implementations, the main practical challenge is thus maintaining sufficiently high fidelity under fabrication and measurement errors. If the fidelity remains sufficiently high, the experimentally accessible states can still densely cover the corresponding parameter space.

Overall, how densely the states can be accessed in experiment mainly depends on the achieved fidelity.

Reviewer's Comments 2

You state that "Meanwhile, higher dimensionality can be realized by superposing orthogonal spin–orbit bases with different weights using a single metasurface that implements the corresponding Jones matrix. Hence, such method is universal and scalable."

This claim is strong. In practice, there are additional limitations on scalability (e.g., fabrication tolerances and cross-talk between modes). I would encourage you to discuss these limits and their implications for the metasurface design and the interferometric characterization experiment, potentially in the Supplementary

Information.

Our Responses

We thank the reviewer for this insightful comment. Following the reviewer's suggestion, we have added a new section, *"**Scalability to higher-dimension**"*, in the Supplementary Information to clarify both the scalability and its practical limitations.

In particular, we provide a numerical demonstration of an eight-dimensional spin–orbit state, confirming that the present strategy is extendable beyond the four-dimensional case. We further introduce fabrication perturbations to evaluate the practical robustness. The results show that, as the perturbation level increases, the generated far-field pattern and reconstructed density matrix gradually deviate from the target, indicating increased inter-mode crosstalk. Correspondingly, the fidelity decreases from 99.5% for the ±5 nm perturbation to 98.1% for the ±10 nm perturbation, while remaining relatively high in both cases.

We also discuss possible strategies to improve the metasurface design and the interferometric characterization system for more robust generation and measurement of higher-dimensional states under practical conditions.

"The Jones-matrix-based framework used in this work is not restricted to the four-dimensional case. In principle, higher-dimensional spin–orbit states can be generated by incorporating additional orthogonal spin–orbit eigen-components with prescribed complex weights into the target Jones matrix. To illustrate this scalability, we numerically designed a representative eight-dimensional spin–orbit state:

$$|\Psi\rangle = \frac{1}{2\sqrt{2}}(|4,L\rangle + |3,L\rangle + |2,L\rangle + |-1,L\rangle + |4,R\rangle + |3,R\rangle + |2,R\rangle + |-1,R\rangle) \quad \text{(S42)}$$

Figure S9a presents the theoretical far-field intensity under x-polarized illumination and x-polarized projection, and Figure S9b shows the corresponding calculated result after mapping the ideal continuous design onto a discrete practical meta-atom library. The good agreement between the two is further supported by the density matrix shown in Figure S10, where the calculated density matrix in Figure S10b closely matches the theoretical target in Figure S10a. The corresponding fidelity reaches 99.9%, confirming that the present Jones-matrix-based design strategy can be extended beyond the four-

dimensional Hilbert space demonstrated in the main text.

To evaluate the practical scalability, we further introduced fabrication-related perturbations into the metasurface design. Specifically, for each pixel, the length and width of the meta-atom are independently perturbed by random values chosen with equal probability from{-5nm，0，+5nm} and{-10nm，-5nm，0，+5nm，+10nm}.When increasing perturbation level, the generated far-field electromagnetic field shows increasing deviation from the target state, as shown in Figure S9c, d. The similar trend is observed in the reconstructed density matrices in Figure S10c, d. From the density matrices, the real part progressively departs from the theoretical distribution, while non-ideal imaginary components emerge, indicating increased inter-mode crosstalk. Accordingly, the state fidelity decreases to 99.5% for the ±5 nm perturbation case, and 98.2% for the ±10 nm perturbation case. Nevertheless, the fidelity remains high in both cases, indicating that the proposed design retains good robustness against moderate fabrication perturbations.

In summary, as the target Hilbert-space dimensionality increases, a larger number of spin–orbit eigen-components must be simultaneously controlled with accurate relative amplitudes and phases. This places stringent requirements on the match between the fabricated meta-atom response and the ideal design, making higher-dimensional state generation more sensitive to fabrication deviations and near-field coupling between meta-atoms. Such limitations may be alleviated in several ways. For example, one may employ meta-atoms with improved fabrication tolerance. In addition, hybrid anapole meta-atoms may help suppress near-field interactions. More generally, inverse design methods that jointly incorporate fabrication uncertainty, near-field coupling, and target state fidelity could provide a practical route toward more robust high-dimensional spin-orbit state design.

On the characterization side, higher-dimensional states generally involve finer spatial features and broader spatial frequency, which impose stricter requirements on the sampling bandwidth of the off-axis interferometric system. In practice, to ensure reliable frequency separation in the Fourier plane, the sensor sampling bandwidth should be at least four times larger than the object bandwidth.”

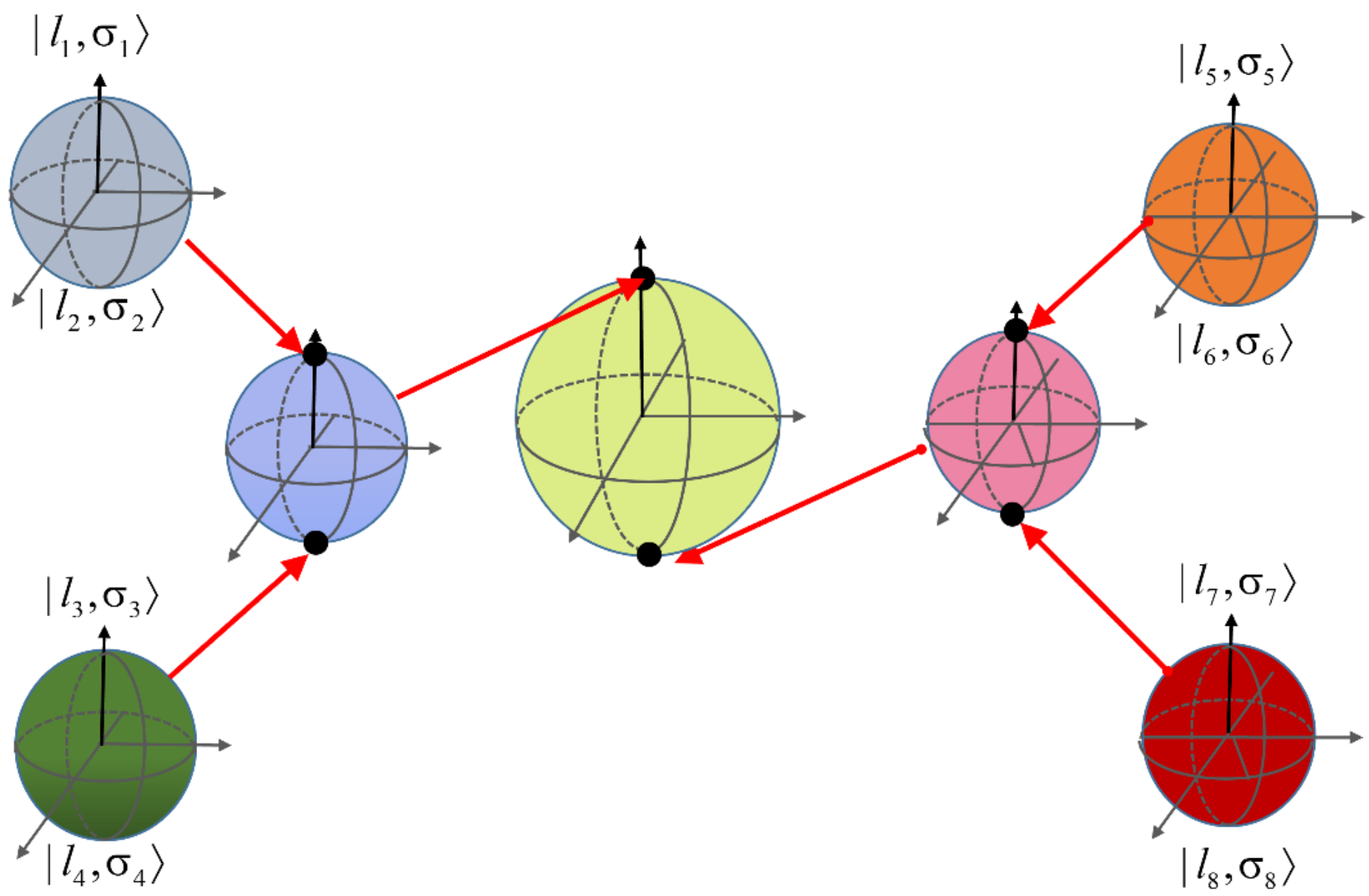

Figure S8 Poincaré hypersphere representation of the eight-dimensional spin-orbit Hilbert space.

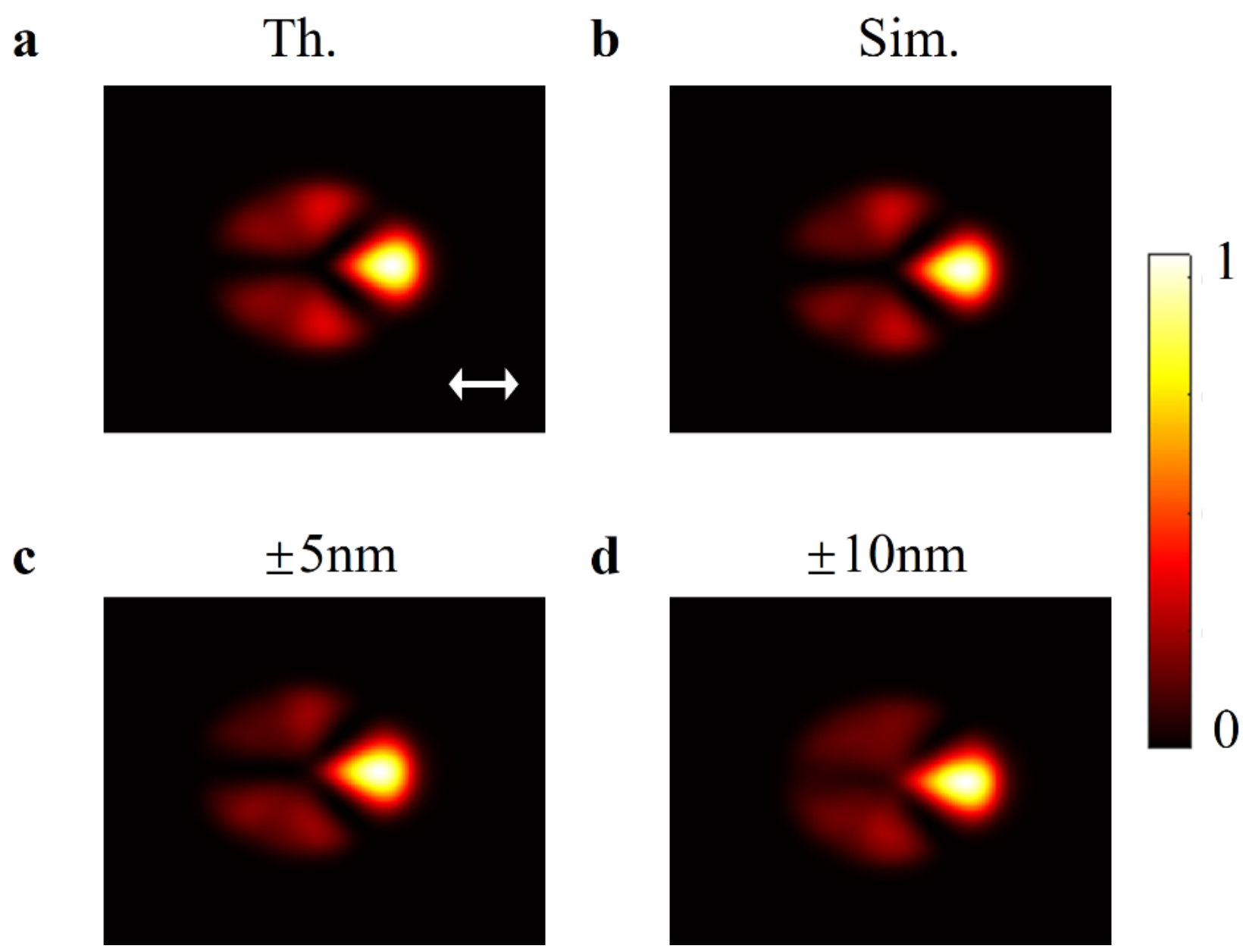

Figure S9 Far-field intensity of the eight-dimensional spin–orbit state under x-polarized projection. **a.** Theoretical result of the target state. **b.** Corresponding calculated result. **c, d.** Calculated results with fabrication perturbations, where the length and width of each meta-atom are independently and randomly varied with equal probability from {−5 nm,0, +5 nm}in c and {−10 nm, −5 nm, 0, +5 nm, +10 nm}

in d, respectively.

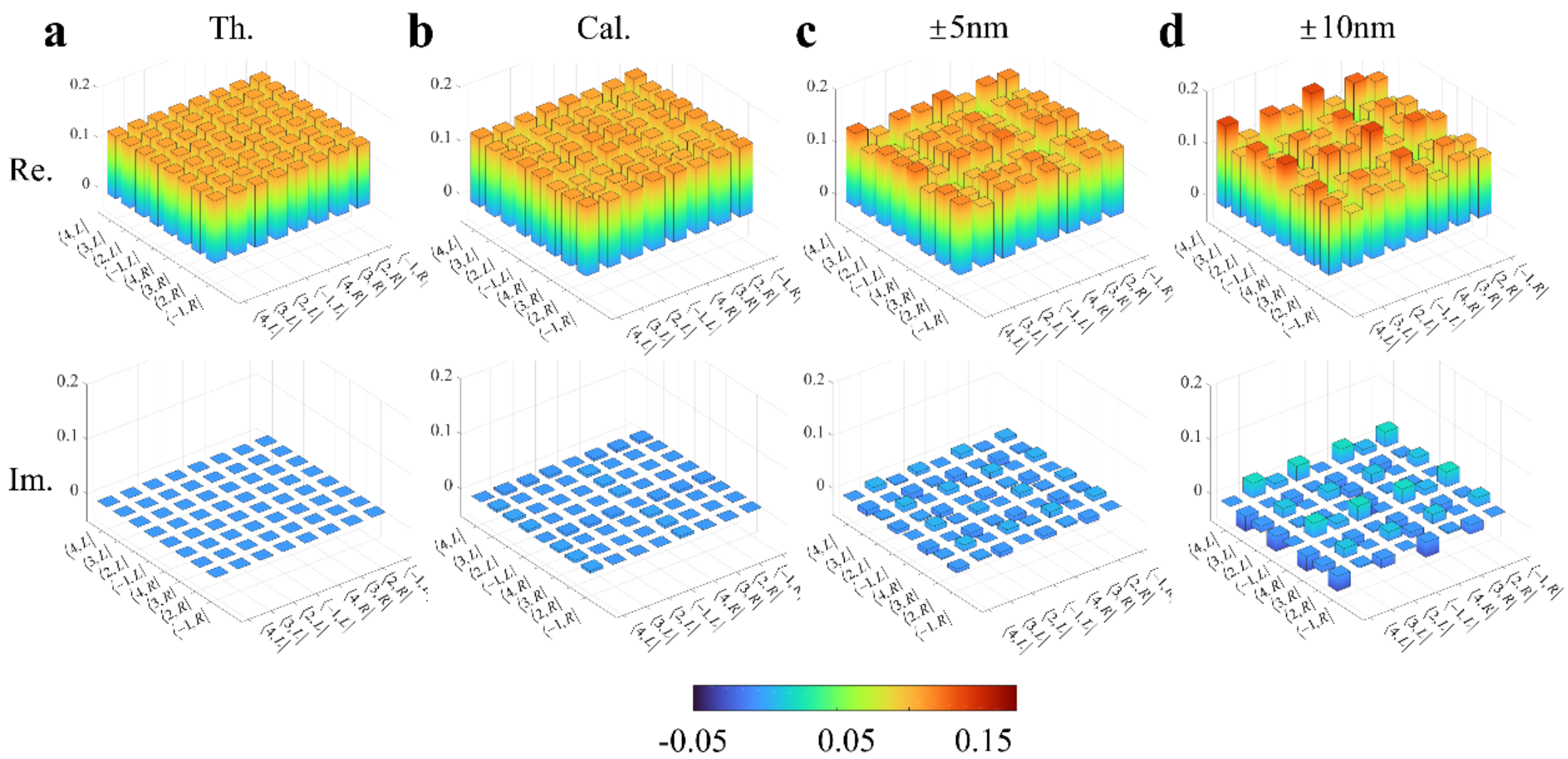

Figure S10 Density matrix results for the eight-dimensional spin–orbit state. **a.** Theoretical density matrix. **b.** Calculated density matrix. **c., d.** Calculated density matrices under fabrication perturbations corresponding to Figure S9 c, d, respectively.

Reviewer's Comments 3

In Fig. 1b, what does "Ref." above the cube refer to? Please revise the figure.

Our Responses

We appreciate the reviewer's suggestion. The "Ref" refers to the reference light in the interferometric system. We have redrawn the Fig.1.

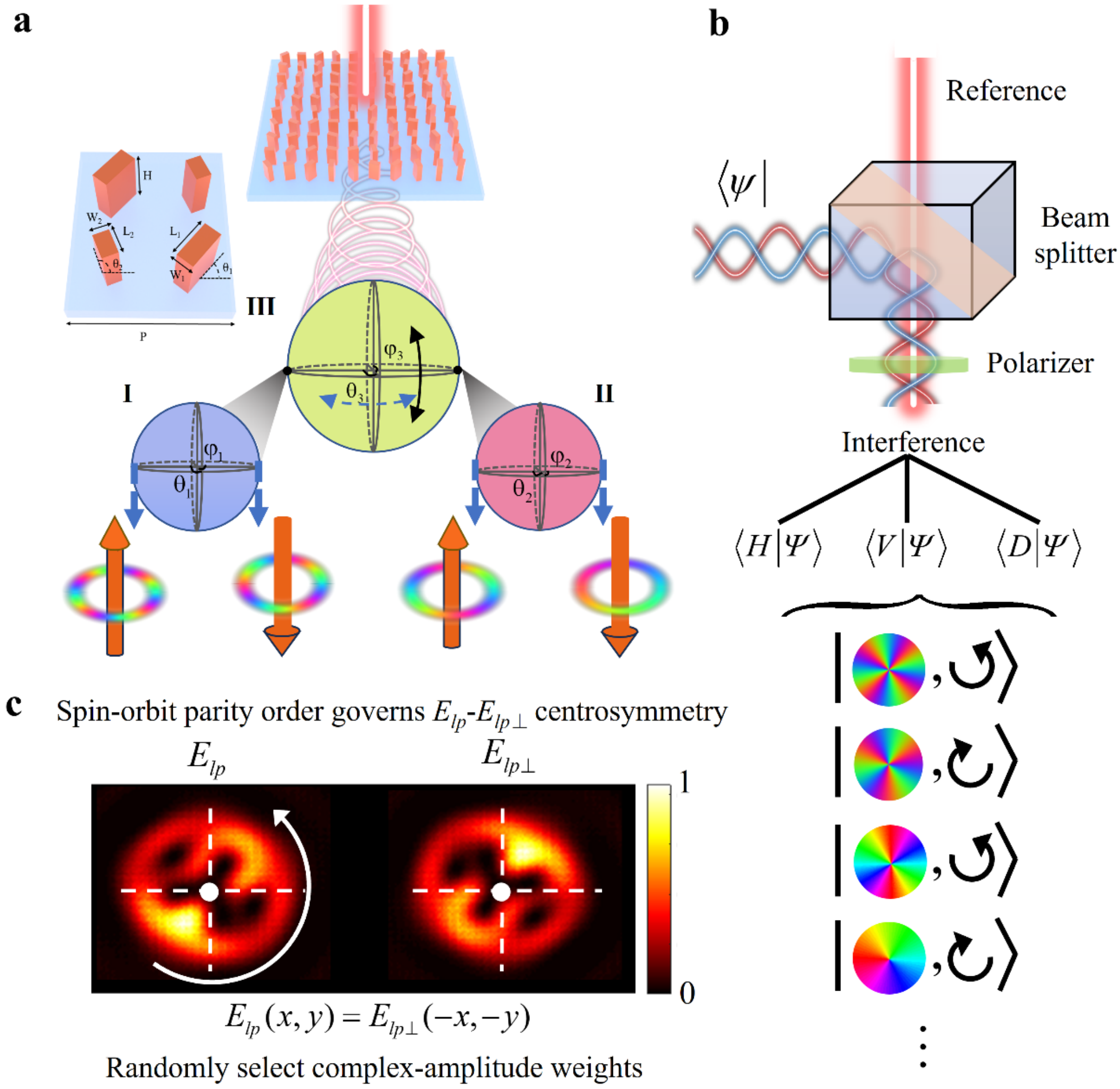

Fig.1. Generation, detection and parity order of high-dimensional spin–orbit states.

Reviewer's Comments 4

The manuscript structure does not comply with Nature Communications formatting: section titles such as "Principle" needs to be revised to match journal requirements.

Our Responses

We thank the reviewer for this valuable suggestion. We have revised the section titles to better match the formatting requirements of *Nature Communications*. Specifically, we added a "Results" section after the Introduction, and changed "Principle" together with the previous first-level headings into subsection headings under "Results".

Reviewer's Comments 5

Some wording choices are unnecessarily uncommon (e.g., "nascent"). Please replace these with more standard scientific language to improve readability.

Our Responses

We thank the reviewer for this helpful comment. We have carefully revised the manuscript to replace unnecessarily uncommon wording choices in order to improve readability. The corresponding changes made in the revised manuscript are listed below.

1."*High-dimensional optical fields are poised to advance next-generation high-capacity, robust communication and computing technologies*."

→"High-dimensional optical fields are promising for advancing next-generation high-capacity, robust communication and computing technologies."

2."*Direct generation of arbitrary hybrid spin-orbit state in high-dimensional Hilbert space with metasurface still remain nascent*"

→"*Direct generation of arbitrary hybrid spin-orbit state in high-dimensional Hilbert space with metasurface is still at an early stage*"

3. " *Complete characterization of high-dimensional spin–orbit states underpins emerging photonic technologies and fundamental studies*"

→"*Complete characterization of high-dimensional spin–orbit states is essential for emerging photonic technologies and fundamental studies*"
